# Flavonoids in Nasal Therapeutics: Biological Activities, Delivery Challenges, and Formulation Strategies–A Systematic Review

**DOI:** 10.1002/ptr.70380

**Published:** 2026-05-17

**Authors:** Jeniffer Viviana Ramirez Hernández, Sara Elis Bianchi, Flávia Nathiely Silveira Fachel, Elizandra Braganhol, Sheila Porto de Matos, Valquíria Linck Bassani

**Affiliations:** ^1^ Programa de Pós‐Graduação Em Ciências Farmacêuticas Universidade Federal Do Rio Grande Do Sul Porto Alegre RS Brazil; ^2^ Programa de Pós‐Graduação Em Biociências Universidade de Ciências da Saúde de Porto Alegre Porto Alegre RS Brazil

**Keywords:** administration routes, flavonoids, nasal administration, nose‐to‐brain, respiratory diseases

## Abstract

Flavonoids represent a significant group of secondary metabolites. They have gained significant attention in the field of health due to their wide range of chemical structures and their diverse biological activities. These compounds have been most commonly administered orally as sugar conjugates (heterosides). Flavonoid nasal administration emerges as a therapeutic strategy, with potential for local, systemic, or targeted flavonoid actions, mainly in the free form (aglycones). This study provides a systematic and comprehensive review of flavonoid classes and administration routes, emphasizing their roles at three levels of the nasal administration route. Following PRISMA guidelines, a systematic search was conducted in Web of Science and PubMed (December 1997–November 2024), retrieving 225 records, of which 40 met the inclusion criteria. The first one is in the nasal cavity, highlighting its beneficial effects on the nasal mucosal epithelia (topical effect); the second is the flavonoid administration for treating naso‐respiratory and other diseases, and the third is how the flavonoid can be targeted to the brain, taking advantage of the nose‐to‐brain route to prevent or treat CNS diseases.

## Graphical abstract

Overview of the therapeutic potential of flavonoids in nasal delivery systems, highlighting their benefits to the human nasal cavity, and applications in the treatment of respiratory and brain‐related diseases.

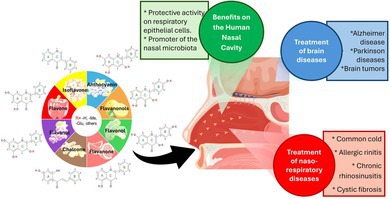

**Abbreviations**
3OMQ: 3‐O‐methylquercetin
6‐OHDA: 6‐hydroxydopamine
ANO1: calcium‐activated chloride channel anoctamin‐1
ANR: anthocyanidin reductase
ANS: anthocyanidin synthase
BALB/c: albino strain of common laboratory mouse
BBB: blood–brain barrier
BEAS‐2B: normal bronchial epithelial cells
C57/BL6: inbred strain of common laboratory mouse
Calu‐3: human lung cancer cell line
cAMP: cyclic adenosine monophosphate
CAT: catalase
CBF: ciliary beat frequency
CF: cystic fibrosis
CFBE41o−: cystic fibrosis human bronchial epithelial cell line
CFTR: cystic fibrosis transmembrane conductance regulator
CGRP: calcitonin gene‐related peptide
CHI: chalcone isomerase
CHS: chalcone synthase
CNS: central nervous system
CRS: chronic rhinosinusitis
CRSsNP: chronic rhinosinusitis without nasal polyps
CRSwNP: chronic rhinosinusitis with nasal polyps
DFR: dihydroflavonol 4‐reductase
DMBT1: deleted in malignant brain tumors 1
DNA: deoxyribonucleic acid
ECM: extracellular matrix
ECP: eosinophil cationic protein
EGCG: epigallocatechin gallate
ELISA: enzyme‐linked immunosorbent assay
ER: endoplasmic reticulum
F3H: flavanone 3‐hydroxylase
F3βH: flavanone 3β hydroxylase
∆F508 CFTR: deletion of phenylalanine at position 508 of the cystic fibrosis transmembrane conductance regulator
FDA: Food and Drug Administration
FLS: flavonol synthase
FNS: flavone synthase
G551D: nonsense mutation of glycine to aspartic acid at codon 551
GATA3: GATA binding protein 3
GPx: glutathione peroxidase
GSH: glutathione
H1R: histamine H1 receptor
HMC‐1: human mast cell line
HNEpC: human nasal epithelial cells
HO‐1: heme oxygenase‐1
HPβCD: hydroxypropyl‐β‐cyclodextrin
HSNE: human sinonasal epithelium
IFN‐γ: interferon gamma
IFS: isoflavone synthase
IgE: immunoglobulin E
IgG1: immunoglobulin G1
IKKβ: inhibitory kappa B kinase beta
iNOS: inducible nitric‐oxide synthase
IκBα: inhibitor of nuclear transcription factor kappa B alpha
JAK2: janus kinase 2 gene
L929: mouse fibroblast cell line
L‐DOPA: dopamine and L‐3,4‐dihydroxyphenylalanine
LID: L‐DOPA induced dyskinesia
LPH: phlorizin hydrolase
LPS: lipopolysaccharide
MAPK: mitogen‐activated protein kinase
MDA: malondialdehyde
MeβCD: methyl‐β‐cyclodextrin
miR‐21: mammalian microRNA
MLMP: mannitol/lecithin microparticles
MNSE: urine nasal septal epithelium
mRNA: messenger RNA
MUC5AC: gen de mucina MUC‐5AC
MyD88: myeloid differentiation factor 88
NALT: nasopharyngeal‐associated lymphoid tissue
NAR‐NPs: chitosan nanoparticles loaded with naringin
NF‐κB: nuclear transcription factor kappa B
NGF: nerve growth factor
NO: nitric oxide
Nrf2: erythroid nuclear factor 2‐related factor 2
OVA: ovalbumin
PKA: protein kinase A
PKC: protein kinase C
p‐STAT3: phosphorylated signal transducer and activator of transcription 3
p‐STAT6: phosphorylated signal transducer and activator of transcription 6
RNA: ribonucleic acid
ROR‐γ: RAR gamma
ROS: reactive oxygen species
RT‐PCR: reverse transcriptase polymerase chain reaction
SGLT1: sodium‐dependent glucose transporter 1
SIRT1: silent information regulator protein 1
SOCS1: suppressors of cytokine signaling 1
SOD: superoxide dismutase
SP: substance P
STAT3: signal transducer and activator of transcription 3
STAT5: signal transducer and activator of transcription 5
STAT6: signal transducer and activator of transcription 6
T2R14: a bitter taste receptor of family type 2 receptors, or T2Rs
T‐bet: T‐box protein expressed in T cells
TDI: toluene 2,4‐diisocyanate
TGF‐β1: transforming growth factor β1
TLR4: toll‐like receptor 4
TNF‐α: tumor necrosis factor alpha
TSLP: thymic stromal lymphopoietin
WB: Western blot
WoS: Web of Science
βG: β‐glucosidase

## 1 Introduction

The nose serves multiple functions, including warming, humidifying, filtering inhaled air, and olfaction (Türker et al. 2004). Four regions characterize the internal structure of the nasal cavity: the nasal vestibule and the respiratory, olfactory, and nasopharyngeal regions. The nasal vestibules are in the anterior part of the nasal cavity and primarily serve as a deflecting system, filtering the air and removing foreign substances, including drugs (Jeong et al. 2023; Türker et al. 2004). The respiratory region is the largest area of the nasal cavity and is highly vascularized. It contributes to multisensory processing and respiration (Jeong et al. 2023; Rassu et al. 2021). The olfactory region, situated at the upper part of the nasal cavity near the olfactory bulb, is crucial for olfactory perception by allowing access to central olfactory sensory neurons (Jeong et al. 2023; Rassu et al. 2021). Finally, the nasopharyngeal region, located at the back of the nasal cavity, contains the lymphatic system known as the nasopharyngeal‐associated lymphoid tissue (NALT), an immunological organ that blocks the invasion of foreign pathogens into the nasal cavity and upper respiratory tract (Jeong et al. 2023). These anatomical and physiological characteristics make the nasal cavity a particularly attractive route for drug administration, not only for local and systemic applications but also for targeting the central nervous system.

On the other hand, flavonoids are polyphenolic compounds that form a group of secondary metabolites in plants. They are distributed across fruits, vegetables, herbs, stems, cereals, nuts, flowers, and seeds of various species. Over 10,000 plant‐derived flavonoid compounds have been recorded (S. Chen et al. 2023; Shen et al. 2022). These compounds are highly valued in health for their wide range of biological activities. They can regulate key cellular enzymatic functions, showing beneficial properties such as anti‐inflammatory, anticancer, anti‐aging, antimutagenic, and antioxidant. Moreover, flavonoids exhibit promising biochemical effects across a variety of conditions, including cardiovascular diseases, atherosclerosis, and neurodegenerative disorders (S. Chen et al. 2023; Shen et al. 2022).

Nevertheless, despite their considerable pharmacological potential, the clinical implementation of flavonoids is limited by unfavorable biopharmaceutical properties. In general, these compounds exhibit low aqueous solubility, poor absorption, and rapid gastrointestinal and hepatic first‐pass metabolism, which represent significant barriers to their therapeutic application across a wide range of diseases (Bicker et al. 2020; S. Chen et al. 2023; Kokkinis et al. 2024). However, it is necessary to distinguish the flavonoid conjugates (flavonoid heterosides) from aglycones. The former represent the predominant form in plant tissues and are generally more water‐soluble. In contrast, aglycones are the non‐glycosylated forms, typically generated through enzymatic hydrolysis of glycosides and usually present in lower amounts as free compounds in plant material.

As a consequence of these physicochemical differences, the absorption of both forms differs significantly. In the context of oral administration, flavonoid aglycones have been reported to enter intestinal epithelial cells primarily via passive diffusion, owing to their greater lipophilicity. In contrast, flavonoid glycosides generally require hydrolysis in the intestinal lumen or within the epithelium prior to absorption (Cassidy and Minihane 2017). The lactase phlorizin hydrolase is responsible for the hydrolysis of the flavonoid glycosides (A. J. Day et al. 2000). However, this enzyme is expressed exclusively in the small intestine (Troelsen et al. 1997). To the best of our knowledge, there is no evidence that flavonoid heterosides are enzymatically hydrolyzed in the nasal mucosa.

Historically, the nasal route was primarily used to treat local sinonasal conditions, establishing itself as one of the preferred pathways for managing respiratory diseases (Bicker et al. 2020; Lagoa et al. 2020; Papakyriakopoulou et al. 2021; Shrewsbury 2023). Nevertheless, due to the nasal mucosa's high vascularization and permeability, unintended systemic absorption can occur. Far from being a limitation, this characteristic has sparked significant pharmacological interest, enabling its exploration for the treatment of various diseases, including those that do not directly involve the nasal cavity (Grassin‐Delyle et al. 2012; Shrewsbury 2023).

Compared to conventional systemic administration methods, such as oral or parenteral routes, nasal delivery offers several key advantages: ease of administration due to its non‐invasive nature, rapid onset of action, reduced enzymatic activity, high absorption area, the possibility of using lower doses, and the ability to bypass the blood–brain barrier (BBB) through the trigeminal nerve or olfactory bulb (Cingi et al. 2021; Hou et al. 2023; Keller et al. 2022). This feature is particularly relevant because the BBB also contains active efflux transporter systems, such as P‐glycoprotein (P‐gp) and breast cancer resistance protein (BCRP), which limit the penetration of many circulating compounds into the central nervous system (Abbott 2013; Crowe et al. 2018; Illum 2000; Pandit et al. 2020; Selvaraj et al. 2018).

However, the effectiveness of these therapeutic effects is determined by the major barriers that limit the complete absorption of a drug. To achieve local action, the drug must rapidly pass through the mucus layer, followed by the epithelial cell membrane, and finally the stroma and basal membrane. For drugs intended for systemic action, they must overcome an additional fourth barrier: the capillary endothelium (Tai et al. 2022). In addition, several factors, such as mucociliary clearance, the presence of ciliated cells, local enzyme activity, and physical barriers, including the BBB, pose additional challenges to the efficient transport of drugs via the nose‐to‐brain delivery route (Marcello and Chiono 2023). These barriers pose significant challenges, as they impose limitations on formulation design and raise toxicological concerns that need to be optimized, particularly in systemic administration approaches (Keller et al. 2022).

Despite these challenges, the close anatomical proximity of the nasal cavity to the central nervous system provides a unique opportunity for direct nose‐to‐brain drug delivery. This pathway can allow the transport of bioactive compounds through multiple mechanisms, including: (1) transport across the olfactory epithelium, allowing the drug to reach the olfactory bulb; (2) absorption through the respiratory epithelium, followed by access to the brain via the systemic circulation; and (3) transport mediated by branches of the trigeminal nerve, which project directly into the central nervous system (Chung et al. 2023; Keller et al. 2022; Shrewsbury 2023).

For clinical and commercial applications, the development of nasal formulations must adhere strictly to the regulatory frameworks established by agencies such as the U.S. Food and Drug Administration (FDA) and the European Medicines Agency (EMA). These agencies define in detail the development stages, quality criteria, and safety requirements that must be met for authorization, clinical use, and subsequent commercialization of new therapeutic agents, directly influencing excipient selection, delivery systems, and quality control strategies (European Medicines Agency 2025; Food and Drug Administration 2016).

In this context, this review critically analyzes and synthesizes the various delivery systems developed for the nasal administration of flavonoids, addressing local, systemic, and direct nose‐to‐brain therapeutic applications. Furthermore, it examines the key physiological, biopharmaceutical, and regulatory challenges associated with this route of administration, aiming to provide a comprehensive overview of the therapeutic potential of intranasally administered flavonoids.

## 2 Systematic Search Methodology

A systematic review was conducted following PRISMA guidelines, using two electronic databases: Web of Science (WoS) and PubMed. These databases were selected due to their comprehensive coverage of high‐impact journals in the fields of health sciences and biomedical research, ensuring the retrieval of relevant and high‐quality literature.

The search was performed using Boolean operators and the following keyword combinations:

- “flavonoid*” AND “nasal”
- “flavonoid*” AND “nose”

The asterisk (*) was used as a truncation symbol to capture word variations (e.g., flavonoid, flavonoids). The search was restricted to studies published in English between December 31, 1997 and November 10, 2024.

### 2.1 Inclusion Criteria

Studies were included if they met all the following conditions:

- Investigated the biological activities of flavonoids in relation to diseases or disorders of the nasal cavity.
- Explored the intranasal administration of flavonoids.
- Evaluated the use of cyclodextrins to enhance the nasal absorption of flavonoids.
- They were original research articles (case studies were included if relevant).

### 2.2 Exclusion Criteria

- Articles not available in full text.
- Duplicates across databases.
- Reviews, editorials, letters, or studies unrelated to nasal delivery or flavonoid activity.

### 2.3 Selection Process

The database search retrieved a total of 225 records (71 from WoS and 154 from PubMed). After the removal of 6 duplicates and 2 records with inaccessible full texts, 217 articles were screened. The selection followed a two‐phase process:

1. Title and abstract screening to eliminate studies clearly outside the scope.
2. Full‐text review to assess compliance with the inclusion criteria.

**FIGURE 1:**
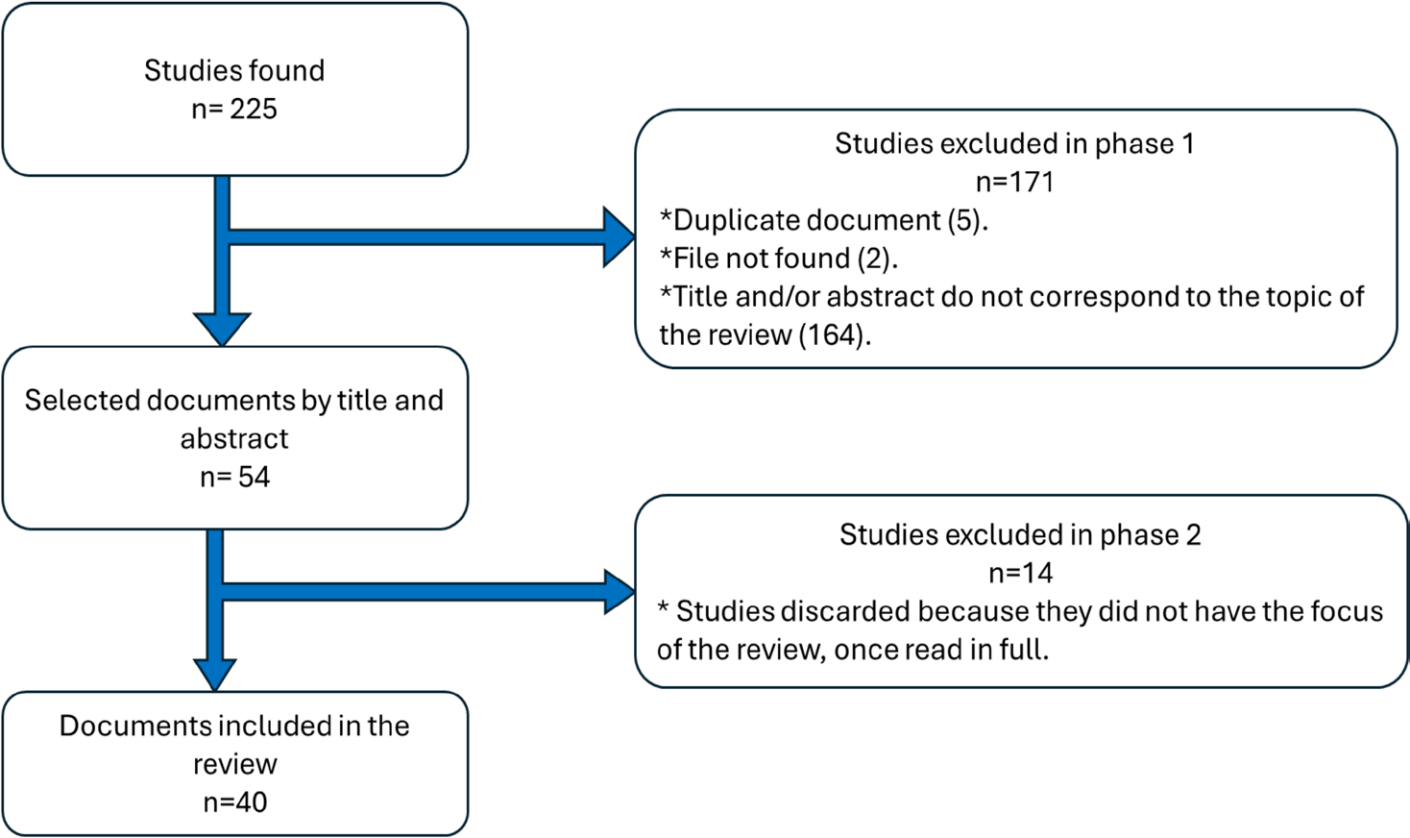
Flowchart of document selection for the review and inclusion/exclusion criteria (from Dec/1997 to Nov/2024).

After full‐text assessment, 177 articles were excluded. A total of 40 studies met all the eligibility criteria and were included in the final review. The detailed selection process is summarized in Figure 1 (flowchart of article inclusion).

## 3 Chemical Properties and Classification of Flavonoids

**FIGURE 2:**
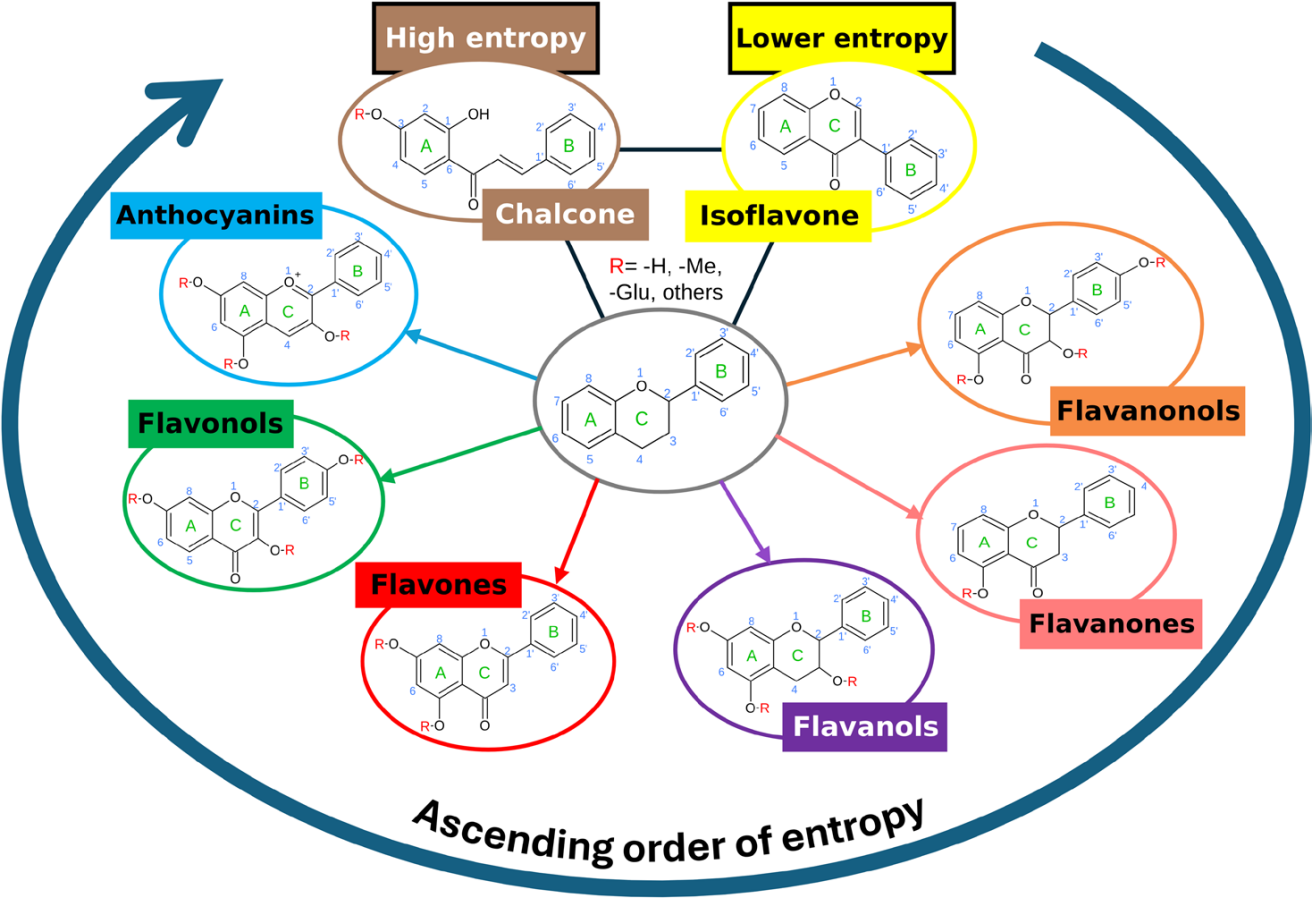
Basic structure and classification of flavonoids.

Flavonoids are secondary metabolites of plants coming from the phenylpropanoid metabolic pathway (Berga et al. 2023; S. Chen et al. 2023). The basic skeleton of these compounds is characterized by having 15 carbon atoms arranged in three rings: A, C, and B (C6–C3–C6), as shown in Figure 2. They are usually found in nature bound to sugars, such as the β‐glucosides, which generally improve their water solubility, stability, and bioavailability (Berga et al. 2023; S. Chen et al. 2023; Dahiya et al. 2023). According to the position of the B ring as a substitution group of the C ring, the degree of unsaturation and oxidation of the heterocyclic central ring, they can be classified into eight groups: isoflavonoids, flavones, flavonols, flavanones, flavanols, flavanonols, chalcones and anthocyanins (Berga et al. 2023; Panche et al. 2016; Ramesh et al. 2021). Figure 2 shows their general chemical structure and the corresponding entropy. The positions of hydroxyl groups in the A and B rings, and their presence or absence in the C ring, lead to various types of flavonoids (Shen et al. 2022).

### 3.1 Flavones

Flavones are one of the most abundant classes of flavonoids. Its chemical structure consists of a 4H‐chromen‐4‐one with a phenyl substituent at position 2 (Shen et al. 2022). They are characterized by low water solubility and low bioavailability (Berga et al. 2023). The variations within flavone structures, hydroxyl groups, *O*‐methylation, and acylation at specific positions make this subclass a group with intermediate‐high entropy (Castellano et al. 2013; Ku et al. 2020). From a biological point of view, these substituents enable the flavones to act as effective xanthine oxidase inhibitors and exhibit strong superoxide‐scavenging capacity. Additionally, synthetic prenylated flavone derivatives have a high potential for blood vessel relaxation due to their antioxidant effects and ability to modulate nitric oxide (NO) (Cos et al. 1998; X. Dong et al. 2008).

This subclass of flavonoids can be found in celery, parsley, red peppers, chamomile, mint, tea, oranges, and ginkgo, which tend to accumulate in plant leaves and flowers (S. Chen et al. 2023; Ku et al. 2020; Shen et al. 2022). Glycosylation can occur in two forms: *O*‐glycosylation (at positions 7, 3′, and 4′) and *C*‐glycosylation (at positions 6 and 8) (Ku et al. 2020). Some examples of flavones include apigenin, chrysin, luteolin, vitexin, isovitexin, and tangeretin (S. Chen et al. 2023; Kopustinskiene et al. 2020; Shen et al. 2022).

### 3.2 Flavonols

Flavonols, also known as 3‐hydroxyflavones. Besides the presence of hydroxyl groups in the A and B rings, their chemical structure is characterized by a hydroxyl or methoxy group at C3 of the 4H‐chromen‐4‐one (C ring) (S. Chen et al. 2023; Kopustinskiene et al. 2020; Shen et al. 2022). The *C*‐glycoside form of flavonols is rare, with most occurring as aglycones or *O*‐glycosides (Ku et al. 2020). Their wide range of biological activities has been related to the presence of —OH groups. For example, flavonols have hydroxyl groups at positions 5 and 7 of the A ring, primarily in protecting DNA from UV‐induced damage in epidermal cells (Kopustinskiene et al. 2020; Shen et al. 2022). They are also highly antioxidant due to their ability to bind and stabilize free radicals (Berga et al. 2023; Cos et al. 1998).

These compounds have high structural entropy due to a hydroxyl group at position 3 of the C ring and to the wide range of possible substitutions. They show higher water solubility than other flavonoid subclasses (Berga et al. 2023; Castellano et al. 2013). Prenylated flavonols exhibit potent antioxidant and vasorelaxant activity, possibly due to their action on enzymes related to NO synthesis and their ability to inhibit the production of reactive oxygen species (ROS) (X. Dong et al. 2008).

From a biological perspective, flavonols have demonstrated antioxidant, antibacterial, cardioprotective, anticancer, and antiviral effects. Preventive effects against gastric cancer risk, especially in women and smokers, have also been reported (S. Chen et al. 2023; Shen et al. 2022). This subclass of flavonoids can be found in vegetables and fruits, such as asparagus, onions, lettuce, broccoli, tomatoes, apples, buckwheat leaves, sophora flowers, and leaves of particular eucalyptus species, accumulating mainly in the aerial parts of plants and playing a protective role against environmental stress (S. Chen et al. 2023; Ku et al. 2020; López Luengo 2002; Shen et al. 2022). Some of the most well‐known flavonols include quercetin, galangin, kaempferol, isorhamnetin, fisetin, rutin (flavonol glycoside), and myricetin (S. Chen et al. 2023; Kopustinskiene et al. 2020; López Luengo 2002; Shen et al. 2022).

### 3.3 Flavanones

Also known as dihydroflavones, these flavonoids lack a double bond between C2 and C3 in the C ring, distinguishing them from flavones. They can also serve as intermediates in the biosynthetic pathways of other flavonoids (Berga et al. 2023; S. Chen et al. 2023; Ku et al. 2020; Shen et al. 2022). They have intermediate structural entropy due to the lack of a double bond between carbons 2 and 3 in the C ring (Castellano et al. 2013). Flavanones show an anti‐inflammatory effect and moderate antioxidant capacity; however, in their prenylated form, they exhibit a high ability to induce vascular relaxation due to their enhanced liposolubility and their interaction with potassium and calcium channels in smooth muscle cells (Cos et al. 1998; Dahiya et al. 2023; X. Dong et al. 2008).

They are found mainly in citrus fruits, including oranges, lemons, tangerines, grapefruits, clementines, and limes (S. Chen et al. 2023; Kopustinskiene et al. 2020; Shen et al. 2022). Flavanones can contain hydroxyl groups at positions 5 and 7 of the A ring and may have hydroxyl and/or methoxy substituents at positions C3 or C4 of the B ring (S. Chen et al. 2023). The main representatives of flavanones are naringin, naringenin, hesperidin, hesperetin, pinocembrin, licorice, and eriodictyol (S. Chen et al. 2023; Kopustinskiene et al. 2020; Shen et al. 2022). Their effects can range from flavor enhancement, as seen with naringin in citrus juice and peel, to the restoration of altered thyroid function in rats, as demonstrated by naringenin and hesperetin (S. Chen et al. 2023; Shen et al. 2022).

### 3.4 Flavanols

Also known as catechins or flavan‐3‐ols, they are characterized by having two chiral centers in the molecule and a hydroxyl group at position 3 of the C ring. Like flavanones, they lack the double bond between carbons C2 and C3 and do not have the ketone group in the C ring (S. Chen et al. 2023; Ku et al. 2020; Shen et al. 2022). These compounds, commonly found in their non‐glycosylated form, are moderately soluble in water and show a very potent antioxidant effect. However, they have shown lower activity as xanthine oxidase inhibitors (Berga et al. 2023; Cos et al. 1998). This class of flavonoids exhibits low structural entropy due to the absence of the ketone group and the flexibility of their structure, which limits the diversity of their substitutions (Castellano et al. 2013).

They are predominantly found in fruits, such as apples, grapes, cherries, plums, apricots, berries, bananas, pears, and blueberries (S. Chen et al. 2023; Shen et al. 2022). Some examples include catechin, epicatechin, gallocatechin, epicatechin gallate, catechin gallate, and gallocatechin gallate. Daily intake of flavanols can provide benefits, including improving endothelial function, protecting blood vessels against tobacco damage, and preventing the development of cardiovascular diseases (S. Chen et al. 2023; Shen et al. 2022). When prenylated flavonoid derivatives are synthesized, an increased ability to inhibit lipid peroxidation is observed, enhancing the flavonoid vasodilatory effects (X. Dong et al. 2008).

### 3.5 Flavanonols

Flavanonols, or “dihydroflavonols,” differ from flavanols in that they possess a ketone group in the C ring, with taxifolin being the primary example. As intermediate compounds between flavonols and flavanones, they share physicochemical properties with both groups (Berga et al. 2023). The presence of hydroxyl and ketone groups in the C ring introduces structural variations, increasing entropy compared to flavanones, but to a lesser extent than flavonols (Castellano et al. 2013). They have potent antioxidants, antibacterial, anti‐inflammatory, hepatoprotective, anti‐angiogenic, anti‐Alzheimer, and anticancer effects, and they also exhibit intermediate efficacy as inhibitors of xanthine oxidase (Cos et al. 1998; Dahiya et al. 2023; Yuan et al. 2024).

Their distribution in nature is limited. They are commonly found in citrus fruits, red onions, and plants of the genus *Glycosmis* L and *Pinaceae* (Berga et al. 2023; Yuan et al. 2024). Flavanonols are metabolites derived from flavonols and leucoanthocyanidins, as well as key precursors in the biosynthetic pathway of flavonoids, specifically in the production of anthocyanins and proanthocyanidins (Lim et al. 2016). The most representative flavonol is taxifolin or dihydroquercetin, known for having a wide range of beneficial health properties, including the ability to improve capillary microcirculation and blood flow in the eye, as well as prevent damage to vascular structures in people with diabetes (Ku et al. 2020; Yuan et al. 2024).

### 3.6 Isoflavonoids

Isoflavones or isoflavonoids are characterized by having a B ring at the C3 position of the heterocyclic C ring of 4H‐chromen‐4‐one (S. Chen et al. 2023; Shen et al. 2022). To facilitate their storage in vacuoles, isoflavones are commonly modified by *O*‐glucosylation to form β‐glucosides and 6‐*O*‐malonylglucosides (Ku et al. 2020). They are well known for their estrogenic activity, antioxidant properties, and role in bone and cardiovascular health (Berga et al. 2023; Cos et al. 1998; Dahiya et al. 2023). Compared to other subclasses of flavonoids, their lower entropy has been related to the conformational characteristic of the skeleton of these flavonoids (Castellano et al. 2013). As for other subclasses of flavonoids, prenylation during synthesis increases lipophilicity and the strong affinity of isoflavones to biological membranes, thereby enhancing their interaction with proteins involved in vascular relaxation (X. Dong et al. 2008).

Isoflavones are found in legumes, such as soybeans, alfalfa, and chickpeas, and in small amounts in fruits, vegetables, nuts, and cereals. They compose the plant defense system against microbial infection, herbivory, wounds, and abiotic stress (Ku et al. 2020). The most well‐known isoflavonoids are genistein, daidzein, and glycitein (S. Chen et al. 2023; Shen et al. 2022). Isoflavones can exhibit various biological effects, including antiviral, anticancer, bactericidal, and antifungal properties. Their antioxidant properties can inhibit DNA damage induced by free radicals. They also act as phytoestrogens due to their structural similarities to estradiol‐17β (S. Chen et al. 2023; López Luengo 2002; Shen et al. 2022).

### 3.7 Anthocyanins

Anthocyanins are flavonoids characterized by having the basic structure of the chromophore 2‐phenyl‐benzopyrylium‐flavilium ion. They are the glycosylated form of anthocyanidins and are frequently found in fresh plants (Kopustinskiene et al. 2020; Ku et al. 2020). Additionally, the C3 position and the carbon atoms 3, 4, and 5 of ring B can be substituted by hydroxyl and/or methoxy groups (Kopustinskiene et al. 2020). They are water‐soluble compounds that are very sensitive to changes in pH, light, and temperature, exhibiting antioxidant and anti‐inflammatory properties but with low inhibitory activity against xanthine oxidase (Berga et al. 2023; Cos et al. 1998; Dahiya et al. 2023).

They are typically found in the outer cellular layers of various fruits and vegetables, as black currants, blueberries, red cabbage, tomatoes, purple sweet potatoes, eggplants, grapes, and berries (S. Chen et al. 2023; Shen et al. 2022). The most abundant and well‐known anthocyanins are petunidin, delphinidin, cyanidin, peonidin, malvidin, and pelargonidin (Kopustinskiene et al. 2020; Shen et al. 2022). Anthocyanins can be rapidly absorbed in the stomach and intestine and may even be found intact in the gastrointestinal wall. After extensive first‐pass metabolism, they enter systemic circulation as metabolites (Fang 2014a).

These compounds are considered soluble vacuolar pigments with a wide range of colors that vary with environmental pH, making them useful in culinary applications. Anthocyanins play a significant role in visual acuity, cholesterol metabolism, and cardiovascular disease risk reduction in humans. Additionally, they exhibit anti‐inflammatory and anti‐platelet aggregation activities and have very low toxicity (S. Chen et al. 2023; López Luengo 2002; Shen et al. 2022). Particularly, the antioxidant activity of this class of flavonoids depends on both the glycosylation of the ring B and the number and position of free hydroxyl groups around the pyrone ring (S. Chen et al. 2023; Shen et al. 2022).

### 3.8 Chalcones

Chalcones (1,3‐diaryl‐2‐propen‐1‐ones) are flavonoids whose main distinction is their open‐chain structure or “absence of C ring,” which can be understood as the presence of three prenyl groups (C5, C10, and C15) that may or may not be modified, connecting rings A and B (S. Chen et al. 2023; Shen et al. 2022). They are soluble in water and exhibit remarkable anti‐inflammatory and anticancer properties, but moderate antioxidant activity and very low inhibitory activity against xanthine oxidase (Berga et al. 2023; Cos et al. 1998; Dahiya et al. 2023). Chalcones are key intermediates for the biosynthesis of other flavonoid subclasses. They originate from the condensation of one p‐coumaroyl‐CoA molecule with three malonyl‐CoA molecules (Chavda et al. 2024; Dahiya et al. 2023). Due to their open structure (lacking the typical closed C ring of flavonoids), they are more flexible and are prone to isomerization; consequently, these compounds exhibit high molecular entropy (Castellano et al. 2013). The prenylation of chalcones can enhance their ability to interact with cell membranes (Dong et al. 2008).

Chalcones are present in the Fabaceae, Moraceae, Zingiberaceae, and Cannabaceae families (Shen et al. 2022). Like other flavonoid subclasses, these compounds exhibit various biological effects, including antioxidant, antibacterial, antihelminthic, antiulcer, antiviral, antiprotozoal, and anticancer properties (S. Chen et al. 2023; Shen et al. 2022). Furthermore, their structural characteristics and remarkable bioactivities have inspired the synthesis of chalcone analogs and the structural modification of natural chalcones. The most well‐known examples of representative derivatives with significant biological and pharmacological activity are xanthohumol and isobavachalcone (S. Chen et al. 2023; Shen et al. 2022).

Table 1 summarizes the general physicochemical characteristics of the different flavonoid subclasses, which are helpful for developing delivery systems.

**TABLE 1:** Flavonoid aglycone classes and their physicochemical properties.

Classification of flavonoids	Main enzymes in biosynthesis	Melting point	Molecular weight (Da)	LogP	References
Flavones	CHS and FNS	> 250°C	240–360	1.0–4.0	(Berga et al. 2023; Dixon and Steele 1999; S. Han et al. 2015; Rothwell et al. 2005)
Flavonols	FLS	> 250°C	250–390	0.5–3.5	(Berga et al. 2023; Dixon and Steele 1999; S. Han et al. 2015; Pourhajibagher and Bahador 2024; Rothwell et al. 2005)
Flavanones	CHS, CHI and F3H	227°C–251°C	260–380	1.5–4.5	(Berga et al. 2023; Echeverría et al. 2017; S. Han et al. 2015; Souza et al. 2020)
Flavanols	ANR	141°C–220°C	270–420	0.2–3.0	(Berga et al. 2023; Bordenave et al. 2014; Gymnastiar et al. 2024; Lewis et al. 2023)
Flavanonols	F3βH	> 300°C	280–400	1.0–4.0	(Berga et al. 2023; Hussain et al. 2024; Y. Liu et al. 2023)
Isoflavonoids	IFS	> 250°C	250–420	1.5–5.0	(Berga et al. 2023; Dixon and Steele 1999; Rothwell et al. 2005; Stefaniu and Pirvu 2022)
Anthocyanins	DFR and ANS	> 300°C (variable)	240–500	−2.0–2.0	(Berga et al. 2023; S. Han et al. 2015; Lewis et al. 2023)
Chalcones	CHS	> 300°C	220–450	2.0–6.0	(Berga et al. 2023; Dixon and Steele 1999; Erik et al. 2025; S. Han et al. 2015; Okolo et al. 2021)

Abbreviations: ANR, anthocyanidin reductase; ANS, anthocyanidin synthase; CHI, chalcone isomerase; CHS, chalcone synthase; DFR, dihydroflavonol 4‐reductase; F3βH, flavanone 3β hydroxylase; F3H, flavanone 3‐hydroxylase; FLS, flavonol synthase; FNS, flavone synthase; IFS, isoflavone synthase.

Glycosylated flavonoids are more soluble and stable than aglycones. This characteristic is relevant for administration routes with significant variations, such as the oral route. Studies have shown that glycosides can remain intact during transport via the sodium‐dependent glucose transporter 1 (SGLT1) (Walle 2004). However, most of them follow another mechanism in which the first step occurs in the bacterial flora of the colon, where uncontrolled hydrolysis of these glycolylated flavonoids takes place. This step can be the rate‐limiting step in the absorption ofany flavonoids (Z. Chen et al. 2014; Walle 2004; Z. Chen et al. 2014).

On the other hand, it has also been observed that (Berrin et al. 2002) glycoside hydrolysis can occur due to the presence of enzymes such as β‐glucosidase (βG) in human tissues, including the liver, small intestine, spleen, and kidney (Berrin et al. 2002), as well as glucocerebrosidase and phlorizin hydrolase (LPH). These enzymes facilitate the release of aglycones, allowing them to enter intestinal epithelial cells (enterocytes) and reach the systemic circulation. Notably, LPH is an essential hydrolase in the intestinal lumen, as it is a membrane protein that breaks down flavonoids without transporting them into cells (Berrin et al. 2002; Z. Chen et al. 2014; Dahiya et al. 2023; Walle 2004).

In the nasal cavity, various hydrolase enzymes have been identified, such as peptidases, epoxide hydrolases, and carboxylesterases, which are involved in the hydrolytic enzymatic activity on a wide variety of xenobiotics entering through the nasal tract (Dahl and Hadley 1991; Wüthrich et al. 1994). Considering the organs in which the different hydrolases are expressed, the metabolism mediated by these enzymes is relevant in various routes of administration, including sublingual, rectal, intragastric, intraperitoneal, intravenous, oral, and even intranasal. To our knowledge, studies directly addressing the enzymatic hydrolysis of flavonoids in the nasal cavity have not been reported to date.

Flavonoids are compounds with potent antioxidant properties and significant entropic variations. Their aglycones typically exhibit low solubility. This characteristic has been taken into account in the formulations for several administration routes, such as ophthalmic, intrathecal, intranasal, cutaneous, pulmonary, intramuscular, and subcutaneous, where different strategies to improve apparent solubility have been reported. Table 2 shows the most commonly used administration routes across the different flavonoid classes, specifically those used in investigations where flavonoids were administered without prior formulation development.

**TABLE 2:** Studies of flavonoids and the corresponding administration routes.

Classification of flavonoids	Administration route	Compound	References
Flavones	Oral	Apigenin (4′,5,7‐trihydroxyflavone) Baicalein Baicalin Chrysin (5,7‐dihydroxyflavone) Scutellarin Icariin Linarin Luteolin (3′,4′,5,7‐tetrahydroxyflavone) Luteolin 7‐O‐glucoside Luteolin 7‐O‐glucuronide Oroxylin A Sudachitin Tangeretin Tricin (4′,5,7‐trihydroxy‐3′,5′‐dimethoxyflavone) Vitexin Wogonin	(Cai et al. 2007; Z. Chen et al. 2012; Lai et al. 2010; Li, Zhang, et al. 2011; C. R. Li et al. 2012; Gradolatto et al. 2005; Walle 2007; X. Chen et al. 2006; S. Xu et al. 2017; Y. Li et al. 2019; H. Liu et al. 2022; Sarawek et al. 2008; Hatanaka et al. 2024; Nielsen et al. 2000; P. Dong et al. 2021)
	Intragastric	Linarin	(Y. Li et al. 2019)
Flavonols	Oral	3′, 4′‐dimethoxy flavonol‐3‐β‐d‐glucopyranoside Kaempferol Gossypetin Hibifolin (gossypetin‐8‐O‐β‐d‐glucuropyranoside) Hyperoside (quercetin‐3‐O‐β‐d‐galactoside) Isorhamnetin Isoquercitrin (quercetin‐3‐O‐β‐d‐glucoside) Myricetin Myricitrin Quercetin Quercetin‐3′‐O‐glucoside (quercetin‐3′‐O‐β‐d‐glucoside) Rutin	(Lou et al. 2018; Y. Zhu et al. 2016; Alkhalidy et al. 2018; H. Liu et al. 2022; Vissiennon et al. 2012; Guo et al. 2010; Xue et al. 2011; Dayem et al. 2015; Maronpot et al. 2015; X. Chen et al. 2005; Maciej et al. 2015; Nguyen et al. 2015; Young and Morris 2007; Abdel‐Ghaffar et al. 2018)
	Intravenous	3′, 4′‐dimethoxy flavonol‐3‐β‐d‐glucopyranoside Fisetin (3,3′,4′,7‐Tetrahydroxyflavone) Quercetin Rutin	(Lou et al. 2018; Shia et al. 2009; Chen et al. 2005; Demrow et al. 1995; Young and Morris 2007)
	Intragastric	3′,4′,5′‐trimethoxyflavonol Isoquercitrin (quercetin‐3‐O‐β‐d‐glucoside) Quercetin Rutin	(Saad et al. 2012; Crespy et al. 2002; Demrow et al. 1995)
	Intraperitoneal	Fisetin (3,3′,4′,7‐Tetrahydroxyflavone) Kaempferol Myricetin Quercetin Rutin	(Krasieva et al. 2015; Vissiennon et al. 2012; Bariexca et al. 2019; Guardia et al. 2001; Moon et al. 2008)
	Intraduodenal	Quercetin Rutin	(Gohlke, Ingelmann, Nürnberg, Starke, et al. 2013; Gohlke, Ingelmann, Nürnberg, Weitzel, et al. 2013; Murota et al. 2013)
Flavanones	Oral	5,7,3′,5′‐tetrahydroxyflavanone 6‐prenylnaringenin 8‐prenylnaringenin Eriocitrin Eriodyctiol Hesperetin Hesperidin Neo hesperidin Naringenin Naringin Pinocembrin	(Gaggeri et al. 2012; van Breemen et al. 2014; Manthey et al. 2022; Kanaze et al. 2007; Jin et al. 2010; Nectoux et al. 2019; Wasowski et al. 2012; Z. Xu et al. 2019; M. Liu et al. 2011, 2012; Zeng et al. 2020; H. Liu et al. 2022)
	Intraperitoneal	Hesperetin Hesperidin	(Bariexca et al. 2019; Guardia et al. 2001; Wasowski et al. 2012)
Flavanols	Intraperitoneal	(+)‐Catequina (−)‐Epigallocatechin‐3‐gallate	(Bariexca et al. 2019; Moon et al. 2008)
	Oral	[3‐^3^H]‐Catechin Epicatechin (−)‐Epicatechin‐3′‐β‐d‐glucuronide (−)‐Epicatechin 3′‐sulfate 3′‐O‐methyl‐(−)‐epicatechin 4′‐sulfate 3‐[3H]‐(2R,3R)‐epicatechin [3‐3H]‐epicatechin (−)‐Epigallocatechin‐3‐gallate	(Catterall et al. 2003; Gutiérrez‐Salmeán et al. 2014; Loke et al. 2008; Urpi‐Sarda et al. 2009; Actis‐Goretta et al. 2012; Abrahamse et al. 2005; Young and Morris 2007)
	Intravenous	[3‐3H]‐Catechin [3‐3H]‐epicatechin (−)‐Epigallocatechin‐3‐gallate	(Catterall et al. 2003; Young and Morris 2007)
Flavanonols	Oral	Aromadendrin Taxifolin (dihydroquercetin)	(Gaggeri et al. 2012; Li, Su, et al. 2023; Ullah and Khan 2018; C. Wu et al. 2017)
	Intraperitoneal	Taxifolin (dihydroquercetin)	(X. Chen et al. 2018)
	Intragastric	Taxifolin (dihydroquercetin)	(Li, Peng, et al. 2023; H. Su et al. 2020)
Isoflavonoids	Oral	Biochanin A Daidzein Formononetin Genistein Ipriflavone Kakkalide Methoxyisoflavone Ononin Puerarin	(Moon et al. 2006; Singh et al. 2010, 2012; S. Zhang et al. 2010; Jeong Choi 2006; L. Y. Luo et al. 2018; Z. Yang et al. 2010; S. Zhou et al. 2008; Iannone et al. 2019, 2021; X. Bai et al. 2011; H. Liu et al. 2022; H. Zhu et al. 2021)
	Intravenous	Biochanin A Daidzin Genistein Puerarin	(Moon et al. 2006; Young and Morris 2007; Tian et al. 2002; Z. Yang et al. 2010; C. F. Luo et al. 2010)
	Intraperitoneal	Biochanin A	(Moon et al. 2006, 2008)
	Intragastric	Daidzein Genistein	(Álvarez et al. 2011)
	Subcutaneous	Daidzein Genistein	(Sosic‐Jurjevic et al. 2007)
Anthocyanins	Intravenous	Cyanidin 3‐glucoside	(Marczylo et al. 2009; Vanzo et al. 2013)
	Oral	Cyanidin 3‐glucoside Cyanidin‐3‐galactoside Cyanidin‐3‐rutinoside Cyanidin‐3‐O‐sambubioside Cyanidin (anthocyanidin) Delphinidin‐3‐glucoside Delphinidin 3‐O‐β‐D‐glucoside Delphinidin 3‐O‐β‐d‐glucopyranoside Delphinidin‐3‐O‐sambubioside Delphinidin (anthocyanidin) Malvidin‐3‐glucoside Malvidin‐3‐galactoside Malvidin (anthocyanidin) Pelargonidin‐3‐glucoside Pelargonidin (anthocyanidin) Petunidin (anthocyanidin) Peonidin (anthocyanidin)	(Fang 2014b; Hassimotto et al. 2008; Hiroyuki et al. 2009; Marczylo et al. 2009; Majdoub et al. 2019; Talavéra et al. 2006; Ichiyanagi et al. 2004a; Ichiyanagi et al. 2004b; Khoo et al. 2017)
	Intragastric	Malvidin‐3‐glucoside	(Passamonti et al. 2005)
Chalcones	Oral	Isoliquiritigenin Isoliquiritin Naringenin Chalcone Xanthohumol Isobavachalcone Phloridzin	(J. Wu et al. 2021; Yoshimura et al. 2009; H. H. Bai et al. 2022; van Breemen et al. 2014; S. Su et al. 2015; Najafian et al. 2012)
	Intraperitoneal	Butein	(Wang et al. 2017)

In this context, the intranasal route has attracted increasing attention as a promising alternative for the administration of flavonoids, particularly to the central nervous system. Consequently, intranasal delivery systems are of great interest, and formulation strategies have been developed to overcome solubility limitations, increase nasal residence time, and optimize transport to the brain.

## 4 Drug Delivery Systems for the Intranasal Administration of Flavonoids

Several strategies have been developed to overcome the low solubility of flavonoids for intranasal administration, ranging from polymer‐based particulate systems at the micrometer scale, such as microspheres, to nanometer‐scale systems, including nanoparticles. Similarly, oil‐in‐water emulsions formulated with one or more emulsifying agents and/or surfactants have been developed and are classified for nasal application according to their droplet size (Boyuklieva and Pilicheva 2022; Ranjbar et al. 2023).

Liposomes have also been used to generate vesicles that encapsulate drugs, there by facilitating their delivery to the brain (Mainardes et al. 2006; Qiu et al. 2025; Ranjbar et al. 2023). Other alternatives used in nasal formulations include complexation with excipients such as cyclodextrins, which enhance the solubility and stability of flavonoids in the nasal mucosa. Additionally, in situ gelling systems have been employed to reduce the formulation clearance. These systems involve a polymer solution that undergoes a phase transition in response to changes in properties such as temperature or pH (Qian et al. 2025; Qiu et al. 2025; Rassu et al. 2021; Costa et al. 2021). The diverse nasal delivery systems have been applied to the flavonoid aglycones and exhibit distinct advantages and limitations, as summarized in Table 3.

**TABLE 3:** Advantages and limitations of drug delivery systems for intranasal administration of flavonoid aglycones.

Type of carrier	Advantages	Limitations	Applications	References
Nanoparticles	Protect encapsulated drugs from degradation. High stability. Enhance nasal permeation. May exhibit mucoadhesive properties. Enable sustained release and direct transport to the brain.	More complex manufacturing processes. Potential variability in encapsulation efficiency. Higher cost.	Poorly soluble flavonoids. Example: quercetin	(Desai 2012; Huang et al. 2023; Mu et al. 2024; Qiu et al. 2025)
Nanoemulsions	Increase the apparent aqueous solubility. Improve absorption. Can incorporate mucoadhesive agents. Easy to scale up industrially.	Less stable than solid systems. Require strict control of droplet size and stability.	Highly hydrophobic flavonoids. Example: kaempferol	(Choi and McClements 2020; Colombo et al. 2018; Preeti et al. 2023; Tartari et al. 2025)
Cyclodextrin complexes	Drastically improve apparent aqueous solubility. Reduce mucosal irritation. Enhance stability against oxidation. Compatible with dry powders and nasal sprays.	Limited drug‐loading capacity. Not all cyclodextrins are safe for intranasal use (HPβCD or SBE‐βCD recommended).	Flavonoids with low solubility. Example: quercetin	(Merkus et al. 1999; Rassu et al. 2021; Saitani et al. 2025; Spiridon and Anghel 2025; Văruț et al. 2025)
Liposomes	Biocompatible Allow high loading of lipophilic compounds. Provide protection and moderate sustained release. Enhance absorption.	Low stability. Require refrigeration. Shorter shelf life.	Flavonoids sensitive to oxidation or degradation. Example: 7,8‐dihydroxyflavone (7,8‐DHF)	(Ahmed et al. 2024; Duong et al. 2023; Khute and Jangde 2023)
In situ gels	Prolong residence time on the nasal mucosa. Enable sustained release. Improve bioavailability. Reduce dosing frequency.	Stability issues and susceptibility to degradation in solution. Difficult to formulate with highly hydrophobic molecules without an additional carrier.	Flavonoids requiring prolonged release. Example: dihydroquercetin	(Abou‐Taleb et al. 2024; Çelik et al. 2023; Galgatte et al. 2014)
Microemulsions	Enhance nasal permeation Thermodynamically stable (do not require high energy or ultrasonication). Low viscosity.	Do not provide sustained release due to rapid clearance. May require high surfactant concentrations to maintain stability.	Extremely hydrophobic flavonoids. Example: fisetin	(Froelich et al. 2021; Tamizmaran et al. 2025)
Microparticles	High stability. Sustained release. Facilitate nasal dry powder formulations. May exhibit mucoadhesive properties.	High particle size may reduce permeation. Spray‐drying or lyophilization processes increase formulation complexity.	Flavonoids sensitive to aqueous degradation. Example: quercetin	(Gavini et al. 2005; Henriques et al. 2022; Papakyriakopoulou et al. 2021)

In general, the high permeability of the nasal epithelium renders it particularly susceptible to adverse effects from drugs and excipients, regardless of the type of nasal delivery system used. This susceptibility arises because the presence of any foreign material within the nasal cavity may induce irritation of epithelial cells and local nerve endings. Moreover, certain compounds may trigger adverse reactions (Alghareeb et al. 2024; Pesic et al. 2020). Additionally, the presence of preservatives in nasal formulations has been shown to be potentially detrimental to the nasal cavity, as these compounds may disrupt mucociliary clearance, in some cases irreversibly, and induce morphological changes in the nasal respiratory mucosa (Mallants et al. 2007; Tratnjek et al. 2021).

These points emphasize the need to carefully evaluate and select appropriate components when developing delivery systems to ensure safe, efficient drug delivery from the nasal cavity.

## 5 Regulatory Considerations for the Use of Flavonoids in Drugs and Foods

Currently, the Food and Drug Administration (FDA) has not established specific regulatory guidance for the use of flavonoids. Nevertheless, regulatory frameworks applicable to botanical or natural products are available, under which these polyphenolic compounds are classified. From a regulatory perspective, a critical aspect is the appropriate classification of the product according to its intended use, whether as a pharmaceutical drug or as a dietary supplement, as each category is subject to different regulatory requirements, evaluation pathways, and levels of oversight (Food and Drug Administration 2016). Although flavonoids are now regulated as supplements or food ingredients, any intranasal therapeutic formulation would be classified by the FDA as a pharmaceutical drug and subject to drug regulations.

In this context, when flavonoids are developed as part of a pharmaceutical drug, the FDA requires compliance with regulatory standards comparable to those applied to conventional drugs. These requirements include establishing quality control measures using chromatographic profiles and chemical markers, generating preclinical and clinical data, demonstrating batch‐to‐batch consistency, and submitting Investigational New Drug (IND) and New Drug Application (NDA) applications (Food and Drug Administration 2016).

Likewise, flavonoids used as ingredients in foods or dietary supplements can follow the Generally Recognized as Safe (GRAS) regulatory process under CFR 170.30, provided their safety is demonstrated under the proposed conditions of use. To support a GRAS determination, the FDA requires a detailed characterization of the ingredient and its manufacturing process, an estimate of dietary intake, and scientific evidence of safety based on toxicological studies and/or a documented history of safe consumption, supported by evaluation from qualified experts. Although GRAS notification is voluntary, the GRAS Notification program allows the FDA to review this information and issue a “no objection” letter when the evidence is deemed sufficient (Generally Recognized as Safe 2023; Subpart B—Food Additive Safety 2004).

## 6 Regulatory Considerations for Nasal Administration

The development of flavonoid‐based intranasal drugs requires compliance with strict regulatory frameworks, primarily established by the Food and Drug Administration (FDA) and the European Medicines Agency (EMA) to ensure product quality, safety, and efficacy.

FDA states that intranasally administered products are regulated as drugs. The manufacturing and commercialization of any intranasal product intended for therapeutic use must comply with Current Good Manufacturing Practices (cGMP) for pharmaceutical products, as described in 21 CFR Parts 210 and 211. These regulations encompass quality control, the implementation of standard operating procedures, deviation management, and control of manufacturing processes to ensure product safety, quality, and efficacy (Food and Drug Administration 2002; 2024).

Additionally, for intranasally administered products in the form of aerosols or sprays, the FDA has issued specific guidance documents recommending conducting bioequivalence studies and a comprehensive evaluation of both device and formulation performance. These assessments aim to demonstrate therapeutic equivalence or adequate and consistent product performance (Food and Drug Administration 2003).

Similarly, the European Medicines Agency (EMA) requires that intranasally administered medicinal products demonstrate both local and systemic safety, adequate bioavailability, and consistency in manufacturing processes, in accordance with the pharmaceutical quality guidelines harmonized by the International Council for Harmonization (ICH). These include ICH Q8 (Pharmaceutical Development), ICH Q9 (Quality Risk Management), and ICH Q10 (Pharmaceutical Quality System), which establish the principles for product design, control, and quality assurance throughout the entire product lifecycle (European Medicines Agency 2025).

In addition, the European Medicines Agency (EMA) has issued a specific guideline on the quality of inhaled and nasal products, establishing detailed regulatory criteria for medicinal products intended for delivery to the nasal mucosa or the lungs. Regulated aspects include the definition and specification of active substances and excipients, along with their appropriate physicochemical characterization. Furthermore, the evaluation of critical properties, such as particle size, particle size distribution, and particle morphology (when applicable) is required, as these attributes are essential to ensure therapeutic consistency and the safety of the final product (European Medicines Agency 2025).

Within this regulatory framework, flavonoids have progressed beyond their extensive use in preclinical studies and academic research and have also been successfully translated into commercial applications. In line with the regulations and guidelines established by the Food and Drug Administration (FDA), which recognize the safety of several flavonoids for human consumption, many FDA‐approved products containing flavonoids are currently available on the market. These products are summarized in Table 4.

**TABLE 4:** FDA‐recognized flavonoid‐containing products (GRAS status).

Product	Flavonoids	FDA approvals
Bonolive	Luteolin‐7‐*O*‐glucoside, apigenin‐7‐*O*‐glucoside, diosmetin‐7‐glucoside, luteolin, diosmetin, rutin, catechin and quercetin	GRAS Notice 1119
Ocean Spray Cranberries	Cyanidin 3‐*O*‐glucoside, quercetin, quercitrin, hyperoside, and myricetin.	GRAS Notice 873
Reducose 5%	Kaempferol, quercetin	GRAS Notice 894
AppleActiv Dapp/Leahy Dapp (Apple peel powder)	Quercetin	GRAS Notice 613
Blue California	Dihydroquercetin or taxifolin	GRAS Notice 916 with Amendments GRAS Notice 826 GRAS Letter 916
RUNA Concentrate	Quercetin‐3‐*O* hexose	GRAS Notice 883
Gyusa.g	Quercetin‐3‐*O* hexose	GRAS Notice 869
AmaTea	Quercetin‐3‐*O* hexose	GRAS Notice 835
AppleActiv DAPP/Leahy DAPP (Dried Apple Peel Powder)	Quercetin	GRAS Notice 805
Quercegen Pharma LLC	Quercetin	GRAS Notice 341
Orange Extract	Hesperidin, hesperetin, narirutin, naringenin, eriodictyol	GRAS Notice 796
Orange pomace	Hesperidin, narirutin, didymin	GRAS Notice 719
Hayashibara	Hesperetin, hesperidin	GRAS Letter 901 GRAS Notice 901
Kemin	(−)‐epicatechin, (−)‐epigallocatechin, (−)‐epicatechin gallate, and (−)‐epigallocatechin gallate, (+)‐catechin, (+)‐catechin gallate, (+)‐gallocatechin and (+)‐gallocatechin gallate	GRAS Notice 772

*Note:* Approved products or ingredients that have GRAS status found in FDA databases as food supplements on the website: www.fda.gov (09/11/2024).

## 7 Pharmacological Properties of Flavonoids in the Nasal Cavity

Flavonoids can provide various health benefits, including protecting the nasal environment or reducing the symptoms provoked by external aggressors. These benefits primarily come from the flavonoids found in nutritional sources (M. Zhang et al. 2023).

The pharmaceutical use of flavonoids has mainly been in herbal medicines containing them. However, understanding the biological mechanisms of action of isolated flavonoids and their pharmacological activities has made this class of secondary metabolites a target for drug development (Panche et al. 2016).

This section focuses on the beneficial effect, pharmacological activities, and biochemical effects of isolated flavonoids in the nasal cavity. In general, flavonoids can be quite versatile, as seen with compounds like naringin and quercetin, which can significantly enhance immune system function and act as protectors against damage to various internal organs or tissues caused by oxidation (Shen et al. 2022), or compounds like morin, which inhibit the hyperphosphorylation of tau protein in human neuroblastoma cells, an important mechanism for preventing the development of neurodegenerative diseases such as Alzheimer's (Gong et al. 2011). Table 5 shows studies on pharmacological activities and biochemical effects of different classes of flavonoids.

**TABLE 5:** Pharmacological activities and experimental models of flavonoids and their derivatives on the nasal cavity.

Classification	Flavonoid	Pharmacological properties	Main model or method of study	References
Flavones	Baicalein	Promoter and protector of the nasal microbiota	Multi‐omics pilot analysis on indoor, oral, and both nasal cavity of dust samples	Zhang et al. 2023
	Baicalin	Anti‐allergic activity	In vitro: histamine quantification and analysis of JAK2, p‐JAK2, STAT5, p‐STAT5, IKKβ, p‐IKKβ, IκBα, p‐IκBα and NF‐κB (p65) by WB in human mast cells In vivo: evaluation of symptoms and histological changes related to allergic rhinitis in the nasal mucosa from guinea pigs	Zhou et al. 2016
		Anti‐inflammatory activity	In vitro: evaluation of the effects of baicalin on type 2 immune responses in human airway epithelial cells and mast cells	Yoshida et al. 2021
	Eupatiline	Promoter and protector of the nasal microbiota	Multi‐omics pilot analysis on indoor, oral, and both nasal cavity of dust samples	Zhang et al. 2023
	Tangeritin	Promoter and protector of the nasal microbiota	Multi‐omics pilot analysis on indoor, oral, and both nasal cavity of dust samples	Zhang et al. 2023
	Luteoline	Anti‐allergic activity	In vitro: patch and voltage fixation in Ussing chambers in human embryonic kidney cells transfected with human ANO1 expression vector hANO1‐HEK293T and human airway epithelial cells (Calu‐3) sensitized to IL‐4	Kim et al. 2020
	Luteolin‐7‐*O*‐glucoside	Anti‐allergic activity	In vivo: evaluation of nasal symptoms, antigen activation, and histamine release in ovalbumin‐sensitized rats (OVA)	Inoue et al. 2002
	Chrysin	Anti‐inflammatory activity	In vitro: measurement of ciliary beating frequency, PKC activation and immunofluorescence microscopy for T2R38 and T2R14 in human primary nasal cells	Hariri et al. 2017
	Apigenin	Anti‐allergic activity	In vivo: evaluation of nasal symptoms, serum IgE, IgG1, and β‐hexosaminidase quantification, and histamine release of OVA‐sensitized BALB/c mice	Chen et al. 2020
		Anti‐inflammatory activity	In vitro: cell viability and detection of apoptosis in HMC‐1 mast cells In vivo: biochemical measurement of β‐hexosaminidase, histamine, eosinophil cationic protein (ECP), OVA‐specific IgE, IgG1, and IgG2a in the serum of BALB/c mice OVA‐sensitized	Li, Zhang, and Zhao 2023
			Ex vivo: evaluation of fibronectin expression, type I collagen, and phosphorylation of MAPK in nasal fibroblasts and human lower nasal turbinate tissues	Yang et al. 2018
			In vitro: measurement of ciliary beating frequency, PKC activation and immunofluorescence microscopy for T2R38 and T2R14 in human primary nasal cells	Hariri et al. 2017
		Secretagogue and Mucoregulator Activity	In vitro: transepithelial measurements and patch fixation in human airway epithelial cells (Calu‐3) In vivo: evaluation of nasal potential difference in healthy humans	Illek and Fischer 1998
	5,7,4′‐trimetoxiflavone (TMF)	Secretagogue and Mucoregulator Activity	In vitro: voltage fixation in Ussing chambers in human airway epithelial cells (Calu‐3) In vivo: evaluation of nasal potential differential in humans.	Fischer and Illek 2008
	Isoorientin	Anti‐inflammatory activity	In vivo: Evaluation of nasal symptoms of allergic rhinitis, measurement of serum inflammatory markers like histamine, OVA‐specific IgE and cytokines, and analysis of p65, p‐IκBα, IκBα and β‐Actin expression by inmunoblot in nasal tissure from C57BL/6 mice C57BL/6 sensitized with OVA.	Huanga et al. 2024
	Wogonin	Anti‐inflammatory activity	In vitro: measurement of ciliary beating frequency, PKC activation and immunofluorescence microscopy for T2R38 and T2R14 in human primary nasal cells	Hariri et al. 2017
Flavonols	Quercetin	Anti‐ oxidant activity on the human nasal mucosa	Ex vivo: comet assay on human nasal mucosal cells from healthy patients.	Reiter et al. 2009
		Anti‐allergic activity	In vitro: evaluation of NO levels and transcription factor STAT6 and activation of iNOS mRNA expression in human nasal epithelial cells (HNEpCs)	Ebihara et al. 2018
		Anti‐inflammatory and anti‐allergic activity	In vivo: Analysis of neuropeptides substance P (SP), calcitonin gene‐related peptide (CGRP), and nerve growth factor (NGF) release and nasal allergy‐like symptoms assay in Sprague–Dawley rats sensitized to toluene 2,4‐diisocyanate (TDI)	Kashiwabara et al. 2016
		Anti‐allergic activity	In vitro: Analysis of H1R mRNA and GAPDH mRNA and protein expression by RT‐PCR and WB, respectively, in HeLa cells In vivo: evaluation of nasal allergy‐like symptoms in Brown Norway rats sensitized with (TDI).	Hattori et al. 2013
		Anti‐inflammatory activity	In vitro: evaluation of anti‐inflammatory activity in LPS‐ induced inflammation by determination of miR‐21, DMBT1, and NF‐κB mRNA expression in human nasal epithelial cells	Cheng et al. 2022
		CFTR modulating activity	In vitro: measurement of chloride ions current in Ussing chambers in Fisher rat thyroid cells and human bronchial epithelial cells CFBE41o−. In vivo: evaluation of nasal potential difference in mice and humans.	Pyle et al. 2010
			In vitro: ion transport in Ussing chambers in primary cultures of murine nasal septal epithelium (MNSE) and human sinonasal epithelium (HSNE)	Zhang et al. 2011
		Secretagogue and Mucoregulator Activity	In vitro: transepithelial measurements and patch fixation in human airway epithelial cells (Calu‐3) In vivo: evaluation of nasal potential difference in healthy humans	Illek and Fischer 1998
	Diosmetin	Anti‐inflammatory and anti‐allergic activities	In vivo: evaluation of nasal symptoms, ELISA for evaluated (IgE), IgG1 and levels of Th1‐ and Th2‐related cytokines and WB for determined NF‐κB and SIRT1/NF‐κB in serum and nasal mucosa of allergic rhinitis in OVA‐ sensitized XX mice, respectively	Hu and Peng 2023
			In vivo: evaluation of nasal symptoms, measurement of allergic markers PGD2 and LTC4 and proinflammatory cytokines interleukin and TNF‐alpha in OVA‐sensitized BalB/c mice.	Yu and Xu 2024
	3‐*O*‐ methylquercetin	Antirhinoviral activity	Ex vivo: ciliary beat frequency (CBF) measurement in nasal epithelial cells of surgical specimens from human nasal polyps.	Dimova et al. 2003
	Morin	Anti‐allergic activity	In vivo: Evaluation of nasal symptoms, GATA3, T‐bet, p‐STAT6, SOCS1, and β‐Actin by RT‐PCR and WB, in OVA‐sensitized BALB/c mice.	Liang et al. 2019
	Kaempferol	Secretagogue and Mucoregulator Activity	In vitro: transepithelial measurements and patch fixation in human airway epithelial cells (Calu‐3) In vivo: evaluation of nasal potential difference in healthy humans	Illek and Fischer 1998
		Protective activity on respiratory epithelial cells	In vitro: Phosphorylated NF‐κB and total NF‐κB level determination by WB, measurement of intracellular ROS in human epithelial cells BEAS‐2B	Podder et al. 2014
	Troxerutin	Relieve symptoms of the common cold	In vivo: evaluation of symptoms like nasal Obstruction, sore throat and rhinorrhea in volunteers with the common cold	Turner et al. 2004
Flavanones	Hesperidin	Promoter and protector of the nasal microbiota	Multi‐omics pilot analysis on indoor, oral, and both nasal cavities dust samples	Zhang et al. 2023
		CFTR activator	In vitro: voltage fixation in Ussing chambers in primary cultures of MNSE and HSNE In vivo: evaluation of the nasal potential differential in mice C57/BL6	Azbell et al. 2010
Flavanols	Epigallocatechin gallate	Anti‐inflammatory activity	In vitro: analysis of BECN1, SQSTM1 and CFTR protein expression by WB in CFBE41o − cells In vivo: evaluation of iodide efflux, expression of TNF and CXCL8 cytokines and spirometry tests and sweat chloride measurements in humans	De Stefano et al. 2014
Isoflavones	Genistein	Anti‐inflammatory activity	In vitro: Measurement of chloride ion current in HeLa cells using viral vectors carrying G551D‐CFTR In vivo: evaluation of nasal potential differential in humans	Illek et al. 1999
			Ex vivo: voltage fixation in Ussing chambers in nasal polyps and rectal mucosa of normal individuals	Mall et al. 2000
		Secretagogue and Mucoregulator Activity	In vitro: transepithelial measurements and patch fixation in human airway epithelial cells (Calu‐3) In vivo: evaluation of nasal potential difference in healthy humans	Illek and Fischer 1998
Chalcones	Isoliquiritigenin	Promoter and protector of the nasal microbiota	Multi‐omics pilot analysis on indoors, oral, and both nasal cavity dust samples	Zhang et al. 2023
	Hydroxysafflor yellow A (HYA)	Anti‐inflammatory and anti‐allergic activity	In vivo: evaluation of antioxidant parameters, specific immunoglobulins like IgE and histamine and cytokines like Interleukins and TNF‐α in OVA‐sensitized BALB/c mice	Ma et al. 2023

Abbreviations: ANO1, calcium‐activated chloride channel anoctamin‐1; CFTR, cystic fibrosis transmembrane conductance regulator.

## 8 Diseases That Affect the Nasal Cavity

A diverse microbial community comprising multiple bacterial phyla inhabits the nasal cavity. The stability of this microbiota is crucial for respiratory health. Disruptions in this microbiota, known as dysbiosis, can disrupt the integrity of the nasal barrier and increase the invasion of pathogenic bacteria, potentially leading to the development of both acute and chronic respiratory conditions, such as rhinosinusitis and asthma (Cho et al. 2020; de Steenhuijsen Piters et al. 2015; Lemon 2020).

Regarding nasal microbiota, exploratory studies have shown that the flavonoids baicalein, eupatilin, isoliquiritigenin, tangeritin, and hesperidin appear to promote greater abundance of protective microbiota, with a positive impact on nasal health. This potential to inhibit biofilm formation and the aggregation of pathogenic microorganisms is the leading hypothesis to explain this phenomenon. However, these mechanisms must be confirmed and explored in greater detail (M. Zhang et al. 2023).

Quercetin, one of the most studied flavonols, which generally plays a significant role in protecting cells from oxidative stress induced by ROS, can also exhibit this protective activity in human nasal mucosa at concentrations of 5 and 50 μM, as observed by Reiter and Cols (Reiter et al. 2009).

Another flavonol potentially protecting respiratory epithelial cells is kaempferol (Podder et al. 2014). Kaempferol suppressed the overexpression of the mucin gene MUC5AC through the nuclear factor kappa B (NF‐κB) pathway in BEAS‐2B cells from the human bronchial epithelium exposed to paraquat. Paraquat, a common herbicide associated with lung damage, is a potent producer of ROS. This suggests that the flavonoid may help prevent chronic cough and excessive sputum production, which are caused by the accumulation of MUC5AC in the respiratory tract. This gene is distinctive and phenotypic in asthma, cystic fibrosis, nasal allergy, rhinitis, and sinusitis (Podder et al. 2014).

The above highlights the advantages of the nose as an accessible route for generating beneficial local effects, enhancing the protection of this organ against various environmental factors (Cingi et al. 2021). Furthermore, nasal administration has been widely used for the local treatment of conditions that specifically affect the nose and paranasal sinuses (Cingi et al. 2021; Grassin‐Delyle et al. 2012). In this context, flavonoids stand out for their key role in the treatment of respiratory conditions at the local level, thanks to their anti‐inflammatory, antioxidant, and protective properties, which directly contribute to improving upper respiratory tract health.

### 8.1 Common Cold

A globally recognized illness is the “common cold,” one of the leading causes of acute morbidity in the general population. The rhinovirus is the primary cause. Nasal symptoms are often predominant because the upper respiratory tract is primarily affected. Since there are no authorized compounds against the rhinovirus (Casanova et al. 2018), studying drugs with potential therapeutic benefits is of great importance. An example is 3‐O‐methylquercetin (3OMQ), a flavonoid that has shown significant anti‐picornaviral activity and potential anti‐inflammatory and antioxidant properties. The antiviral mechanism of 3OMQ has been linked to inhibition of an early step in viral replication, thereby reducing viral RNA and protein synthesis. Part of this action has been attributed to the methoxy and hydroxyl groups at the C3 and C5 positions of the flavone skeleton (Dimova et al. 2003).

Additionally, 3*O*MQ has been shown to be a flavone with high potential for nasal application, especially when combined with an absorption enhancer such as hydroxypropyl‐β‐cyclodextrin (HPβCD). This combination does not affect ciliary beat frequency (CBF) at 20 μg/mL, which is essential for ensuring the safety of nasal‐administered drugs (Dimova et al. 2003). Another example is troxerutin, a compound that has shown effects on rhinorrhea comparable to available treatments for the common cold. These findings suggest that such compounds could be considered a potential option for developing a safe and effective treatment for the common cold, avoiding the side effects of antihistaminic treatments (Turner et al. 2004).

### 8.2 Allergic Rhinitis

Allergic rhinitis is a chronic inflammatory condition of the nasal mucosa in hypersensitive individuals, mediated by immunoglobulin E (IgE) in response to exposure to various allergens. Once allergens stimulate antigen‐IgE production, the release of allergic mediators by mast cells, eosinophils, and T lymphocytes is triggered. These mediators, including histamine, cytokines, and chemokines, trigger various allergic symptoms, such as nasal itching, sneezing, and nasal discharge (Hu and Peng 2023).

Given that prolonged use of current treatments for allergic rhinitis can lead to side effects, such as endocrine disorders, liver and kidney damage, and central nervous system inhibition, it is crucial to seek new compounds that are more effective and offer higher pharmacological safety. An example is the flavonoid diosmetin, which has been shown to improve nasal symptoms, reduce nasal inflammation, and correct the Th1/Th2 cytokine imbalance by regulating the SIRT1/NF‐κB signaling pathway. Additionally, diosmetin negatively regulates serum levels of histamine, IgE, and IgG1, and attenuates eosinophil and mast cell infiltration in nasal mucosal tissues in mice with allergic rhinitis. This evidence suggests that this flavonoid could be a promising option for treating allergic rhinitis and other human allergic conditions (Hu and Peng 2023; Yu and Xu 2024). Similarly, isoorientin was shown to be a potential therapeutic agent due to its ability to restore the Th1/Th2 balance, inhibit the NF‐κB pathway in nasal tissues, and significantly decrease serum levels of IgE and histamine in mice (Huanga et al. 2024). Morin is another flavonoid that has shown significant ability to alleviate nasal symptoms, such as rubbing, sneezing, and discharge, in BALB/c mice sensitized with ovalbumen (OVA). This effect can be attributed to the inhibitory action of p‐STAT6, which negatively regulates the expression of the SOCS1 pathway and GATA3/T‐bet, thereby modulating the release of Th1/Th2 cytokines and IgE levels. This, in turn, helps to inhibit allergen‐induced hypersensitivity (Liang et al. 2019).

Another compound with potential anti‐allergic properties is apigenin. This flavonoid regulates the Th1/Th2 balance by suppressing the Th2 response, reducing the expression of IgE, histamine, IL, GATA3, STAT6, SOCS1, and NF‐κB, while activating the Th1 response by increasing IFN‐γ and T‐bet levels. Additionally, apigenin improves sneezing, rubbing, and discharge symptoms in rats at doses of 10 and 20 mg/kg (F. Chen et al. 2020). Additionally, apigenin can inhibit β‐hexosaminidase secretion and block LPS‐induced effects on cell viability, apoptosis, and inflammatory cytokine secretion by suppressing the TLR4/MyD88/NF‐κB signaling pathway in HMC‐1 cells. These findings confirm the flavonoid's ability to alleviate the inflammatory response in allergic rhinitis and suggest a potential mechanism of action for the compound (Li, Zhang, and Zhao 2023).

Similarly, baicalin has been shown to improve symptoms of allergic rhinitis and restore nasal mucosal function. Similar to other flavonoids, baicalin reduces serum levels of histamine, ECP, interleukin (IL)‐1β, IL‐6, IL‐8, tumor necrosis factor (TNF)‐α, and ovalbumin‐specific IgE in guinea pigs with OVA‐induced allergic rhinitis, as well as in vitro in human mast cells. Additionally, baicalin also suppressed the phosphorylation of JAK2, STAT5, IKKβ, IκBα, and the nuclear translocation of the NF‐κB (p65) subunit in LPS‐stimulated human mast cells. These effects demonstrate anti‐inflammatory activity, combined with findings on allergic rhinitis, that could position this flavonoid as an effective drug for treating such conditions (Y. Jang Zhou et al. 2016).

Hydroxysafflor yellow A is a chalcone that has also effectively reduced nasal allergy symptoms such as sneezing, rubbing, and redness. Additionally, it significantly decreased levels of Th2 cytokines and Th17 transcription factors, including the orphan receptor related to RAR gamma (ROR‐γ), signal transducer and activator of transcription 3 (STAT3), and phosphorylated signal transducer and activator of transcription 3 (p‐STAT3). At the same time, it increased levels of erythroid nuclear factor 2‐related factor 2 (Nrf2) and heme oxygenase‐1 (HO‐1). Similarly, this chalcone regulates the oxi‐red balance by reducing malondialdehyde (MDA) and enhancing levels of superoxide dismutase (SOD), glutathione peroxidase (GPx), catalase (CAT), and glutathione (GSH). These effects confirm its antioxidant and anti‐inflammatory properties and suggest therapeutic potential against ovalbumin‐induced allergic rhinitis in mice by altering the Th17/Treg balance and enhancing the Nrf2/HO‐1 signaling pathway (Ma et al. 2023).

On the other hand, the development of allergic diseases such as allergic rhinitis can be promoted by nitric oxide (NO), making this molecule a potential pharmacological target. Therefore, it is crucial to assess the influence of various compounds on NO production. In a study by Ebihara and colleagues, quercetin at a concentration as low as 1.0 nM suppressed NO production and IL‐4‐induced activation of the transcription factor STAT6. This finding indicates that quercetin may possess anti‐allergic properties, with this pathway among its therapeutic mechanisms (Ebihara et al. 2018).

Allergen‐induced stimulation generates sensory signals through the nerves, resulting in sensations such as itching and sneezing. Additionally, this stimulation triggers an axonal reflex that releases neuropeptides, such as substance P (SP) and calcitonin gene‐related peptide (CGRP). These neuropeptides are responsible for vasodilation, edema, and activation of inflammatory cells in the nasal mucosa, suggesting they could be potential therapeutic targets for allergic rhinitis. Therefore, compounds like quercetin, which have shown to inhibit increases in SP, CGRP, and nerve growth factor (NGF) levels when administered orally at 25 mg/kg for 5 days, can alleviate symptoms of nasal allergies induced by neurogenic inflammation. These characteristics indicate that quercetin is a potentially useful adjuvant for the management and treatment of allergic rhinitis (Kashiwabara et al. 2016).

Another crucial factor in the development of allergic rhinitis is the release of histamine. Therefore, its inhibition can be considered a pivotal point in the treatment of rhinitis. A compound with significant histamine‐inhibitory activity is luteolin‐7‐*O*‐glucoside, which has shown even better results than its precursor, luteolin. At 100 and 300 mg/kg, luteolin‐7‐*O*‐glucoside reduced nasal responses in ovalbumin‐sensitized rats, suggesting it could help alleviate nasal symptoms of allergic rhinitis, likely through the antigen–antibody reaction. These effects are primarily attributed to the catechol structure in ring B and the C2–C3 double bond in ring C. The sugar moiety at the C‐7 position of ring A may have contributed to the enhanced anti‐allergic activity of this compound, compared to luteolin (Inoue et al. 2002).

On the other hand, luteolin is an effective inhibitor of ANO1 activity in Calu‐3 cells. This calcium‐activated chloride channel is involved in nasal hypersecretion by increasing electrolyte secretion, thereby alleviating rhinorrhea, a symptom of allergic rhinitis. Consequently, luteolin may offer a new therapeutic approach for allergic rhinitis by regulating the ANO1 channel (Kim et al. 2020).

As previously noted, histamine H1 receptor (H1R) expression levels strongly correlate with the severity of allergic rhinitis symptoms. Therefore, suppressing H1R gene expression could be a relevant pharmacological target for anti‐allergic compounds. In this regard, quercetin has been shown to inhibit the histamine‐ and PMA‐induced upregulation of H1R gene expression. Additionally, it has been observed that this flavonol suppresses nasal allergy‐like symptoms induced by TDI and the elevation of H1R mRNA in the nasal mucosa of TDI‐sensitized rats. Therefore, it can be considered a flavonoid with anti‐allergic potential due to its suppressive effects and its ability to inhibit Th2 cytokine signaling through the histamine‐Th2 cytokine network. This leads to improved allergy symptoms and may help prevent the development of hay fever (Hattori et al. 2013).

### 8.3 Chronic Rhinosinusitis

Another condition primarily affecting the nasal cavity is chronic rhinosinusitis (CRS), a multifactorial inflammatory disease of the nose and paranasal sinuses that affects 12% of the global population (X. Han et al. 2023; H. W. Yang et al. 2018). The main symptoms of chronic rhinosinusitis include nasal obstruction, congestion, discharge, facial pain or pressure, impaired or lost sense of smell (anosmia), cough, and fatigue (Schleimer 2017; H. W. Yang et al. 2018). Although chronic rhinosinusitis is widespread, bacterial infection has been identified as one of the most common pathogens. However, the complete pathogenesis of the condition is not yet fully understood (Cheng et al. 2022; X. Han et al. 2023; H. W. Yang et al. 2018). Chronic rhinosinusitis is present in two forms: chronic rhinosinusitis without nasal polyps (CRSsNP) and chronic rhinosinusitis with nasal polyps (CRSwNP). Only 20%–30% of cases involve nasal polyps, which are characterized by chronic inflammation along with concurrent tissue remodeling (Schleimer 2017; Shi et al. 2013; H. W. Yang et al. 2018).

In chronic rhinosinusitis, tissue remodeling or reconstruction is characterized by excessive growth of the nasal mucosa, thickening of the basement membrane, glandular hyperplasia, and infiltration of inflammatory cells. In the development of chronic rhinosinusitis, transforming growth factor β1 (TGF‐β1) is involved. This profibrotic cytokine promotes the differentiation of nasal fibroblasts into myofibroblasts, thereby increasing the expression of extracellular matrix components, such as fibronectin and type I collagen. Therefore, fibroblasts and their differentiation are considered a crucial target for the treatment of chronic rhinosinusitis (Meng et al. 2013; Shi et al. 2013).

Based on this, flavonoids such as apigenin have been shown to exert anti‐remodeling effects on TGF‐β1‐stimulated nasal fibroblasts and inferior turbinate tissue. This is due to their ability to inhibit the phosphorylation of Mitogen‐activated protein kinase (MAPK) (p38, JNK) induced by TGF‐β1 and to block extracellular matrix production by inhibiting the NF‐κB transcription factor pathway. These processes are involved in the activation of myofibroblast differentiation and in the synthesis of the extracellular matrix (ECM). Additionally, this flavone attenuated fibroblast function by reducing collagen migration and contractile activity, suggesting that this flavonoid could contribute to the treatment and prevention of chronic rhinosinusitis (H. W. Yang et al. 2018).

An alternative therapeutic target of interest in chronic rhinosinusitis is the inflammatory response of tissues induced by lipopolysaccharides (LPS), a significant endotoxin from the outer membrane of Gram‐negative bacteria. On the other hand, specific miRNAs involved in the pathogenesis of chronic rhinosinusitis have been identified as miR‐21, which is associated with inflammatory disorders of the airways. MiR‐21 is known to regulate genes involved in the inflammatory response, making it a potential therapeutic target. Therefore, regulating genes whose expression promotes these effects may be essential for treating this condition. For example, quercetin increased miR‐21 expression, which, in turn, decreased DMBT1 and NF‐κB expression in human nasal epithelial cells (HNEpC). This effect is significant because the deleted gene in malignant brain tumors 1 (DMBT1) is directly involved in the growth and division of epithelial cells, and its expression is significantly elevated in cells exposed to LPS. This suggests that this flavonol could reduce the inflammatory response in LPS‐treated HNEpC cells and may provide a novel and effective therapeutic strategy for preventing or treating chronic rhinosinusitis (Cheng et al. 2022).

A flavonoid that stands out as a potentially effective alternative for the treatment of chronic rhinosinusitis is baicalin. This flavonoid dose‐dependently inhibits mast cell degranulation and significantly reduces IL‐33 expression in airway epithelial cells. Additionally, baicalin reduces type 2 predominant inflammation by negatively regulating the production of type 2 cytokines and the expression of IL‐5 and IL‐13 in human mast cells, through a combination of thymic stromal lymphopoietin (TSLP) and IL‐1β, and by stimulating IL‐33, respectively. This effect is of significant therapeutic importance due to the close relationship between type 2 inflammation and chronic rhinosinusitis, which typically presents with severe symptoms and a high recurrence rate after surgery. This makes regulating type 2 inflammation a primary therapeutic target, as it facilitates intense eosinophil infiltration into the respiratory tissue (Yoshida et al. 2021).

A potential pharmacological target for chronic rhinosinusitis is the expression of T2R14 in bronchial cilia and airway smooth muscle cells. Activation of a bitter taste receptor of taste family type 2 receptors, or T2Rs (T2R14) leads to Ca^2+^‐dependent NO production and an increase in ciliary beat frequency, which enhances mucociliary clearance and facilitates the cleaning of the respiratory epithelium. Thus, apigenin, chrysin, and wogonin, which have been shown to activate T2R14, also significantly repress the upregulation of Muc5AC and inducible nitric‐oxide synthase (iNOS), as well as cytokine release in airway cells in response to various inflammatory stimuli. This effect may be due to the inhibition of PKC and receptor tyrosine kinase activity. This suggests that these flavones may exert an anti‐inflammatory effect in airway cells and have clinical potential for topical therapies (Hariri et al. 2017).

### 8.4 Cystic Fibrosis

Cystic fibrosis (CF) is characterized by deficient or absent cAMP‐stimulated Cl^−^ conductance in various epithelia, including those of the nasal cavity, which are an important part of the respiratory tract (Illek et al. 1999). Mutations in the CFTR gene cause this genetic disease, which affects the epithelial chloride channel regulated by cyclic adenosine monophosphate (cAMP)‐dependent protein kinase A (PKA).

In general, several flavonoids have been shown to activate CFTR‐mediated Cl^−^ currents in the epithelium of human airways. Considering the stimulating concentrations, the ascending order of flavonoids would be: kaempferol ≤ apigenin ≤ genistein ≤ quercetin. In tissues treated with forskolin, sensitivity to flavonoids significantly increased, resulting in the following concentration sequence: kaempferol (2.5 ± 0.7 μM) ≤ apigenin (3.4 ± 0.9 μM) ≤ quercetin (4.1 ± 0.7 μM) ≤ genistein (6.9 ± 2.2 μM). Additionally, in vivo studies showed that flavonoids significantly increased nasal potential difference, achieving approximately 27.8% greater than with isoproterenol. This suggests that flavonoids could serve as potential lead compounds for the development of drugs aimed at CFTR activation (Illek and Fischer 1998).

An additional example is hesperidin, a flavonoid that has been shown to enhance Cl^−^ transport mediated by CFTR in both primary cultures of murine nasal septal epithelium (MNSE) and human sinonasal epithelium (HSNE). Similarly, in vivo, hesperidin significantly improves Cl^−^ transport across murine nasal epithelium and activates CBF in sinonasal epithelium. This suggests that it may be a promising therapeutic candidate for treating cystic fibrosis (Azbell et al. 2010).

One of the most common causes of CF is the deletion of phenylalanine at position 508 of the cystic fibrosis transmembrane conductance regulator (ΔF508 CFTR). As a result, improper folding of the protein product in the endoplasmic reticulum (ER) results in accelerated degradation in the proteasome. The aforementioned mutation is very common in CF; therefore, the detection of ΔF508 CFTR localized in the plasma membrane represents a logical target for treating the disease. Quercetin has been shown to activate ΔF508 CFTR in a dose‐dependent manner after its localization at the cell surface. Additionally, it promotes CFTR‐mediated anion transport in respiratory epithelia both in vitro and in vivo. This finding may be helpful in studies aimed at detecting and rescuing F508 CFTR using nasal potential difference measurements (Pyle et al. 2010). Additionally, quercetin could significantly increase the transepithelial transport of Cl^−^ and CBF in HSNE and MNSE cultures in both cystic and non‐cystic fibrosis cases. This highlights the pharmacological potential of quercetin for therapeutic use in the nasal cavity (S. Zhang et al. 2011).

As seen, the development of drugs targeting CFTR correctors (i.e., drugs that support the proper processing of the protein at the intracellular level) is of great importance for treating CF. Therefore, flavonoids such as 5,7,4′‐trimethoxyflavone, which has shown the ability to stimulate CFTR in vitro and in vivo, as well as to induce hyperpolarization of the nasal potential difference in humans, make it a reliable CFTR activator across different techniques (Fischer and Illek 2008). Additionally, in combination with cysteamine, epigallocatechin gallate (EGCG) reduces SQSTM1 levels and improves CFTR function in nasal epithelial cells in vivo. It was observed that EGCG was able to maintain the presence of a more significant amount of rescued ΔF508 CFTR in the plasma membrane after treatment with cysteamine. These findings suggest a potential synergistic effect between EGCG and cysteamine, which could be used to treat cystic fibrosis associated with the ΔF508 mutation in the CFTR gene (De Stefano et al. 2014).

The nonsense mutation of glycine to aspartic acid at codon 551 (G551D) is another mutation present in CF. In comparison to ΔF508‐CFTR, which is a mutation with impaired trafficking and whose synthesized protein is largely degraded intracellularly, G551D‐CFTR is transported to the apical membrane. However, the function of the chloride channel is significantly reduced. For this reason, it is also considered a potential therapeutic target. This is why compounds such as genistein, in the presence of forskolin, activated the G551D‐CFTR in such a way that its function was like that of the wild‐type CFTR under the same conditions. It also hyperpolarizes the nasal potential difference in humans, indicating activation of a Cl^−^ conductance under experimental conditions, as observed with the ΔF508 mutation of CFTR (Illek et al. 1999). Additionally, other studies have confirmed that genistein can activate the luminal Cl^−^ conductance of CFTR not only in mutants but also in tissues without cystic fibrosis (Mall et al. 2000). These findings suggest that genistein activates both CFTR and its mutants, making it an auspicious therapeutic approach for treating cystic fibrosis.

## 9 Targeting of Flavonoids to the Central Nervous System (Nose‐To‐Brain)

Unlike other routes of administration, nasal delivery allows medications to be absorbed through the nasal mucosa, thereby exerting both topical and systemic therapeutic and preventive effects. Nasal administration can result in a rapid systemic effect and, often, lower doses than oral administration, since it avoids first‐pass liver metabolism. While some molecules are absorbed systemically through the respiratory mucosa and enter the bloodstream, others may be targeted directly to the brain via the olfactory and trigeminal pathways. This administration route presents significant pharmacological interest for the treatment of various diseases, including those that do not directly involve the nasal cavity (Jeong et al. 2023).

**FIGURE 3:**
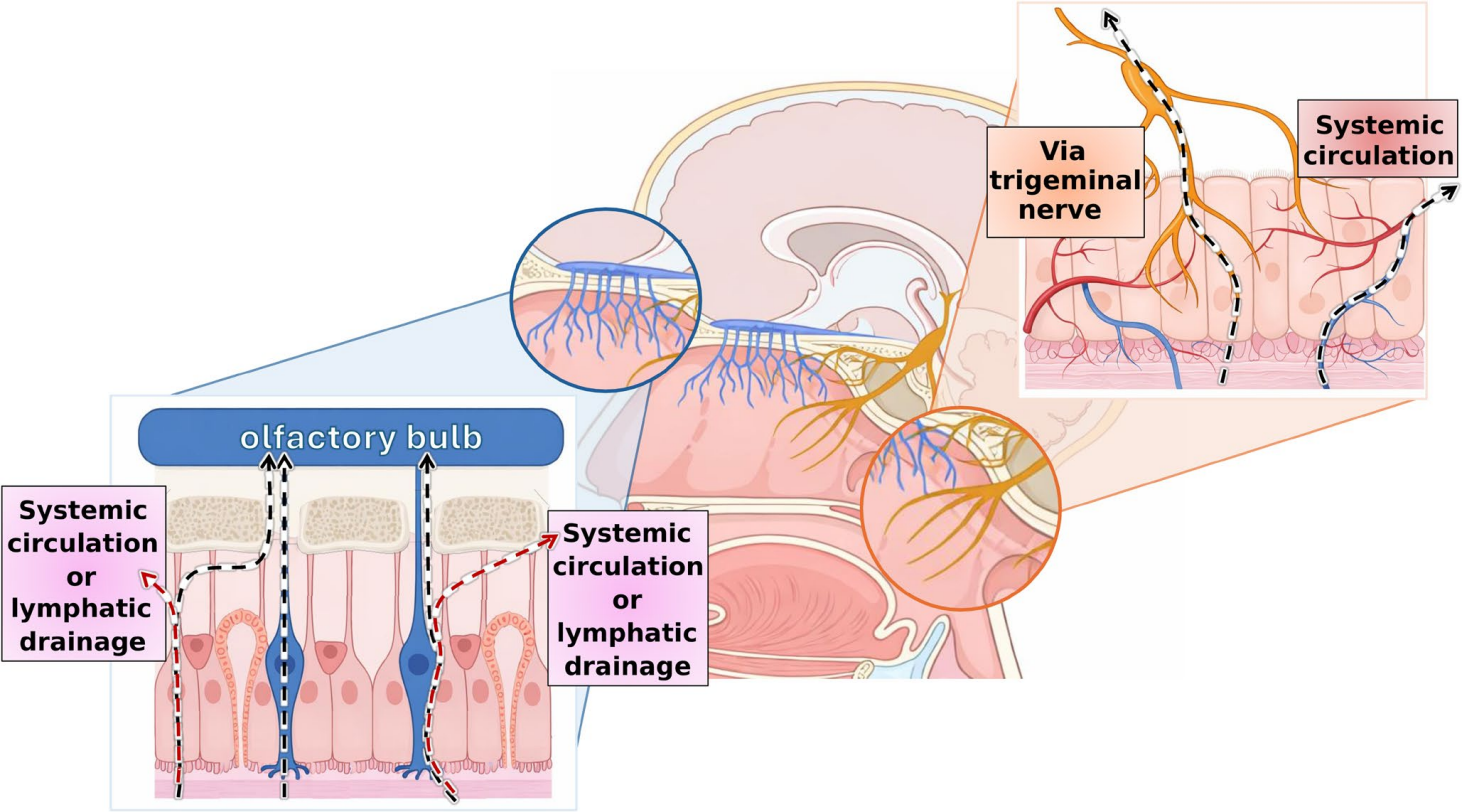
Mechanisms of transport and targeting of flavonoids from the nasal cavity to the brain.

Some molecules can also be directly targeted from the nasal cavity to the brain (nose‐to‐brain). Once the drug enters the nasal cavity, it may reach the brain through multiple pathways (Figure 3). When the therapeutic formulation targets the olfactory mucosa, the drug can access the lamina propria by crossing tight junctions within the olfactory epithelium, mainly formed by sustentacular cells, a process regulated by junctional proteins that control permeability and the overall tightness of the intercellular spaces. Additionally, endocytosis mediated by sustentacular cells and passive diffusion across the olfactory epithelium represent other relevant extraneuronal routes for drug transport (Crowe et al. 2018; Illum 2000). Once within the lamina propria, molecules may reach the olfactory bulb via the perineural space, traveling externally along neuronal axons as the extracellular fluid moves from the lamina propria toward the subarachnoid space. Subsequently, upon reaching the olfactory bulb, the drug can be distributed to different brain regions through the cerebrospinal fluid (CSF) (Crowe et al. 2018; Erdő et al. 2018; Selvaraj et al. 2018). However, the presence of nasal lymphatic vessels within the lamina propria allows, to a lesser extent, the transport of molecules to the brain via the meningeal lymphatic vessels (Crowe et al. 2018; Zhang, Niu, et al. 2025).

In addition to paracellular and transcellular transport pathways, the presence of neurons within the olfactory epithelium represents another relevant route for drug targeting. In this mechanism, endocytic vesicles containing the molecules are processed by the Golgi apparatus and converted into exosomes, which are subsequently transported along the neuronal axon, cross the cribriform plate, and reach the olfactory bulb. There, through exocytosis, the drug is released into postsynaptic cells of the olfactory bulb, enabling its subsequent distribution throughout the brain via the CSF (Figure 3) (Crowe et al. 2018; Erdő et al. 2018; Illum 2000; Selvaraj et al. 2018).

Another neural pathway that facilitates drug transport to the brain is the trigeminal nerve, which connects to the spinal cord, medulla oblongata, and pons. This large‐caliber nerve comprises two branches of particular relevance for nasal administration: the ophthalmic branch, whose nerve segments are partially located within the lamina propria and the olfactory bulb, and the maxillary branch, which, together with the ophthalmic branch, is predominantly distributed in the respiratory region of the nasal cavity (Finger et al. 2002; Illum 2000). From the nerve endings present in the epithelium of the respiratory region and within the lamina propria, molecules may be internalized via endocytosis and transported along the axonal pathway. Through the trigeminal route, drugs are conveyed to the trigeminal ganglion and then enter brainstem structures such as the pons. The arrival of the drug at these sites enables its subsequent release into the brain parenchyma, facilitated by fluid movement within the perivascular spaces (Figure 3) (Crowe et al. 2018; Dhuria et al. 2010; Erdő et al. 2018; Selvaraj et al. 2018).

Nevertheless, the high degree of vascularization in both the respiratory epithelium and the corresponding lamina propria allows a fraction of the drug to be absorbed into the systemic circulation. Once in the bloodstream, the molecules may cross the BBB and reach the central nervous system (CNS). However, there is a high likelihood that the drug will ultimately be eliminated through hepatic clearance mechanisms (Crowe et al. 2018; Illum 2000; Selvaraj et al. 2018).

The BBB represents a critical obstacle to the entry of active molecules into the central nervous system (CNS), as it separates the lumen of cerebral blood vessels from the brain parenchyma. This barrier function is primarily attributed to specialized endothelial cells, characterized by highly restrictive tight junctions that limit paracellular transport and markedly reduce endocytosis. In addition, the BBB expresses multiple efflux transporters that further restrict drug entry into the brain, including the ATP‐binding cassette P‐glycoprotein (P‐gp), neutral amino acid transporters, and the breast cancer resistance protein (BCRP). Collectively, these mechanisms account for the restricted delivery of more than 98% of molecules from the systemic circulation to the brain (Abbott 2013; Crowe et al. 2018; Illum 2000; Pandit et al. 2020; Selvaraj et al. 2018).

## 10 Factors Influencing the Transport of Flavonoids From the Nasal Cavity to the Brain

Despite the limitations of nasal administration, this route has been shown to offer significant advantages in terms of efficiency for the treatment of central nervous system (CNS) disorders compared with other routes. For instance, large molecules have been reported to reach the CNS at substantially higher concentrations following intranasal delivery (≈2 nM) than via the intravenous route (≈0.001 nM) (Frey et al. 1997).

Regarding flavonoids, intranasal administration of quercetin‐loaded gels has been shown to promote greater accumulation of the flavonoid in the brain compared with intravenous administration. Moreover, the incorporation of mucoadhesive agents into these formulations further enhances brain accumulation, achieving levels up to fivefold higher via the intranasal route than those obtained following intravenous delivery (Costa et al. 2021; Sonawane and Pokharkar 2024).

This effect is further evidenced by comparing the intranasal and oral routes: the former enable delivery of measurable amounts of the flavonol to the brain (28.9 μg/mL), whereas oral administration resulted in undetectable levels of quercetin and its metabolite in brain tissue (Manta et al. 2023). Similarly, other flavonoids, such as naringenin, have been reported to achieve significantly higher brain concentrations following intranasal administration (1148.6 ng/mL) than following intravenous delivery (381.7 ng/mL), using a vitamin E‐loaded naringenin nanoemulsion as the delivery vehicle (Gaba et al. 2019). These findings demonstrate that nasal administration is a potentially suitable and effective route for treating various central nervous system (CNS) disorders.

In this context, formulation strategies play a critical role in maximizing therapeutic outcomes. For example, Nagaraja and cols. (2021) demonstrated that a self‐emulsifying nanosystem of naringin incorporated in a gelling hydrogel can provide sustained delivery of the flavanone, due to the ability of the hydrogel to transition from a sol to a gel, thus overcoming the challenge of rapid nasomucociliar clearance. Consequently, this delivery system improved the permeation through sheep mucosa by approximately 67% compared to a simple flavonoid dispersion. Additionally, supplementary analyses have shown that the gelling naringin formulation is biocompatible and non‐toxic to the L929 mouse fibroblast cell line, and that it exhibits significantly higher drug transport and brain targeting efficiency in in vivo biodistribution studies. These properties of the in situ gelling system with naringin provided a new dimension to the treatment of chronic central nervous system disorders using small‐sized compounds, with the advantages of minimizing dosage and associated systemic adverse effects, as well as offering safe nasal administration (Nagaraja et al. 2021).

In more specific cases, such as stroke, phospholipid complexes with baicalin have been developed to transfer this flavonoid along the olfactory pathway to the brain following nasal administration in rats. This formulation allowed the flavonoid to enter brain tissues, with approximately 52.36%–100% of the baicalin content detected within 8 h after administration. This finding makes the nasal administration of BP‐phospholipid complexes a promising approach for protecting the brain against cerebral ischemic injury (Li, Ye, et al. 2011).

Various types of nanoemulsions loaded with kaempferol have also been developed to investigate their potential for brain delivery via the nasal route and to assess the antitumor capability of this flavonoid against glioma cells. In this context, chitosan was found to play a crucial role in enhancing the permeation of the flavonol through the mucosa and consequently increased the amount of drug in the rat brain after nasal administration by 5.0 and 4.5 times compared to free drug and nanoemulsion without the mucoadhesive polymer, respectively. This result represented an improvement in kaempferol's ability to inhibit the viability of C6 glioma cells through apoptosis induction, as determined by formulation and nasal administration (Colombo et al. 2018).

For neurodegenerative disorders such as Parkinson's disease, chitosan nanoparticles loaded with naringin (NAR‐NPs) have been designed, achieving a flavonoid permeation of 67.90% ± 0.72% through the nasal mucosa. Additionally, the nanoparticles demonstrated enhanced neuroprotective capability and antioxidant effects against 6‐hydroxydopamine (6‐OHDA) induced neurotoxicity in human neuroblastoma cells compared to the flavanone, providing initial evidence that they could be an effective carrier for Parkinson's disease treatment via the nose‐to‐brain route (Md et al. 2019). In relation to this disorder, mixtures of lyophilized powders with mannitol/lecithin microparticles (MLMP) containing quercetin complexed with HPβCD or methyl‐β‐cyclodextrin (MeβCD) were also proposed. These mixtures can enable significant levels of quercetin to reach both serum and brain, thereby improving brain targeting. Since quercetin transport is more efficient in the CNS when delivered in a formulation, its ‘nose‐to‐brain’ application is considered promising for the prevention and/or treatment of neurodegenerative and neuroinflammatory conditions, including Parkinson's disease, Alzheimer's disease, and other neurological disorders (Manta et al. 2023).

Also, Ahmed and cols have implemented the use of liposomes for the efficient administration of the flavonoid 7,8‐dihydroxyflavone, which, after its nasal application in a hemiparkinsonian mouse model, was observed to improve L‐DOPA‐induced dyskinesia (LID), specifically reducing the debilitating side effects of purposeless movements caused by long‐term use of replacement therapy with dopamine and L‐3,4‐dihydroxyphenylalanine (L‐DOPA). Additionally, it corrected some molecular signaling pathways involved in LID pathophysiology, suggesting it could be a promising alternative for Parkinson's disease (Ahmed et al. 2024).

For the treatment of Alzheimer disease, dihydroquercetin‐loaded chitosan nanoparticles have been developed and incorporated into a mucoadhesive thermosensitive in situ gel, which reduced both amyloid‐β protein formation and tau protein hyperphosphorylation, in addition to inhibiting acetylcholinesterase activity and oxidative stress in the brain. Consequently, improvements in the structures of the hippocampus and cerebral cortex in adult male Sprague–Dawley rats were observed, demonstrating the development of a promising therapeutic formulation for the treatment of Alzheimer disease (Abou‐Taleb et al. 2024).

In addition, nasal powders composed of lyophilized quercetin‐cyclodextrins (MeβCD and HPβCD) have shown improved apparent solubility of quercetin by 19 to 35 times when combined with each cyclodextrin, respectively. They showed permeation through regenerated cellulose membranes 17 times (for methyl‐β‐cyclodextrin) to 48 times (for HPβCD) that of pure quercetin, and this ratio was maintained in experiments through rabbit nasal mucosa (Papakyriakopoulou et al. 2021). Compared to other studies, the solubility of the lyophilized quercetin/MeβCD and quercetin/HPβCD products was 7–40 times and 14–50 times higher, respectively, than that of pure quercetin, across a pH range of 1.2–6.8. Regarding ex vivo permeation, the performance was 0.17 ± 0.04 and 0.22 ± 0.05 mg/cm^2^, respectively (Manta et al. 2020). These results provide strong evidence for the effectiveness of intranasal administration of quercetin formulations, suggesting the flavonol's potential neuroprotective activity by reducing oxidative stress and providing a basis for a possible therapeutic effect on Alzheimer's disease (Manta et al. 2020; Papakyriakopoulou et al. 2021).

## 11 Patents Regarding Flavonoid Nasal Delivery Systems

Although there is no approved nasal drug, various patents have explored and proposed innovative systems for the effective delivery of these compounds. These proposals encompass various administration routes, including the nasal route, to maximize bioavailability and therapeutic efficacy. Table 6 presents representative examples of these initiatives, highlighting their potential for developing flavonoid‐based delivery systems and their potential for targeting via the nasal route.

**TABLE 6:** Patents concerning nasal delivery systems containing flavonoids.

Patent no.	Inventor	Patent date	System used	References
US 20220202735 A1	Birbara, P. J.	June 30, 2022	The patent provides a new soluble and microparticulate formulations of flavonoids	Birbara 2022b
US 11491226 B2	Birbara, P. J.	January 07, 2021	The invention proposes novel soluble forms of organic compounds with a flat ring structure, including flavonoids, and a thermostable solubilizing compound	Birbara 2022a
ES 2847602 T3	Motoichi, K.; Nunnarpas, Y.; Ying, J. Y.; Chung, J. E.; Bae, K. H.; Tan, M.; Lee, E.	August 03, 2021	The patent shows a micellar nanocomplex comprising a micelle and an encapsulated agent, the micelle being a polymer‐flavonoid conjugate, where the polymer is bound to the B ring of the flavonoid	Motoichi et al. 2015
EP 3345594 B1	Birbara, P. J.	July 11, 2018	The patent refers to new soluble forms consisting of a flavonoid, a thermostable solubilizing compound, and a vehicle	Birbara 2018
CA 2669503 C	Day, B. J. and Kachadourian, R.	September 17, 2013	The patent shows methods for treating cystic fibrosis and other conditions, such as cancer, where flavonoids are used as agents to modulate the transport of thiol‐containing compounds.	Day and Kachadourian 2007
AU 2010203223 B2	Padrah, S. and Vajdy, M.	January 19, 2006	This patent provides adjuvant compositions that are capable of modulating the immune response in a subject, consisting of free mustard oil, a flavonoid, and a vitamin.	Padrah and Vajdy 2010
US 20060013905 A1	Tehoharides, T. C.	January 19, 2006	The patent reveals new compositions with synergistic anti‐inflammatory effects, consisting of 1 or more flavonoids, an interferon, other supplements, excipients, and appropriate vehicles.	Tehoharides 2006
US 7923043 B2	Theoharides, T. C.	April 12, 2011	The patent provides a composition to treat chronic fatigue syndrome, consisting of a flavonoid compound, vitamin B12, other appropriate supplements, excipients, and vehicles.	Theoharides 2009

## 12 Conclusion

This review highlights that although the oral route remains the most established pathway for flavonoid administration, the aglycones exhibit biological and physicochemical properties that make it particularly attractive for alternative administration routes, such as the nasal route. Moreover, the intranasal route is a promising strategy to overcome key limitations of oral administration, such as extensive first‐pass metabolism and low aqueous solubility.

The role of advanced drug delivery systems is critical to enable effective intranasal administration of flavonoids. Nanostructured systems, micelles, cyclodextrin complexes, and mucoadhesive or thermosensitive polymers have demonstrated their ability to enhance permeation, protect flavonoids from premature degradation, improve their aqueous solubility, or prolong nasal residence time. Consequently, these approaches can potentiate both their effects.

The available scientific evidence supports intranasal administration as a versatile and potentially transformative route for flavonoid‐based therapies, offering opportunities for local, systemic, and direct nose‐to‐brain delivery. However, the full realization of this therapeutic approach still depends on addressing several unresolved scientific and technological challenges.

## 13 Outlook and Future Perspectives

Future research on intranasal flavonoid delivery should prioritize the development of intranasal flavonoid drugs requires a clear prioritization of research and regulatory efforts. A still unresolved need is the characterization of the pharmacokinetic profile and drug biodistribution following nasal administration, particularly systemic absorption/distribution and direct nose‐to‐brain transport mechanisms. Several studies have highlighted the importance of understanding these processes to optimize therapeutic outcomes, as significant differences in drug absorption kinetics have been observed between in vivo animal experimental models and humans following inhalation administration (Kolanjiyil et al. 2019; Lowe et al. 2017).

To address these limitations, future experimental designs could adopt multilevel approaches incorporating in vitro, ex vivo, and in vivo models. Advanced in vitro systems, such as differentiated epithelial cell cultures and enhanced mucosal models, provide detailed insights into permeation mechanisms, mucoadhesion, and local toxicity (H. Wang et al. 2018; Wengst and Reichl 2010). On the other hand, ex vivo and in vivo studies remain essential to validate nose‐to‐brain transport and therapeutic efficacy by correlating drug interactions with the various tissues along this route (H. Li et al. 2025). In this regard, it is crucial to conduct standardized comparative studies evaluating flavonoids in both aglycone and glycoside forms under equivalent intranasal conditions to guide the rational design of formulations.

On the other hand, technologies are emerging as tools to accelerate development in this field. Microfluidics‐based platforms and in vitro nasal mucosa models with enhanced physiological relevance allow the simulation of processes such as mucociliary clearance and formulation–tissue interactions, improving predictive capability and reducing reliance on animal models (Koch et al. 2023; Kreft et al. 2015). Additionally, advances in systems designed to target deposition to specific regions, such as the olfactory epithelium, could significantly enhance dosing precision and the effectiveness of direct nose‐to‐brain delivery (Seifelnasr et al. 2023; Zhang, Su, et al. 2025).

From a translational perspective, early integration of safety considerations, long‐term nasal tolerability, excipient compatibility, and manufacturing process reproducibility will be essential for successful clinical development. Aligning formulation strategies with regulatory requirements from the initial stages could facilitate the progression of flavonoid‐based intranasal therapies toward clinical application. Ultimately, systematically addressing these limitations will be crucial to unlocking the full therapeutic potential of intranasally administered flavonoids.

## 14 Open Questions

From the analysis of the articles included in the review on nasal administration of flavonoids and their impact on the protection of the nasal cavity, the treatment of diseases affecting this site, and their therapeutic potential for neurodegenerative diseases and brain tumors, several knowledge gaps, barriers to clinical application, and possible solutions have been identified. The main open questions are presented below:

### 14.1 Knowledge Gaps

*How does the bioavailability of nasally administered flavonoids vary depending on their chemical structure and the physiological characteristics of the nasal mucosa?

*What are the factors that influence the transport and metabolism of flavonoids from the nasal cavity to the brain?

*How do flavonoid formulations impact the nasal microbiota and their role in modulating inflammation and immune response?

### 14.2 Main Barriers to Clinical Application

Despite promising preclinical results, few clinical trials have validated the efficacy and safety of flavonoids delivered via the nasal route for respiratory and neurological diseases. In addition, the lack of standardization in the production and formulation of flavonoids complicates their approval for clinical use.

On the other hand, factors such as rapid mucociliary clearance and enzymatic degradation can reduce the therapeutic efficacy of flavonoids administered by this route.

### 14.3 Possible Solutions

As shown, one solution for nose‐to‐brain therapy is the incorporation of nanoparticles, sustained‐release systems, and bioadhesive carriers, which could improve the stability and bioavailability of flavonoids in the nasal cavity. Also, the structural modification of flavonoids or combination with nasal permeabilizers to enhance their absorption and transport to the central nervous system.

Other potential solutions include expanding studies in animal models, moving toward clinical trials to validate therapeutic efficacy and safety profiles, and developing clear guidelines and conducting pharmacokinetic and pharmacodynamic studies to facilitate the approval of nasal formulations of flavonoids as viable therapies.

These issues represent critical points requiring further research and development so that nasally administered flavonoid‐based therapies can reach the clinic and become effective treatments for respiratory and neurodegenerative diseases.

## Author Contributions

**Jeniffer Viviana Ramirez Hernández:** conceptualization and writing – original draft. **Sara Elis Bianchi:** investigation, writing, review, and editing. **Flávia Nathiely Silveira Fachel:** review and editing. **Elizandra Braganhol:** investigation and supervision. **Sheila Porto de Matos:** writing, review, and editing. **Valquíria Linck Bassani:** conceptualization, funding acquisition, project administration, writing – review and editing.

## Funding

This work was supported by the Coordenação de Aperfeiçoamento de Pessoal de Nível Superior (CAPES, 001); Conselho Nacional de Desenvolvimento Científico e Tecnológico (CNPq) (305490/2022‐2 and 406218/2025‐0).

## Conflicts of Interest

The authors declare no conflicts of interest.

## Data Availability

Data sharing not applicable to this article as no datasets were generated or analysed during the current study.

## References

[ptr70380-bib-0001] Abbott, N. J. 2013. “Blood‐Brain Barrier Structure and Function and the Challenges for CNS Drug Delivery.” Journal of Inherited Metabolic Disease 36, no. 3: 437–449. 10.1007/s10545-013-9608-0. 23609350

[ptr70380-bib-0002] Abdel‐Ghaffar, O. , S. T. Mahmoud , A. A. Said , and F. A. A. Y. Sanad . 2018. “Ameliorative Effect of Rutin Against Isoniazid‐Induced Alterations in Certain Hematological and Biochemical Parameters of Albino Rats.” International Journal of Pharmacology 14, no. 1: 39–51. 10.3923/ijp.2018.39.51.

[ptr70380-bib-0003] Abou‐Taleb, B. A. , W. F. El‐Hadidy , I. M. Masoud , N. A. Matar , and H. S. Hussein . 2024. “Dihydroquercetin Nanoparticles Nasal Gel Is a Promising Formulation for Amelioration of Alzheimer's Disease.” International Journal of Pharmaceutics 666: 1–17. 10.1016/j.ijpharm.2024.124814. 39384026

[ptr70380-bib-0004] Abrahamse, S. L. , W. J. Kloots , and J. M. M. Van Amelsvoort . 2005. “Absorption, Distribution, and Secretion of Epicatechin and Quercetin in the Rat.” Nutrition Research 25, no. 3: 305–317. 10.1016/j.nutres.2004.10.013.

[ptr70380-bib-0005] Actis‐Goretta, L. , A. Lévèques , F. Giuffrida , et al. 2012. “Elucidation of (−)‐Epicatechin Metabolites After Ingestion of Chocolate by Healthy Humans.” Free Radical Biology and Medicine 53, no. 4: 787–795. 10.1016/j.freeradbiomed.2012.05.023. 22664313

[ptr70380-bib-0006] Ahmed, M. R. , M. Inayathullah , M. Morton , et al. 2024. “Intranasal Delivery of Liposome Encapsulated Flavonoids Ameliorates L‐DOPA Induced Dyskinesia in Hemiparkinsonian Mice.” Biomaterials 311: 1–21. 10.1016/j.biomaterials.2024.122680. 38959534

[ptr70380-bib-0007] Alghareeb, S. , K. Asare‐Addo , B. R. Conway , and A. O. Adebisi . 2024. “PLGA Nanoparticles for Nasal Drug Delivery.” Journal of Drug Delivery Science and Technology 95: 1–25. 10.1016/j.jddst.2024.105564.

[ptr70380-bib-0008] Alkhalidy, H. , W. Moore , Y. Wang , et al. 2018. “The Flavonoid Kaempferol Ameliorates Streptozotocin‐Induced Diabetes by Suppressing Hepatic Glucose Production.” Molecules 23, no. 9: 1–16. 10.3390/molecules23092338. PMC6192519 30216981

[ptr70380-bib-0009] Álvarez, A. I. , F. Vallejo , B. Barrera , et al. 2011. “Bioavailability of the Glucuronide and Sulfate Conjugates of Genistein and Daidzein in Breast Cancer Resistance Protein 1 Knockout Mice.” Drug Metabolism and Disposition 39, no. 11: 2008–2012. 10.1124/dmd.111.040881. 21828252

[ptr70380-bib-0010] Azbell, C. , S. Zhang , D. Skinner , J. Fortenberry , E. J. Sorscher , and B. A. Woodworth . 2010. “Hesperidin Stimulates Cystic Fibrosis Transmembrane Conductance Regulatormediated Chloride Secretion and Ciliary Beat Frequency in Sinonasal Epithelium.” Otolaryngology–Head and Neck Surgery 143, no. 3: 397–404. 10.1016/j.otohns.2010.05.021. 20723778 PMC3073343

[ptr70380-bib-0011] Bai, H. H. , T. S. Xia , Y. P. Jiang , et al. 2022. “Absorption, Metabolism, and Pharmacokinetic Profile of Xanthohumol in Rats as Determined via UPLC‐MS/MS.” Biopharmaceutics and Drug Disposition 43, no. 1: 11–22. 10.1002/bdd.2306. 34914109

[ptr70380-bib-0012] Bai, X. , J. Qu , J. Lu , Y. Kano , and D. Yuan . 2011. “Pharmacokinetics of Kakkalide and Its Main Metabolites in Rat Plasma Determined by HPLC‐DAD and LC‐MSn.” Journal of Chromatography B: Analytical Technologies in the Biomedical and Life Sciences 879, no. 5–6: 395–402. 10.1016/j.jchromb.2010.12.025. 21256817

[ptr70380-bib-0013] Bariexca, T. , J. Ezdebski , B. W. Redan , and J. Vinson . 2019. “Pure Polyphenols and Cranberry Juice High in Anthocyanins Increase Antioxidant Capacity in Animal Organs.” Food 8, no. 8: 1–10. 10.3390/foods8080340. PMC6727083 31408979

[ptr70380-bib-0014] Berga, M. , K. Logviss , L. Lauberte , A. Paulausks , and V. Mohylyuk . 2023. “Flavonoids in the Spotlight: Bridging the Gap Between Physicochemical Properties and Formulation Strategies.” Pharmaceuticals 16, no. 10: 1–28. 10.3390/ph16101407. PMC10610233 37895878

[ptr70380-bib-0015] Berrin, J. G. , W. R. McLauchlan , P. Needs , et al. 2002. “Functional Expression of Human Liver Cytosolic β‐Glucosidase in Pichia Pastoris: Insights Into Its Role in the Metabolism of Dietary Glucosides.” European Journal of Biochemistry 269, no. 1: 249–258. 10.1046/j.0014-2956.2001.02641.x. 11784319

[ptr70380-bib-0016] Bicker, J. , A. Fortuna , G. Alves , and A. Falcão . 2020. “Nose‐To‐Brain Delivery of Natural Compounds for the Treatment of Central Nervous System Disorders.” Current Pharmaceutical Design 26, no. 5: 594–619. 10.2174/1381612826666200115101544. 31939728

[ptr70380-bib-0017] Birbara, P. J. 2018. “Solubilized Flavonoid Composition (Patent EP3345594B1).”

[ptr70380-bib-0018] Birbara, P. J. 2022a. “Methods of Increasing Solubility of Poorly Soluble Compounds and Methods of Making and Using Formulations of Such Compound (Patent US11491226B2).” http://www.oogroup.it/sooft/en/.

[ptr70380-bib-0019] Birbara, P. J. 2022b. “Methods of Making and Using Compositions Comprising Flavonoids (Patent US 20220202735A1).”

[ptr70380-bib-0020] Bordenave, N. , B. R. Hamaker , and M. G. Ferruzzi . 2014. “Nature and Consequences of Non‐Covalent Interactions Between Flavonoids and Macronutrients in Foods.” Food and Function 5, no. 1: 18–34. 10.1039/c3fo60263j. 24326533

[ptr70380-bib-0021] Boyuklieva, R. , and B. Pilicheva . 2022. “Micro‐ and Nanosized Carriers for Nose‐To‐Brain Drug Delivery in Neurodegenerative Disorders.” Biomedicine 10, no. 7: 1–18. 10.3390/biomedicines10071706. PMC9313014 35885011

[ptr70380-bib-0022] Cai, H. , D. J. Boocock , W. P. Steward , and A. J. Gescher . 2007. “Tissue Distribution in Mice and Metabolism in Murine and Human Liver of Apigenin and Tricin, Flavones With Putative Cancer Chemopreventive Properties.” Cancer Chemotherapy and Pharmacology 60, no. 2: 257–266. 10.1007/s00280-006-0368-5. 17089164

[ptr70380-bib-0023] Casanova, V. , F. H. Sousa , C. Stevens , and P. G. Barlow . 2018. “Antiviral Therapeutic Approaches for Human Rhinovirus Infections.” Future Virology 13, no. 7: 505–518. 10.2217/fvl-2018-0016. 30245735 PMC6136076

[ptr70380-bib-0024] Cassidy, A. , and A. M. Minihane . 2017. “The Role of Metabolism (And the Microbiome) in Defining the Clinical Efficacy of Dietary Flavonoids.” American Journal of Clinical Nutrition 105, no. 1: 10–22. 10.3945/ajcn.116.136051. 27881391 PMC5183723

[ptr70380-bib-0025] Castellano, G. , J. L. González‐Santander , A. Lara , and F. Torrens . 2013. “Classification of Flavonoid Compounds by Using Entropy of Information Theory.” Phytochemistry 93: 182–191. 10.1016/j.phytochem.2013.03.024. 23642389

[ptr70380-bib-0026] Catterall, F. , L. J. King , M. N. Clifford , and C. Ioannides . 2003. “Bioavailability of Dietary Doses of 3H‐Labelled Tea Antioxidants (+)‐Catechin and (−)‐Epicatechin in Rat.” Xenobiotica 33, no. 7: 743–753. 10.1080/0049825031000108315. 12893523

[ptr70380-bib-0027] Çelik, Y. S. , B. Örenli , M. Al‐Mohaya , B. Mesut , and Y. Özsoy . 2023. “Nasal In Situ Gels as a Drug Delivery System: An Overview of Literature and Clinical Studies.” Journal of Research in Pharmacy 27, no. 5: 1875–1888. 10.29228/jrp.471.

[ptr70380-bib-0028] Chavda, V. P. , A. Z. Chaudhari , P. C. Balar , A. Gholap , and L. K. Vora . 2024. “Phytoestrogens: Chemistry, Potential Health Benefits, and Their Medicinal Importance.” Phytotherapy Research 38, no. 6: 3060–3079. 10.1002/ptr.8196. 38602108

[ptr70380-bib-0029] Chen, F. , D. He , and B. Yan . 2020. “Apigenin Attenuates Allergic Responses of Ovalbumin‐Induced Allergic Rhinitis Through Modulation of Th1/Th2 Responses in Experimental Mice.” Dose‐Response 18, no. 1: 1559325820904799. 10.1177/1559325820904799. 32165873 PMC7054738

[ptr70380-bib-0030] Chen, S. , X. Wang , Y. Cheng , H. Gao , and X. Chen . 2023. “A Review of Classification, Biosynthesis, Biological Activities and Potential Applications of Flavonoids.” Molecules 28, no. 13: 4982. 10.3390/molecules28134982. 37446644 PMC10343696

[ptr70380-bib-0031] Chen, X. , L. Cui , X. Duan , B. Ma , and D. Zhong . 2006. “Pharmacokinetics and Metabolism of the Flavonoid Scutellarin in Humans After a Single Oral Administration.” Drug Metabolism and Disposition 34, no. 8: 1345–1352. 10.1124/dmd.106.009779. 16714374

[ptr70380-bib-0032] Chen, X. , N. Gu , C. Xue , and B. R. Li . 2018. “Plant Flavonoid Taxifolin Inhibits the Growth, Migration and Invasion of Human Osteosarcoma Cells.” Molecular Medicine Reports 17, no. 2: 3239–3245. 10.3892/mmr.2017.8271. 29257319

[ptr70380-bib-0033] Chen, X. , O. Q. P. Yin , Z. Zuo , and M. S. S. Chow . 2005. “Pharmacokinetics and Modeling of Quercetin and Metabolites.” Pharmaceutical Research 22, no. 6: 892–901. 10.1007/s11095-005-4584-1. 15948033

[ptr70380-bib-0034] Chen, Z. , M. Tu , S. Sun , et al. 2012. “The Exposure of Luteolin Is Much Lower Than That of Apigenin in Oral Administration of Flos Chrysanthemi Extract to Rats.” Drug Metabolism and Pharmacokinetics 27, no. 1: 162–168. 10.2133/dmpk.DMPK-11-RG-081. 21931223

[ptr70380-bib-0035] Chen, Z. , S. Zheng , L. Li , and H. Jiang . 2014. “Metabolism of Flavonoids in Human: A Comprehensive Review.” Current Drug Metabolism 15: 48–61. 24588554 10.2174/138920021501140218125020

[ptr70380-bib-0036] Cheng, J. , X. g. Luo , and F. s. Chen . 2022. “Quercetin Attenuates Lipopolysaccharide‐Mediated Inflammatory Injury in Human Nasal Epithelial Cells via Regulating miR‐21/DMBT1/NF‐κB Axis.” Immunopharmacology and Immunotoxicology 44, no. 1: 7–16. 10.1080/08923973.2021.1988963. 34927513

[ptr70380-bib-0037] Cho, D. Y. , R. C. Hunter , and V. R. Ramakrishnan . 2020. “The Microbiome and Chronic Rhinosinusitis.” Immunology and Allergy Clinics of North America 40, no. 2: 251–263. 10.1016/j.iac.2019.12.009. 32278449 PMC7154041

[ptr70380-bib-0038] Choi, S. J. , and D. J. McClements . 2020. “Nanoemulsions as Delivery Systems for Lipophilic Nutraceuticals: Strategies for Improving Their Formulation, Stability, Functionality and Bioavailability.” Food Science and Biotechnology 29, no. 2: 149–168. 10.1007/s10068-019-00731-4. 32064124 PMC6992823

[ptr70380-bib-0039] Chung, S. , J. M. Peters , K. Detyniecki , W. Tatum , A. L. Rabinowicz , and E. Carrazana . 2023. “The Nose Has It: Opportunities and Challenges for Intranasal Drug Administration for Neurologic Conditions Including Seizure Clusters.” Epilepsy and Behavior Reports 21: 1–12. 10.1016/j.ebr.2022.100581. PMC9829802 36636458

[ptr70380-bib-0040] Cingi, C. , N. Bayar Muluk , D. I. Mitsias , et al. 2021. “The Nose as a Route for Therapy: Part 1. Pharmacotherapy.” Frontiers in Allergy 2: 1–17. 10.3389/falgy.2021.638136. PMC8974766 35387039

[ptr70380-bib-0041] Colombo, M. , F. Figueiró , A. de Fraga Dias , H. F. Teixeira , A. M. O. Battastini , and L. S. Koester . 2018. “Kaempferol‐Loaded Mucoadhesive Nanoemulsion for Intranasal Administration Reduces Glioma Growth In Vitro.” International Journal of Pharmaceutics 543: 214–223. 10.1016/j.ijpharm.2018.03.055. 29605695

[ptr70380-bib-0042] Cos, P. , L. Ying , M. Calomme , et al. 1998. “Structure‐Activity Relationship and Classification of Flavonoids as Inhibitors of Xanthine Oxidase and Superoxide Scavengers.” Journal of Natural Products 61, no. 1: 71–76. 9461655 10.1021/np970237h

[ptr70380-bib-0043] Costa, C. P. , J. N. Moreira , J. M. Sousa Lobo , and A. C. Silva . 2021. “Intranasal Delivery of Nanostructured Lipid Carriers, Solid Lipid Nanoparticles and Nanoemulsions: A Current Overview of In Vivo Studies.” Acta Pharmaceutica Sinica B 11, no. 4: 925–940. 10.1016/j.apsb.2021.02.012. 33996407 PMC8105874

[ptr70380-bib-0044] Crespy, V. , C. Morand , C. Besson , C. Manach , C. Demigne , and C. Remesy . 2002. “Quercetin, but Not Its Glycosides, Is Absorbed From the Rat Stomach.” Journal of Agricultural and Food Chemistry 50, no. 3: 618–621. 10.1021/jf010919h. 11804539

[ptr70380-bib-0045] Crowe, T. P. , M. H. W. Greenlee , A. G. Kanthasamy , and W. H. Hsu . 2018. “Mechanism of Intranasal Drug Delivery Directly to the Brain.” Life Sciences 195: 44–52. 10.1016/j.lfs.2017.12.025. 29277310

[ptr70380-bib-0046] Dahiya, A. , C. Majee , R. Mazumder , et al. 2023. “Insight Into the Glycosylation Methods of the Flavonoids as an Approach to Enhance Its Bioavailability and Pharmacological Activities.” Indian Journal of Pharmaceutical Education and Research 57, no. 2: 354–371. 10.5530/ijper.57.2.45.

[ptr70380-bib-0047] Dahl, A. R. , and W. M. Hadley . 1991. “Nasal Cavity Enzymes Involved in Xenobiotic Metabolism: Effects on the Toxicity of Inhalants.” Toxicology 21, no. 5: 345–372. 10.3109/10408449109019571 1741949

[ptr70380-bib-0048] Day, A. J. , F. J. Cañada , J. C. Díaz , et al. 2000. “Dietary Flavonoid and Isoflavone Glycosides Are Hydrolysed by the Lactase Site of Lactase Phlorizin Hydrolase.” FEBS Letters 468, no. 2–3: 166–170. 10.1016/S0014-5793(00)01211-4. 10692580

[ptr70380-bib-0049] Day, B. J. , and R. Kachadourian . 2007. “Compounds and Methods for Thiol‐Containing Compound Efflux and Cancer Treatment (Patent CA2669503C).”

[ptr70380-bib-0050] Dayem, A. A. , H. Y. Choi , Y. B. Kim , and S. G. Cho . 2015. “Antiviral Effect of Methylated Flavonol Isorhamnetin Against Influenza.” PLoS One 10, no. 3: 1–21. 10.1371/journal.pone.0121610. PMC4373826 25806943

[ptr70380-bib-0051] de Steenhuijsen Piters, W. A. A. , E. A. M. Sanders , and D. Bogaert . 2015. “The Role of the Local Microbial Ecosystem in Respiratory Health and Disease.” Philosophical Transactions of the Royal Society, B: Biological Sciences 370, no. 1675: 20140294. 10.1098/rstb.2014.0294. PMC4528492 26150660

[ptr70380-bib-0052] De Stefano, D. , V. R. Villella , S. Esposito , et al. 2014. “Restoration of CFTR Function in Patients With Cystic Fibrosis Carrying the F508del‐CFTR Mutation.” Autophagy 10, no. 11: 2053–2074. 10.4161/15548627.2014.973737. 25350163 PMC4502695

[ptr70380-bib-0053] Demrow, H. S. , P. R. Slane , and J. D. Folts . 1995. “Administration of Wine and Grape Juice Inhibits In Vivo Platelet Activity and Thrombosis in Stenosed Canine Coronary Arteries.” Circulation 91, no. 4: 1182–1188. 10.1161/01.CIR.91.4.1182. 7850957

[ptr70380-bib-0054] Desai, N. 2012. “Challenges in Development of Nanoparticle‐Based Therapeutics.” AAPS Journal 14, no. 2: 282–295. 10.1208/s12248-012-9339-4. 22407288 PMC3326161

[ptr70380-bib-0055] Dhuria, S. V. , L. R. Hanson , and W. H. Frey . 2010. “Intranasal Delivery to the Central Nervous System: Mechanisms and Experimental Considerations.” Journal of Pharmaceutical Sciences 99, no. 4: 1654–1673. 10.1002/jps.21924. 19877171

[ptr70380-bib-0056] Dimova, S. , R. Mugabowindekwe , T. Willems , et al. 2003. “Safety‐Assessment of 3‐Methoxyquercetin as an Antirhinoviral Compound for Nasal Application: Effect on Ciliary Beat Frequency.” International Journal of Pharmaceutics 263, no. 1–2: 95–103. 10.1016/S0378-5173(03)00363-6. 12954184

[ptr70380-bib-0057] Dixon, R. A. , and C. L. Steele . 1999. “Flavonoids and Isoflavonoids ‐ a Gold Mine for Metabolic Engineering.” Trends in Plant Science 4, no. 10: 394–400. 10498963 10.1016/s1360-1385(99)01471-5

[ptr70380-bib-0058] Dong, P. , L. Shi , S. Wang , et al. 2021. “Rapid Profiling and Identification of Vitexin Metabolites in Rat Urine, Plasma and Faeces After Oral Administration Using a UHPLC‐Q‐Exactive Orbitrap Mass Spectrometer Coupled With Multiple Data‐Mining Methods.” Current Drug Metabolism 22, no. 3: 185–197. 10.2174/1389200221999210101232841. 33397253

[ptr70380-bib-0059] Dong, X. , Y. Liu , J. Yan , et al. 2008. “Identification of SVM‐Based Classification Model, Synthesis and Evaluation of Prenylated Flavonoids as Vasorelaxant Agents.” Bioorganic and Medicinal Chemistry 16, no. 17: 8151–8160. 10.1016/j.bmc.2008.07.031. 18678502

[ptr70380-bib-0060] Duong, V. A. , T. T. L. Nguyen , and H. J. Maeng . 2023. “Recent Advances in Intranasal Liposomes for Drug, Gene, and Vaccine Delivery.” Pharmaceutics 15, no. 1: 1–27. 10.3390/pharmaceutics15010207. PMC9865923 36678838

[ptr70380-bib-0061] Ebihara, N. , K. Takahashi , H. Takemura , Y. Akanuma , K. Asano , and M. Sunagawa . 2018. “Suppressive Effect of Quercetin on Nitric Oxide Production From Nasal Epithelial Cells In Vitro.” Evidence‐Based Complementary and Alternative Medicine 2018: 6097625. 10.1155/2018/6097625. 30069224 PMC6057307

[ptr70380-bib-0062] Echeverría, J. , J. Opazo , L. Mendoza , A. Urzúa , and M. Wilkens . 2017. “Structure‐Activity and Lipophilicity Relationships of Selected Antibacterial Natural Flavones and Flavanones of Chilean Flora.” Molecules 22, no. 4: 1–15. 10.3390/molecules22040608. PMC6154607 28394271

[ptr70380-bib-0063] Erdő, F. , L. A. Bors , D. Farkas , Á. Bajza , and S. Gizurarson . 2018. “Evaluation of Intranasal Delivery Route of Drug Administration for Brain Targeting.” Brain Research Bulletin 143: 155–170. 10.1016/j.brainresbull.2018.10.009. 30449731

[ptr70380-bib-0064] Erik, Z. , İ. Erik , C. Ö. Yalçin , et al. 2025. “Evaluation of Synthesized Methoxy Chalcones for Therapeutic Potential Through In Vitro and In Silico Methods.” Journal of Research in Pharmacy 29, no. 4: 1693–1711. 10.12991/jrespharm.1734661.

[ptr70380-bib-0065] European Medicines Agency . 2025. “Guideline on the Pharmaceutical Quality of Inhalation and Nasal Medicinal Products.” https://www.ema.europa.eu/contact.

[ptr70380-bib-0066] Fang, J. 2014a. “Bioavailability of Anthocyanins.” Drug Metabolism Reviews 46, no. 4: 508–520. 10.3109/03602532.2014.978080. 25347327

[ptr70380-bib-0067] Fang, J. 2014b. “Some Anthocyanins Could Be Efficiently Absorbed Across the Gastrointestinal Mucosa: Extensive Presystemic Metabolism Reduces Apparent Bioavailability.” Journal of Agricultural and Food Chemistry 62, no. 18: 3904–3911. 10.1021/jf405356b. 24650097

[ptr70380-bib-0068] Finger, T. E. , B. Böttger , M. L. Schaefer , and W. L. Silver . 2002. “Trigeminal Collaterals in the Nasal Epithelium and Olfactory Bulb: A Potential Route for Direct Modulation of Olfactory Information by Trigeminal Stimuli.” Journal of Comparative Neurology 444, no. 3: 221–226. 10.1002/cne.10143. 11840476

[ptr70380-bib-0069] Fischer, H. , and B. Illek . 2008. “Activation of the CFTR cl–Channel by Trimethoxyflavone In Vitro and In Vivo.” Cellular Physiology and Biochemistry 22, no. 5–6: 685–692. 10.1159/000185552. 19088450 PMC2820299

[ptr70380-bib-0070] Food and Drug Administration . 2002. “Nasal Spray and Inhalation Solution, Suspension, and Spray Drug Products Chemistry, Manufacturing, and Controls Documentation FDA. U.S. Department of Health and Human Services.”

[ptr70380-bib-0071] Food and Drug Administration . 2003. “Guidance for Industry Bioavailability and Bioequivalence Studies for Nasal Aerosols and Nasal Sprays for Local Action. U.S. Department of Health and Human Services.” http://www.fda.gov/cder/guidance/index.htm.

[ptr70380-bib-0072] Food and Drug Administration . 2016. “Botanical Drug Development Guidance for Industry. U.S. Department of Health and Human Services.” http://www.fda.gov/Drugs/GuidanceComplianceRegulatoryInformation/Guidances/default.htm.

[ptr70380-bib-0073] Food and Drug Administration . 2024. “Questions and Answers on Current Good Manufacturing Practice Regulations|Production and Process Controls.” https://www.fda.gov/drugs/guidances‐drugs/questions‐and‐answers‐current‐good‐manufacturing‐practice‐regulations‐production‐and‐process?utm_s.

[ptr70380-bib-0074] Frey, W. H. , J. Liu , X. Chen , et al. 1997. “Delivery of 125I‐NGF to the Brain via the Olfactory Route Drug Delivery.” Journal of Delivery and Targeting of Therapeutic Agents 4, no. 2: 87–92. 10.3109/10717549709051878.

[ptr70380-bib-0075] Froelich, A. , T. Osmałek , B. Jadach , V. Puri , and B. Michniak‐Kohn . 2021. “Microemulsion‐Based Media in Nose‐To‐Brain Drug Delivery.” Pharmaceutics 13, no. 2: 1–37. 10.3390/pharmaceutics13020201. PMC7912993 33540856

[ptr70380-bib-0076] Gaba, B. , T. Khan , M. F. Haider , et al. 2019. “Vitamin E Loaded Naringenin Nanoemulsion via Intranasal Delivery for the Management of Oxidative Stress in a 6‐OHDA Parkinson's Disease Model.” BioMed Research International 2019: 1–20. 10.1155/2019/2382563. PMC6487130 31111044

[ptr70380-bib-0077] Gaggeri, R. , D. Rossi , M. S. Christodoulou , et al. 2012. “Chiral Flavanones From Amygdalus Lycioides Spach: Structural Elucidation and Identification of TNFalpha Inhibitors by Bioactivity‐Guided Fractionation.” Molecules 17, no. 2: 1665–1674. 10.3390/molecules17021665. 22318322 PMC6268923

[ptr70380-bib-0078] Galgatte, U. C. , A. B. Kumbhar , and P. D. Chaudhari . 2014. “Development of In Situ Gel for Nasal Delivery: Design, Optimization, In Vitro and In Vivo Evaluation.” Drug Delivery 21, no. 1: 62–73. 10.3109/10717544.2013.849778. 24191774

[ptr70380-bib-0079] Gavini, E. , G. Rassu , V. Sanna , M. Cossu , and P. Giunchedi . 2005. “Mucoadhesive Microspheres for Nasal Administration of an Antiemetic Drug, Metoclopramide: In‐Vitro/Ex‐Vivo Studies.” Journal of Pharmacy and Pharmacology 57, no. 3: 287–294. 10.1211/0022357055623. 15807983

[ptr70380-bib-0098] Generally Recognized as Safe . 2023. “Federal Register.” https://www.gpo.gov/fdsys/pkg/FR‐2010‐12‐28/pdf/2010‐32344.pdf.

[ptr70380-bib-0080] Gohlke, A. , C. J. Ingelmann , G. Nürnberg , A. Starke , S. Wolffram , and C. C. Metges . 2013. “Bioavailability of Quercetin From Its Aglycone and Its Glucorhamnoside Rutin in Lactating Dairy Cows After Intraduodenal Administration.” Journal of Dairy Science 96, no. 4: 2303–2313. 10.3168/jds.2012-6234. 23403185

[ptr70380-bib-0081] Gohlke, A. , C. J. Ingelmann , G. Nürnberg , et al. 2013. “Influence of 4‐Week Intraduodenal Supplementation of Quercetin on Performance, Glucose Metabolism, and mRNA Abundance of Genes Related to Glucose Metabolism and Antioxidative Status in Dairy Cows.” Journal of Dairy Science 96, no. 11: 6986–7000. 10.3168/jds.2013-6852. 24054306

[ptr70380-bib-0082] Gong, E. J. , H. R. Park , M. E. Kim , et al. 2011. “Morin Attenuates Tau Hyperphosphorylation by Inhibiting GSK3β.” Neurobiology of Disease 44, no. 2: 223–230. 10.1016/j.nbd.2011.07.005. 21782947 PMC3166962

[ptr70380-bib-0083] Gradolatto, A. , J. P. Basly , R. Berges , et al. 2005. “Pharmacokinetics and Metabolism of Apigenin in Female and Male Rats After a Single Oral Administration.” Drug Metabolism and Disposition 33, no. 1: 49–54. 10.1124/dmd.104.000893. 15466493

[ptr70380-bib-0084] Grassin‐Delyle, S. , A. Buenestado , E. Naline , et al. 2012. “Intranasal Drug Delivery: An Efficient and Non‐Invasive Route for Systemic Administration–Focus on Opioids.” Pharmacology and Therapeutics 134, no. 3: 366–379. 10.1016/j.pharmthera.2012.03.003. 22465159

[ptr70380-bib-0085] Guardia, T. , A. Ester Rotelli , A. Osvaldo Juarez , and L. Eugenia Pelzer . 2001. “Anti‐Inflammatory Properties of Plant Flavonoids. Effects of Rutin, Quercetin and Hesperidin on Adjuvant Arthritis in Rat.” Il Farmaco 56, no. 9: 683–687. https://www.elsevier.com/locate/farmac. 11680812 10.1016/s0014-827x(01)01111-9

[ptr70380-bib-0086] Guo, J. , E. X. Shang , J. A. Duan , Y. Tang , D. Qian , and S. Su . 2010. “Fast and Automated Characterization of Major Constituents in Rat Biofluid After Oral Administration of *Abelmoschus manihot* Extract Using Ultra‐Performance Liquid Chromatography/Quadrupole Time‐Of‐Flight Mass Spectrometry and Metabolynx.” Rapid Communications in Mass Spectrometry 24, no. 4: 443–453. 10.1002/rcm.4416. 20069688

[ptr70380-bib-0087] Gutiérrez‐Salmeán, G. , P. Ortiz‐Vilchis , C. M. Vacaseydel , et al. 2014. “Effects of (−)‐Epicatechin on a Diet‐Induced Rat Model of Cardiometabolic Risk Factors.” European Journal of Pharmacology 728, no. 1: 24–30. 10.1016/j.ejphar.2014.01.053. 24491839

[ptr70380-bib-0088] Gymnastiar, A. P. , D. S. Damayanti , and A. Tilaqza . 2024. “Molecular Docking of *Carica papaya* Leaves as Antihypertensive at ACE and Angiotensin II Receptor.” Research Journal of Pharmacy and Technology 17, no. 12: 5908–5914. 10.52711/0974-360X.2024.00896.

[ptr70380-bib-0089] Han, S. , Z. Wu , Y. Jin , W. Yang , and H. Shi . 2015. “RNA‐Seq Analysis for Transcriptome Assembly, Gene Identification, and SSR Mining in Ginkgo ( *Ginkgo biloba* L.).” Tree Genetics and Genomes 11, no. 3: 37. 10.1007/s11295-015-0868-8.

[ptr70380-bib-0090] Han, X. , X. He , X. Zhan , et al. 2023. “Disturbed Microbiota‐Metabolites‐Immune Interaction Network Is Associated With Olfactory Dysfunction in Patients With Chronic Rhinosinusitis.” Frontiers in Immunology 14: 1159112. 10.3389/fimmu.2023.1159112. 37292198 PMC10245275

[ptr70380-bib-0091] Hariri, B. M. , D. B. McMahon , B. Chen , et al. 2017. “Flavones Modulate Respiratory Epithelial Innate Immunity: Anti‐Inflammatory Effects and Activation of the T2R14 Receptor.” Journal of Biological Chemistry 292, no. 20: 8484–8497. 10.1074/jbc.M116.771949. 28373278 PMC5437252

[ptr70380-bib-0092] Hassimotto, N. M. A. , M. I. Genovese , and F. M. Lajolo . 2008. “Absorption and Metabolism of Cyanidin‐3‐Glucoside and Cyanidin‐3‐Rutinoside Extracted From Wild Mulberry ( *Morus nigra* L.) in Rats.” Nutrition Research 28, no. 3: 198–207. 10.1016/j.nutres.2007.12.012. 19083408

[ptr70380-bib-0093] Hatanaka, R. , A. Taguchi , Y. Nagao , et al. 2024. “The Flavonoid Sudachitin Regulates Glucose Metabolism via PDE Inhibition.” Heliyon 10, no. 16: 1–10. 10.1016/j.heliyon.2024.e35978. PMC11367099 39224336

[ptr70380-bib-0094] Hattori, M. , H. Mizuguchi , Y. Baba , et al. 2013. “Quercetin Inhibits Transcriptional Up‐Regulation of Histamine H1 Receptor via Suppressing Protein Kinase C‐δ/Extracellular Signal‐Regulated Kinase/Poly(ADP‐Ribose) Polymerase‐1 Signaling Pathway in HeLa Cells.” International Immunopharmacology 15, no. 2: 232–239. 10.1016/j.intimp.2012.12.030. 23333628

[ptr70380-bib-0095] Henriques, P. , A. Fortuna , and S. Doktorovová . 2022. “Spray Dried Powders for Nasal Delivery: Process and Formulation Considerations.” European Journal of Pharmaceutics and Biopharmaceutics 176: 1–20. 10.1016/j.ejpb.2022.05.002. 35568256

[ptr70380-bib-0096] Hiroyuki, S. , O. Takeshi , K. Akiharu , et al. 2009. “Distribution and Excretion of Bilberry Anthocyanines in Mice.” Journal of Agricultural and Food Chemistry 57, no. 17: 7681–7686. 10.1021/jf901341b. 19663426

[ptr70380-bib-0097] Hou, H. , Y. Li , Z. Xu , et al. 2023. “Applications and Research Progress of Traditional Chinese Medicine Delivered via Nasal Administration.” Biomedicine and Pharmacotherapy 157, no. 3: 113933. 10.1016/j.biopha.2022.113933. 36399826

[ptr70380-bib-0099] Hu, Q. , and L. Peng . 2023. “Diosmetin Alleviates Ovalbumin‐Induced Nasal Inflammation by Regulating the SIRT1/NF‐κB Signaling in Mouse Models of Allergic Rhinitis.” Revista Brasileira de Farmacognosia 33, no. 12: 1232–1242. 10.1007/s43450-023-00448-w.

[ptr70380-bib-0100] Huang, Q. , X. Chen , S. Yu , G. Gong , and H. Shu . 2023. “Research Progress in Brain‐Targeted Nasal Drug Delivery.” Frontiers in Aging Neuroscience 15: 1–12. 10.3389/fnagi.2023.1341295. PMC10828028 38298925

[ptr70380-bib-0101] Huanga, J. , R. Jib , X. Qianc , and Y. Shen . 2024. “Isoorientin Alleviates Ovalbumin‐Stimulated Allergic Rhinitis in Mice by Restoring Th1/Th2 Balance.” Allergologia et Immunopathologia 52, no. 5: 29–35. 39278848 10.15586/aei.v52i5.1154

[ptr70380-bib-0102] Hussain, A. , S. Azam , R. Maqsood , et al. 2024. “Chemistry, Biosynthesis, and Theranostics of Antioxidant Flavonoids and Polyphenolics of Genus Rhododendron: An Overview.” Naunyn‐Schmiedeberg's Archives of Pharmacology 398: 1–44. 10.1007/s00210-024-03428-6. 39276249

[ptr70380-bib-0103] Iannone, M. , F. Alberti , M. C. Braganò , X. de la Torre , F. Molaioni , and F. Botrè . 2021. “Influence of Synthetic Isoflavones on Selected Urinary Steroid Biomarkers: Relevance to Doping Control.” Steroids 174: 1–10. 10.1016/j.steroids.2021.108900. 34391799

[ptr70380-bib-0104] Iannone, M. , F. Botrè , S. Parenti , D. Jardines , and X. de la Torre . 2019. “An Investigation on the Metabolic Pathways of Synthetic Isoflavones by Gas Chromatography Coupled to High Accuracy Mass Spectrometry.” Rapid Communications in Mass Spectrometry 33, no. 19: 1485–1493. 10.1002/rcm.8490. 31132805

[ptr70380-bib-0105] Ichiyanagi, T. , M. M. Rahman , Y. Kashiwada , et al. 2004a. “Absorption and Metabolism of Delphinidin 3‐O‐β‐D‐Glucoside in Rats.” BioFactors 21, no. 1–4: 411–413. 15630238 10.1002/biof.552210181

[ptr70380-bib-0106] Ichiyanagi, T. , M. M. Rahman , Y. Kashiwada , et al. 2004b. “Absorption and Metabolism of Delphinidin 3‐O‐β‐D‐Glucopyranoside in Rats.” Free Radical Biology and Medicine 36, no. 7: 930–937. 10.1016/j.freeradbiomed.2004.01.005. 15019977

[ptr70380-bib-0107] Illek, B. , and H. Fischer . 1998. “Flavonoids Stimulate cl Conductance of Human Airway Epithelium In Vitro and In Vivo.” American Physiological Society 275: L902–L910. 10.1152/ajplung.1998.275.5.L902 9815107

[ptr70380-bib-0108] Illek, B. , L. Zhang , N. C. Lewis , et al. 1999. “Defective Function of the Cystic Fibrosis‐Causing Missense Mutation G551D Is Recovered by Genistein.” American Journal of Physiology 277, no. 4: C833–C839. 10516113 10.1152/ajpcell.1999.277.4.C833

[ptr70380-bib-0109] Illum, L. 2000. “Transport of Drugs From the Nasal Cavity to the Central Nervous System.” European Journal of Pharmaceutical Sciences 11: 1–18. https://www.elsevier.nl/locate/ejps. 10913748 10.1016/s0928-0987(00)00087-7

[ptr70380-bib-0110] Inoue, T. , Y. Sugimoto , H. Masuda , and C. Kamei . 2002. “Antiallergic Effect of Flavonoid Glycosides Obtained From Mentha Piperita L.” Biological and Pharmaceutical Bulletin 25, no. 2: 256–259. 11853178 10.1248/bpb.25.256

[ptr70380-bib-0111] Jeong Choi, E. 2006. “The Prooxidant, Rather Than Antioxidant, Acts of Daidzein In Vivo and In Vitro: Daidzein Suppresses Glutathione Metabolism.” European Journal of Pharmacology 542, no. 1–3: 162–169. 10.1016/j.ejphar.2006.05.020. 16797001

[ptr70380-bib-0112] Jeong, S. H. , J. H. Jang , and Y. B. Lee . 2023. “Drug Delivery to the Brain via the Nasal Route of Administration: Exploration of Key Targets and Major Consideration Factors.” Journal of Pharmaceutical Investigation 53, no. 1: 119–152. 10.1007/s40005-022-00589-5. 35910081 PMC9308891

[ptr70380-bib-0113] Jin, M. J. , U. Kim , I. S. Kim , et al. 2010. “Effects of Gut Microflora on Pharmacokinetics of Hesperidin: A Study on Non‐Antibiotic and Pseudo‐Germ‐Free Rats.” Journal of Toxicology and Environmental Health, Part A 73, no. 21–22: 1441–1450. 10.1080/15287394.2010.511549. 20954071

[ptr70380-bib-0114] Kanaze, F. I. , M. I. Bounartzi , M. Georgarakis , and I. Niopas . 2007. “Pharmacokinetics of the Citrus Flavanone Aglycones Hesperetin and Naringenin After Single Oral Administration in Human Subjects.” European Journal of Clinical Nutrition 61, no. 4: 472–477. 10.1038/sj.ejcn.1602543. 17047689

[ptr70380-bib-0115] Kashiwabara, M. , K. Asano , T. Mizuyoshi , and H. Kobayashi . 2016. “Suppression of Neuropeptide Production by Quercetin in Allergic Rhinitis Model Rats.” BMC Complementary and Alternative Medicine 16, no. 1: 1–9. 10.1186/s12906-016-1123-z. 27207147 PMC4875744

[ptr70380-bib-0116] Keller, L. A. , O. Merkel , and A. Popp . 2022. “Intranasal Drug Delivery: Opportunities and Toxicologic Challenges During Drug Development.” Drug Delivery and Translational Research 12, no. 4: 735–757. 10.1007/s13346-020-00891-5. 33491126 PMC7829061

[ptr70380-bib-0117] Khoo, H. E. , A. Azlan , S. T. Tang , and S. M. Lim . 2017. “Anthocyanidins and Anthocyanins: Colored Pigments as Food, Pharmaceutical Ingredients, and the Potential Health Benefits.” Food and Nutrition Research 61: 1–21. 10.1080/16546628.2017.1361779. PMC5613902 28970777

[ptr70380-bib-0118] Khute, S. , and R. K. Jangde . 2023. “Optimization of Nasal Liposome Formulation of Venlafaxine Hydrochloride Using a Box‐Behnken Experimental Design.” Current Therapeutic Research, Clinical and Experimental 99: 1–13. 10.1016/j.curtheres.2023.100714. PMC10506098 37727460

[ptr70380-bib-0119] Kim, H. J. , J. H. Woo , Y. R. Nam , et al. 2020. “Luteolin Reduces Fluid Hypersecretion by Inhibiting TMEM16A in Interleukin‐4 Treated Calu‐3 Airway Epithelial Cells.” Korean Journal of Physiology and Pharmacology 24, no. 4: 329–338. 10.4196/KJPP.2020.24.4.329. 32587127 PMC7317179

[ptr70380-bib-0120] Koch, E. V. , S. Bendas , K. Nehlsen , T. May , S. Reichl , and A. Dietzel . 2023. “The Path From Nasal Tissue to Nasal Mucosa on Chip: Part 2—Advanced Microfluidic Nasal In Vitro Model for Drug Absorption Testing.” Pharmaceutics 15, no. 10: 1–17. 10.3390/pharmaceutics15102439. PMC10610000 37896199

[ptr70380-bib-0121] Kokkinis, S. , M. Singh , K. R. Paudel , et al. 2024. “Plant‐Based Therapeutics for Chronic Obstructive Pulmonary Diseases: Nanoformulation Strategies to Overcome Delivery Challenges.” Food Bioscience 58, no. 4: 103761. 10.1016/j.fbio.2024.103761.

[ptr70380-bib-0122] Kolanjiyil, A. V. , C. Kleinstreuer , N. C. Kleinstreuer , W. Pham , and R. T. Sadikot . 2019. “Mice‐To‐Men Comparison of Inhaled Drug‐Aerosol Deposition and Clearance.” Respiratory Physiology and Neurobiology 260: 82–94. 10.1016/j.resp.2018.11.003. 30445230

[ptr70380-bib-0123] Kopustinskiene, D. M. , V. Jakstas , A. Savickas , and J. Bernatoniene . 2020. “Flavonoids as Anticancer Agents.” Nutrients 12, no. 457: 1–25. 10.3390/nu12020457 PMC7071196 32059369

[ptr70380-bib-0124] Krasieva, T. B. , J. Ehren , T. O'Sullivan , B. J. Tromberg , and P. Maher . 2015. “Cell and Brain Tissue Imaging of the Flavonoid Fisetin Using Label‐Free Two‐Photon Microscopy.” Neurochemistry International 89: 243–248. 10.1016/j.neuint.2015.08.003. 26271433 PMC4587296

[ptr70380-bib-0125] Kreft, M. E. , U. D. Jerman , E. Lasič , et al. 2015. “The Characterization of the Human Nasal Epithelial Cell Line RPMI 2650 Under Different Culture Conditions and Their Optimization for an Appropriate In Vitro Nasal Model.” Pharmaceutical Research 32, no. 2: 665–679. 10.1007/s11095-014-1494-0. 25145337

[ptr70380-bib-0126] Ku, Y. S. , M. S. Ng , S. S. Cheng , et al. 2020. “Understanding the Composition, Biosynthesis, Accumulation and Transport of Flavonoids in Crops for the Promotion of Crops as Healthy Sources of Flavonoids for Human Consumption.” Nutrients 12, no. 6: 1–23. 10.3390/nu12061717. PMC7352743 32521660

[ptr70380-bib-0127] Lagoa, R. , J. Silva , J. R. Rodrigues , and A. Bishayee . 2020. “Advances in Phytochemical Delivery Systems for Improved Anticancer Activity.” Biotechnology Advances 38, no. 4: 107382. 10.1016/j.biotechadv.2019.04.004. 30978386

[ptr70380-bib-0128] Lai, M.‐Y. , S.‐L. Hsiu , S.‐Y. Tsai , Y.‐C. Hou , and P.‐D. L. Chao . 2010. “Comparison of Metabolic Pharmacokinetics of Baicalin and Baicalein in Rats.” Journal of Pharmacy and Pharmacology 55, no. 2: 205–209. 10.1211/002235702522. 12631413

[ptr70380-bib-0129] Lemon, K. P. 2020. “Human Nasal Microbiota.” Current Biology 30, no. 19: R1118–R1119. 33022252 10.1016/j.cub.2020.08.010

[ptr70380-bib-0130] Lewis, J. A. , B. Zhang , R. Harza , et al. 2023. “Structural Similarities and Overlapping Activities Among Dihydroflavonol 4‐Reductase, Flavanone 4‐Reductase, and Anthocyanidin Reductase Offer Metabolic Flexibility in the Flavonoid Pathway.” International Journal of Molecular Sciences 24, no. 18: 13901. 10.3390/ijms241813901. 37762209 PMC10531346

[ptr70380-bib-0131] Li, C. , L. Zhang , G. Lin , and Z. Zuo . 2011. “Identification and Quantification of Baicalein, Wogonin, Oroxylin A and Their Major Glucuronide Conjugated Metabolites in Rat Plasma After Oral Administration of Radix Scutellariae Product.” Journal of Pharmaceutical and Biomedical Analysis 54, no. 4: 750–758. 10.1016/j.jpba.2010.10.005. 21051171

[ptr70380-bib-0132] Li, C. R. , L. Zhang , S. K. Wo , L. M. Zhou , G. Lin , and Z. Zuo . 2012. “Pharmacokinetic Interactions Among Major Bioactive Components in Radix Scutellariae via Metabolic Competition.” Biopharmaceutics and Drug Disposition 33, no. 9: 487–500. 10.1002/bdd.1815. 22933367

[ptr70380-bib-0133] Li, H. , X. Shen , B. Zhang , et al. 2025. “Brain‐Targeted Intranasal Delivery of Biologics: A Perspective for Alzheimer's Disease Treatment.” RSC Pharmaceutics 2: 1323–1348. 10.1039/d5pm00148j.

[ptr70380-bib-0134] Li, H. , H. Zhang , and H. Zhao . 2023. “Apigenin Attenuates Inflammatory Response in Allergic Rhinitis Mice by Inhibiting the TLR4/MyD88/NF‐κB Signaling Pathway.” Environmental Toxicology 38, no. 2: 253–265. 10.1002/tox.23699. 36350155

[ptr70380-bib-0135] Li, M. M. , R. Y. Peng , W. J. Wang , et al. 2023. “Interaction With Taxifolin Reduces the Digestibility of Corn Starch In Vitro and In Vivo.” Journal of Food Measurement and Characterization 17, no. 4: 4026–4033. 10.1007/s11694-023-01930-8.

[ptr70380-bib-0136] Li, N. , Y. J. Ye , M. Yang , X. H. Jiang , and J. H. Ma . 2011. “Pharmacokinetics of Baicalin‐Phospholipid Complex in Rat Plasma and Brain Tissues After Intranasal and Intravenous Administration.” Pharmazie 66, no. 5: 374–377. 10.1691/ph.2011.0783. 21699072

[ptr70380-bib-0137] Li, Y. , C. Guang , N. Zhao , X. Feng , and F. Qiu . 2019. “LC‐MS/MS Method for Simultaneous Determination of Linarin and Its Metabolites in Rat Plasma and Liver Tissue Samples: Application to Pharmacokinetic and Liver Tissue Distribution Study After Oral Administration of Linarin.” Molecules 24, no. 18: 1–12. 10.3390/molecules24183342. PMC6766828 31540332

[ptr70380-bib-0138] Li, Y. , H. Su , W. Wang , et al. 2023. “Fabrication of Taxifolin Loaded Zein‐Caseinate Nanoparticles and Its Bioavailability in Rat.” Food Science and Human Wellness 12, no. 6: 2306–2313. 10.1016/j.fshw.2023.03.034.

[ptr70380-bib-0139] Liang, K. , A. D. Kandhare , A. A. Mukherjee‐Kandhare , S. L. Bodhankar , and D. Xu . 2019. “Morin Ameliorates Ovalbumin‐Induced Allergic Rhinitis via Inhibition of STAT6/SOCS1 and GATA3/T‐Bet Signaling Pathway in BALB/c Mice.” Journal of Functional Foods 55: 391–401. 10.1016/j.jff.2019.01.052.

[ptr70380-bib-0140] Lim, S. H. , M. K. You , D. H. Kim , J. K. Kim , J. Y. Lee , and S. H. Ha . 2016. “RNAi‐Mediated Suppression of Dihydroflavonol 4‐Reductase in Tobacco Allows Fine‐Tuning of Flower Color and Flux Through the Flavonoid Biosynthetic Pathway.” Plant Physiology and Biochemistry 109: 482–490. 10.1016/j.plaphy.2016.10.028. 27842297

[ptr70380-bib-0141] Liu, H. , G. Chang , W. Wang , Z. Ji , J. Cui , and Y. Peng . 2022. “Pharmacokinetics, Prostate Distribution and Metabolic Characteristics of Four Representative Flavones After Oral Administration of the Aerial Part of Glycyrrhiza Uralensis in Rats.” Molecules 27, no. 10: 1–29. 10.3390/molecules27103245. PMC9144537 35630722

[ptr70380-bib-0142] Liu, M. , C. Yang , W. Zou , et al. 2011. “Toxicokinetics of Naringin, a Putative Antitussive, After 184‐Day Repeated Oral Administration in Rats.” Environmental Toxicology and Pharmacology 31, no. 3: 485–489. 10.1016/j.etap.2011.01.006. 21787720

[ptr70380-bib-0143] Liu, M. , W. Zou , C. Yang , W. Peng , and W. Su . 2012. “Metabolism and Excretion Studies of Oral Administered Naringin, a Putative Antitussive, in Rats and Dogs.” Biopharmaceutics and Drug Disposition 33, no. 3: 123–134. 10.1002/bdd.1775. 22374702

[ptr70380-bib-0144] Liu, Y. , X. Shi , Y. Tian , et al. 2023. “An Insight Into Novel Therapeutic Potentials of Taxifolin.” Frontiers in Pharmacology 14: 1–17. 10.3389/fphar.2023.1173855. PMC10227600 37261284

[ptr70380-bib-0145] Loke, W. M. , J. M. Hodgson , J. M. Proudfoot , A. J. Mckinley , I. B. Puddey , and K. D. Croft . 2008. “Pure Dietary Flavonoids Quercetin and (−)‐Epicatechin Augment Nitric Oxide Products and Reduce Endothelin‐1 Acutely in Healthy Men 1‐3.” American Journal of Clinical Nutrition 88, no. 4: 1018–1043. 18842789 10.1093/ajcn/88.4.1018

[ptr70380-bib-0146] López Luengo, M. T. 2002. “Flavonoides Concepto y Clasificacion.” Fitoterapia 21, no. 4: 108–113.

[ptr70380-bib-0147] Lou, Q. , J. Wen , Y. Jiang , J. Huang , G. Fan , and F. Song . 2018. “Rapid Determination of 3′ 4′‐Dimethoxy Flavonol‐3‐β‐D‐Glucopyranoside in Rat Plasma by LC‐MS/MS Method Followed by Protein Precipitation.” Journal of Chromatography B: Analytical Technologies in the Biomedical and Life Sciences 1086: 47–55. 10.1016/j.jchromb.2018.04.008. 29656083

[ptr70380-bib-0148] Lowe, S. , E. Sher , G. Wishart , et al. 2017. “An Assessment of the Central Disposition of Intranasally Administered Insulin Lispro in the Cerebrospinal Fluid of Healthy Volunteers and Beagle Dogs.” Drug Delivery and Translational Research 7, no. 1: 11–15. 10.1007/s13346-016-0325-8. 27553192

[ptr70380-bib-0149] Luo, C. F. , M. Yuan , M. S. Chen , S. M. Liu , and H. Ji . 2010. “Metabolites of Puerarin Identified by Liquid Chromatography Tandem Mass Spectrometry: Similar Metabolic Profiles in Liver and Intestine of Rats.” Journal of Chromatography B: Analytical Technologies in the Biomedical and Life Sciences 878, no. 3–4: 363–370. 10.1016/j.jchromb.2009.12.002. 20006564

[ptr70380-bib-0150] Luo, L. Y. , M. X. Fan , H. Y. Zhao , M. X. Li , X. Wu , and W. Y. Gao . 2018. “Pharmacokinetics and Bioavailability of the Isoflavones Formononetin and Ononin and Their In Vitro Absorption in Ussing Chamber and Caco‐2 Cell Models.” Journal of Agricultural and Food Chemistry 66, no. 11: 2917–2924. 10.1021/acs.jafc.8b00035. 29504397

[ptr70380-bib-0152] Ma, Q. , G. Li , J. He , J. Wang , and B. Ye . 2023. “Hydroxysafflor Yellow A Attenuates Allergic Response of Ovalbumin Induced Allergic Rhinitis via Nrf2/HO‐1 and Inflammatory Signaling Pathways.” Environmental Toxicology 38, no. 7: 1520–1534. 10.1002/tox.23783. 37195255

[ptr70380-bib-0153] Maciej, J. , C. T. Schäff , E. Kanitz , et al. 2015. “Bioavailability of the Flavonol Quercetin in Neonatal Calves After Oral Administration of Quercetin Aglycone or Rutin.” Journal of Dairy Science 98, no. 6: 3906–3917. 10.3168/jds.2015-9361. 25795488 PMC7094564

[ptr70380-bib-0154] Mainardes, R. M. , M. C. Cocenza Urban , P. Oliveira Cinto , et al. 2006. “Liposomes and Micro/Nanoparticles as Colloidal Carriers for Nasal Drug Delivery.” Current Drug Delivery 3, no. 3: 275–285. 16848729 10.2174/156720106777731019

[ptr70380-bib-0155] Majdoub, Y. O. E. , M. Diouri , P. Arena , et al. 2019. “Evaluation of the Availability of Delphinidin and Cyanidin‐3‐O‐Sambubioside From Hibiscus Sabdariffa and 6‐Gingerol From *Zingiber officinale* in Colon Using Liquid Chromatography and Mass Spectrometry Detection.” European Food Research and Technology 245, no. 11: 2425–2433. 10.1007/s00217-019-03358-1.

[ptr70380-bib-0156] Mall, M. , A. Wissner , H. H. Seydewitz , et al. 2000. “Effect of Genistein on Native Epithelial Tissue From Normal Individuals and CF Patients and on Ion Channels Expressed in Xenopus Oocytes 1,2.” British Journal of Pharmacology 130, no. 8: 1884–1892. https://www.nature.com/bjp. 10952679 10.1038/sj.bjp.0703520 PMC1572276

[ptr70380-bib-0157] Mallants, R. , M. Jorissen , and P. Augustijns . 2007. “Effect of Preservatives on Ciliary Beat Frequency in Human Nasal Epithelial Cell Culture: Single Versus Multiple Exposure.” International Journal of Pharmaceutics 338, no. 1–2: 64–69. 10.1016/j.ijpharm.2007.01.029. 17324538

[ptr70380-bib-0158] Manta, K. , P. Papakyriakopoulou , M. Chountoulesi , et al. 2020. “Preparation and Biophysical Characterization of Quercetin Inclusion Complexes With β‐Cyclodextrin Derivatives to Be Formulated as Possible Nose‐To‐Brain Quercetin Delivery Systems.” Molecular Pharmaceutics 17, no. 11: 4241–4255. 10.1021/acs.molpharmaceut.0c00672. 32986435

[ptr70380-bib-0159] Manta, K. , P. Papakyriakopoulou , A. Nikolidaki , et al. 2023. “Comparative Serum and Brain Pharmacokinetics of Quercetin After Oral and Nasal Administration to Rats as Lyophilized Complexes With β‐Cyclodextrin Derivatives and Their Blends With Mannitol/Lecithin Microparticles.” Pharmaceutics 15: 2036. 10.3390/pharmaceutics15082036. 37631250 PMC10459069

[ptr70380-bib-0160] Manthey, J. A. , P. S. Ferreira , and T. B. Cesar . 2022. “Influences of Solubility and Vehicle Carriers on Eriodictyol Pharmacokinetics in Rats.” Journal of Agricultural and Food Chemistry 70, no. 15: 4667–4676. 10.1021/acs.jafc.2c00319. 35394285

[ptr70380-bib-0161] Marcello, E. , and V. Chiono . 2023. “Biomaterials‐Enhanced Intranasal Delivery of Drugs as a Direct Route for Brain Targeting.” International Journal of Molecular Sciences 24, no. 4: 1–27. 10.3390/ijms24043390. PMC9964911 36834804

[ptr70380-bib-0162] Marczylo, T. H. , D. Cooke , K. Brown , W. P. Steward , and A. J. Gescher . 2009. “Pharmacokinetics and Metabolism of the Putative Cancer Chemopreventive Agent Cyanidin‐3‐Glucoside in Mice.” Cancer Chemotherapy and Pharmacology 64, no. 6: 1261–1268. 10.1007/s00280-009-0996-7. 19363608

[ptr70380-bib-0163] Maronpot, R. R. , M. Koyanagi , J. Davis , et al. 2015. “Safety Assessment and Single‐Dose Toxicokinetics of the Flavouring Agent Myricitrin in Sprague–Dawley Rats.” Food Additives and Contaminants. Part A 32, no. 11: 1799–1809. 10.1080/19440049.2015.1084653. 26365525

[ptr70380-bib-0164] Md, S. , N. A. Alhakamy , H. M. Aldawsari , and H. Z. Asfour . 2019. “Neuroprotective and Antioxidant Effect of Naringenin‐Loaded Nanoparticles for Nose‐To‐Brain Delivery.” Brain Sciences 9: 275. 10.3390/brainsci9100275. 31618942 PMC6827151

[ptr70380-bib-0165] Meng, J. , P. Zhou , Y. Liu , et al. 2013. “The Development of Nasal Polyp Disease Involves Early Nasal Mucosal Inflammation and Remodelling.” PLoS One 8, no. 12: 1–13. 10.1371/journal.pone.0082373. PMC3858290 24340021

[ptr70380-bib-0166] Merkus, F. W. H. M. , J. C. Verhoef , E. Marttin , et al. 1999. “Cyclodextrins in Nasal Drug Delivery.” Advanced Drug Delivery Reviews 36, no. 1: 41–57. 10837708 10.1016/s0169-409x(98)00054-4

[ptr70380-bib-0167] Moon, Y. J. , K. Sagawa , K. Frederick , S. Zhang , and M. E. Morris . 2006. “Pharmacokinetics and Bioavailability of the Isoflavone Biochanin A in Rats.” AAPS Journal 8, no. 51: E433–E442. 17025260 10.1208/aapsj080351 PMC2761049

[ptr70380-bib-0168] Moon, Y. J. , B. S. Shin , G. An , and M. E. Morris . 2008. “Biochanin A Inhibits Breast Cancer Tumor Growth in a Murine Xenograft Model.” Pharmaceutical Research 25, no. 9: 2158–2163. 10.1007/s11095-008-9583-6. 18454305

[ptr70380-bib-0169] Motoichi, K. , Y. Nunnarpas , J. Y. Ying , et al. 2015. “Micellar Nanocomplex (Patent ES2847602T3).”

[ptr70380-bib-0170] Mu, D. , L. Zhou , L. Shi , et al. 2024. “Quercetin‐Crosslinked Chitosan Nanoparticles: A Potential Treatment for Allergic Rhinitis.” Scientific Reports 14, no. 1: 1–13. 10.1038/s41598-024-54501-2. 38369554 PMC10874938

[ptr70380-bib-0171] Murota, K. , R. Cermak , J. Terao , and S. Wolffram . 2013. “Influence of Fatty Acid Patterns on the Intestinal Absorption Pathway of Quercetin in Thoracic Lymph Duct‐Cannulated Rats.” British Journal of Nutrition 109, no. 12: 2147–2153. 10.1017/S0007114512004564. 23200408

[ptr70380-bib-0172] Nagaraja, S. , G. M. Basavarajappa , R. K. Karnati , E. M. Bakir , and S. Pund . 2021. “Ion‐Triggered In Situ Gelling Nanoemulgel as a Platform for Nose‐To‐Brain Delivery of Small Lipophilic Molecules.” Pharmaceutics 13: 1216. 10.3390/pharmaceutics13081216. 34452177 PMC8400950

[ptr70380-bib-0173] Najafian, M. , M. Z. Jahromi , M. J. Nowroznejhad , et al. 2012. “Phloridzin Reduces Blood Glucose Levels and Improves Lipids Metabolism in Streptozotocin‐Induced Diabetic Rats.” Molecular Biology Reports 39, no. 5: 5299–5306. 10.1007/s11033-011-1328-7. 22167331

[ptr70380-bib-0174] Nectoux, A. M. , C. Abe , S. W. Huang , et al. 2019. “Absorption and Metabolic Behavior of Hesperidin (Rutinosylated Hesperetin) After Single Oral Administration to Sprague‐Dawley Rats.” Journal of Agricultural and Food Chemistry 67, no. 35: 9812–9819. 10.1021/acs.jafc.9b03594. 31392887

[ptr70380-bib-0175] Nguyen, M. A. , P. Staubach , S. Wolffram , and P. Langguth . 2015. “The Influence of Single‐Dose and Short‐Term Administration of Quercetin on the Pharmacokinetics of Midazolam in Humans.” Journal of Pharmaceutical Sciences 104, no. 9: 3199–3207. 10.1002/jps.24500. 25988261

[ptr70380-bib-0176] Nielsen, S. E. , V. Breinholt , C. Cornett , and L. O. Dragsted . 2000. “Biotransformation of the Citrus Flavone Tangeretin in Rats. Identication of Metabolites With Intact Flavane Nucleus.” Food and Chemical Toxicology 38, no. 9: 739–746. https://www.elsevier.com/locate/foodchemtox. 10930694 10.1016/s0278-6915(00)00072-7

[ptr70380-bib-0177] Okolo, E. N. , D. I. Ugwu , B. E. Ezema , et al. 2021. “New Chalcone Derivatives as Potential Antimicrobial and Antioxidant Agent.” Scientific Reports 11, no. 1: 1–13. 10.1038/s41598-021-01292-5. 34741131 PMC8571407

[ptr70380-bib-0178] Padrah, S. , and M. Vajdy . 2010. “Adjuvant Compositions and Methods of Use (Patent AU2010203223B2).”

[ptr70380-bib-0179] Panche, A. N. , A. D. Diwan , and S. R. Chandra . 2016. “Flavonoids: An Overview.” Journal of Nutritional Science 5, no. e47: 1–15. 10.1017/jns.2016.41 PMC5465813 28620474

[ptr70380-bib-0180] Pandit, R. , L. Chen , and J. Götz . 2020. “The Blood‐Brain Barrier: Physiology and Strategies for Drug Delivery.” Advanced Drug Delivery Reviews 165–166: 1–14. 10.1016/j.addr.2019.11.009. 31790711

[ptr70380-bib-0181] Papakyriakopoulou, P. , K. Manta , C. Kostantini , et al. 2021. “Nasal Powders of Quercetin‐β‐Cyclodextrin Derivatives Complexes With Mannitol/Lecithin Microparticles for Nose‐To‐Brain Delivery: In Vitro and Ex Vivo Evaluation.” International Journal of Pharmaceutics 607, no. 12: 121016. 10.1016/j.ijpharm.2021.121016. 34411652

[ptr70380-bib-0182] Passamonti, S. , A. Vanzo , U. Vrhovsek , et al. 2005. “Hepatic Uptake of Grape Anthocyanins and the Role of Bilitranslocase.” Food Research International 38, no. 8–9: 953–960. 10.1016/j.foodres.2005.02.015.

[ptr70380-bib-0183] Pesic, M. , F. Schippers , R. Saunders , L. Webster , M. Donsbach , and T. Stoehr . 2020. “Pharmacokinetics and Pharmacodynamics of Intranasal Remimazolam‐A Randomized Controlled Clinical Trial.” European Journal of Clinical Pharmacology 76: 1505–1516. 10.1007/s00228-020-02984-z/Published. 32886178 PMC7557484

[ptr70380-bib-0184] Podder, B. , K. S. Song , H.‐Y. Song , and Y.‐S. Kim . 2014. “Cytoprotective Effect of Kaempferol on Paraquat‐Exposed BEAS‐2B Cells via Modulating Expression of MUC5AC.” Biological and Pharmaceutical Bulletin 37, no. 9: 1486–1494. 25177032 10.1248/bpb.b14-00239

[ptr70380-bib-0185] Pourhajibagher, M. , and A. Bahador . 2024. “Bioinformatics Analysis of Photoexcited Natural Flavonoid Glycosides as the Inhibitors for Oropharyngeal HPV Oncoproteins.” AMB Express 14, no. 1: 1–22. 10.1186/s13568-024-01684-6. 38466452 PMC10928025

[ptr70380-bib-0186] Preeti , S. Sambhakar , R. Malik , et al. 2023. “Nanoemulsion: An Emerging Novel Technology for Improving the Bioavailability of Drugs.” Scientifica 2023: 1–25. 10.1155/2023/6640103 PMC10625491 37928749

[ptr70380-bib-0187] Pyle, L. C. , J. C. Fulton , P. A. Sloane , et al. 2010. “Activation of the Cystic Fibrosis Transmembrane Conductance Regulator by the Flavonoid Quercetin Potential Use as a Biomarker of DF508 Cystic Fibrosis Transmembrane Conductance Regulator Rescue.” American Journal of Respiratory Cell and Molecular Biology 43, no. 5: 607–616. https://www.atsjournals.org. 20042712 10.1165/rcmb.2009-0281OC PMC2970857

[ptr70380-bib-0188] Qian, L. , M. T. Cook , and C. A. Dreiss . 2025. “In Situ Gels for Nasal Delivery: Formulation, Characterization and Applications.” Macromolecular Materials and Engineering 310, no. 6: 1–25. 10.1002/mame.202400356.

[ptr70380-bib-0189] Qiu, H. , X. Duan , Y. Su , S. Zeng , D. Zheng , and W. Liao . 2025. “Progress in Nasal Drug Delivery Research Based on Biomaterials.” APL Materials 13, no. 10: 1–27. 10.1063/5.0296863.

[ptr70380-bib-0190] Ramesh, P. , R. Jagadeesan , S. Sekaran , A. Dhanasekaran , and S. Vimalraj . 2021. “Flavonoids: Classification, Function, and Molecular Mechanisms Involved in Bone Remodelling.” Frontiers in Endocrinology 12: 1–22. 10.3389/fendo.2021.779638. PMC8649804 34887836

[ptr70380-bib-0191] Ranjbar, S. , A. Emamjomeh , F. Sharifi , et al. 2023. “Lipid‐Based Delivery Systems for Flavonoids and Flavonolignans: Liposomes, Nanoemulsions, and Solid Lipid Nanoparticles.” Pharmaceutics 15, no. 7: 1–25. 10.3390/pharmaceutics15071944. PMC10383758 37514130

[ptr70380-bib-0192] Rassu, G. , M. Sorrenti , L. Catenacci , et al. 2021. “Versatile Nasal Application of Cyclodextrins: Excipients and/or Actives?” Pharmaceutics 13, no. 8: 1180. 10.3390/pharmaceutics13081180. 34452141 PMC8401481

[ptr70380-bib-0193] Reiter, M. , K. Rupp , P. Baumeister , S. Zieger , and U. Harréus . 2009. “Antioxidant Effects of Quercetin and Coenzyme Q10 in Mini Organ Cultures of Human Nasal Mucosa Cells.” Anticancer Research 29, no. 1: 33–40. 19331131

[ptr70380-bib-0194] Rothwell, J. A. , A. J. Day , and M. R. A. Morgan . 2005. “Experimental Determination of Octanol‐Water Partition Coefficients of Quercetin and Related Flavonoids.” Journal of Agricultural and Food Chemistry 53, no. 11: 4355–4360. 10.1021/jf0483669. 15913295

[ptr70380-bib-0195] Saad, S. E. A. , D. J. L. Jones , L. M. Norris , et al. 2012. “Tissue Distribution and Metabolism of the Putative Cancer Chemopreventive Agent 3′,4′,5′‐Trimethoxyflavonol (TMFol) in Mice.” Biomedical Chromatography 26, no. 12: 1559–1566. 10.1002/bmc.2732. 22454297

[ptr70380-bib-0196] Saitani, E. M. , P. Papakyriakopoulou , T. Bogri , et al. 2025. “Cyclodextrin‐Based Quercetin Powders for Potential Nose‐To‐Brain Transport: Formulation and In Vitro Assessment.” Molecules 30, no. 13: 1–22. 10.3390/molecules30132878. PMC12251352 40649391

[ptr70380-bib-0197] Sarawek, S. , B. Feistel , I. Pischel , and V. Butterweck . 2008. “Flavonoids of *Cynara scolymus* Possess Potent Xanthinoxidase Inhibitory Activity In Vitro but Are Devoid of Hypouricemic Effects in Rats After Oral Application.” Planta Medica 74, no. 3: 221–227. 10.1055/s-2008-1034316. 18300193

[ptr70380-bib-0198] Schleimer, R. P. 2017. “Immunopathogenesis of Chronic Rhinosinusitis and Nasal Polyposis.” Annual Review of Pathology: Mechanisms of Disease 12, no. 1: 331–357. 10.1146/annurev-pathol-052016-100401. PMC5514544 27959637

[ptr70380-bib-0199] Seifelnasr, A. , X. A. Si , and J. Xi . 2023. “Visualization and Estimation of Nasal Spray Delivery to Olfactory Mucosa in an Image‐Based Transparent Nasal Model.” Pharmaceutics 15, no. 6: 1–21. 10.3390/pharmaceutics15061657. PMC10303528 37376105

[ptr70380-bib-0200] Selvaraj, K. , K. Gowthamarajan , and V. V. S. R. Karri . 2018. “Nose to Brain Transport Pathways an Overview: Potential of Nanostructured Lipid Carriers in Nose to Brain Targeting.” Artificial Cells, Nanomedicine, and Biotechnology 46, no. 8: 2088–2095. 10.1080/21691401.2017.1420073. 29282995

[ptr70380-bib-0201] Shen, N. , T. Wang , Q. Gan , S. Liu , L. Wang , and B. Jin . 2022. “Plant Flavonoids: Classification, Distribution, Biosynthesis, and Antioxidant Activity.” Food Chemistry 383: 132531. 10.1016/j.foodchem.2022.132531. 35413752

[ptr70380-bib-0202] Shi, L. L. , P. Xiong , L. Zhang , et al. 2013. “Features of Airway Remodeling in Different Types of Chinese Chronic Rhinosinusitis Are Associated With Inflammation Patterns.” Allergy: European Journal of Allergy and Clinical Immunology 68, no. 1: 101–109. 10.1111/all.12064. 23157215

[ptr70380-bib-0203] Shia, C. S. , S. Y. Tsai , S. C. Kuo , Y. C. Hou , and P. D. L. Chao . 2009. “Metabolism and Pharmacokinetics of 3,3′,4′,7‐ Tetrahydroxyflavone (Fisetin), 5‐Hydroxyflavone, and 7‐Hydroxyflavone and Antihemolysis Effects of Fisetin and Its Serum Metabolites.” Journal of Agricultural and Food Chemistry 57, no. 1: 83–89. 10.1021/jf802378q. 19090755

[ptr70380-bib-0204] Shrewsbury, S. B. 2023. “The Upper Nasal Space: Option for Systemic Drug Delivery, Mucosal Vaccines and Nose‐To‐Brain.” Pharmaceutics 15, no. 6: 1720. 10.3390/pharmaceutics15061720. 37376168 PMC10303426

[ptr70380-bib-0205] Singh, S. P. , Wahajuddin , M. M. Ali , and G. K. Jain . 2010. “High‐Throughput Quantification of Isoflavones, Biochanin A and Genistein, and Their Conjugates in Female Rat Plasma Using LC‐ESI‐MS/MS: Application in Pharmacokinetic Study.” Journal of Separation Science 33, no. 21: 3326–3334. 10.1002/jssc.201000456. 21049521

[ptr70380-bib-0206] Singh, S. P. , Wahajuddin , K. S. R. Raju , M. M. Ali , K. Kohli , and G. K. Jain . 2012. “Reduced Bioavailability of Tamoxifen and Its Metabolite 4‐Hydroxytamoxifen After Oral Administration With Biochanin A (An Isoflavone) in Rats.” Phytotherapy Research 26, no. 2: 303–307. 10.1002/ptr.3652. 22131128

[ptr70380-bib-0207] Sonawane, D. , and V. Pokharkar . 2024. “Quercetin‐Loaded Nanostructured Lipid Carrier In Situ Gel for Brain Targeting Through Intranasal Route: Formulation, In Vivo Pharmacokinetic and Pharmacodynamic Studies.” AAPS PharmSciTech 25, no. 2: 1–25. 10.1208/s12249-024-02736-7. 38316672

[ptr70380-bib-0208] Sosic‐Jurjevic, B. , B. Filipović , V. Ajdžanović , et al. 2007. “Subcutaneously Administrated Genistein and Daidzein Decrease Serum Cholesterol and Increase Triglyceride Levels in Male Middle‐Aged Rats.” Experimental Biology and Medicine 232, no. 9: 1222–1227. 10.3181/0703-BC-82. 17895530

[ptr70380-bib-0209] Souza, F. R. , F. Fornasier , L. M. P. Souza , et al. 2020. “Interaction of Naringin and Naringenin With DPPC Monolayer at the Air‐Water Interface.” Colloids and Surfaces A: Physicochemical and Engineering Aspects 584: 1–9. 10.1016/j.colsurfa.2019.124024.

[ptr70380-bib-0210] Spiridon, I. , and N. Anghel . 2025. “Cyclodextrins as Multifunctional Platforms in Drug Delivery and Beyond: Structural Features, Functional Applications, and Future Trends.” Molecules 30, no. 14: 1–42. 10.3390/molecules30143044. PMC12299055 40733310

[ptr70380-bib-0211] Stefaniu, A. , and L. C. Pirvu . 2022. “In Silico Study Approach on a Series of 50 Polyphenolic Compounds in Plants; A Comparison on the Bioavailability and Bioactivity Data.” Molecules 27, no. 4: 1–25. 10.3390/molecules27041413. PMC8878759 35209203

[ptr70380-bib-0212] Su, H. , Y. T. Ruan , Y. Li , J. G. Chen , Z. P. Yin , and Q. F. Zhang . 2020. “In Vitro and In Vivo Inhibitory Activity of Taxifolin on Three Digestive Enzymes.” International Journal of Biological Macromolecules 150: 31–37. 10.1016/j.ijbiomac.2020.02.027. 32035149

[ptr70380-bib-0213] Su, S. , Y. Wang , L. Bai , et al. 2015. “Structural Elucidation of In Vivo Metabolites of Isobavachalcone in Rat by LC‐ESI‐MSn and LC‐NMR.” Journal of Pharmaceutical and Biomedical Analysis 104: 38–46. 10.1016/j.jpba.2014.11.010. 25459757

[ptr70380-bib-0214] Subpart B—Food Additive Safety . 2004. “21 CFR Part 170.”

[ptr70380-bib-0215] Tai, J. , M. Han , D. Lee , I. H. Park , S. H. Lee , and T. H. Kim . 2022. “Different Methods and Formulations of Drugs and Vaccines for Nasal Administration.” Pharmaceutics 14, no. 5: 1–19. 10.3390/pharmaceutics14051073. PMC9144811 35631663

[ptr70380-bib-0216] Talavéra, S. , C. Felgines , O. Texier , et al. 2006. “Bioavailability of a Bilberry Anthocyanin Extract and Its Impact on Plasma Antioxidant Capacity in Rats.” Journal of the Science of Food and Agriculture 86, no. 1: 90–97. 10.1002/jsfa.2327.

[ptr70380-bib-0217] Tamizmaran, V. , V. S. Mannur , R. Koli , and P. Biradar . 2025. “Novel Intranasal Fisetin‐Loaded Mucoadhesive Microemulsion for Schizophrenia Management: A Nanotherapeutic Approach to Enhance Brain Bioavailability and Improved Efficacy.” Molecular Pharmaceutics 22, no. 7: 4293–4313. 10.1021/acs.molpharmaceut.5c00584. 40545922

[ptr70380-bib-0218] Tartari, A. P. S. , J. Jacumazo , A. K. P. Lorenzett , R. A. de Freitas , and R. M. Mainardes . 2025. “Development and Characterization of Silibinin‐Loaded Nanoemulsions: A Promising Mucoadhesive Platform for Enhanced Mucosal Drug Delivery.” Pharmaceutics 17, no. 2: 1–18. 10.3390/pharmaceutics17020192. PMC11859180 40006559

[ptr70380-bib-0219] Tehoharides, T. 2006. “Anti‐Inflammatory Compositions for Treating Multiple Sclerosis (Patent US20060013905A1).”

[ptr70380-bib-0220] Theoharides, T. C. 2009. “Method for Protecting Humans Against Superficial Vasodilator Flush Syndrome (Patent US7923043B2).”

[ptr70380-bib-0221] Tian, F. , Y. Zhu , H. Long , et al. 2002. “Liquid Chromatography Coupled With Multi‐Channel Electrochemical Detection for the Determination of Daidzin in Rat Blood Sampled by an Automated Blood Sampling System.” Journal of Chromatography. B, Analytical Technologies in the Biomedical and Life Sciences 772, no. 1: 173–177. https://www.elsevier.com/locate/chromb. 12016029 10.1016/s1570-0232(02)00087-9

[ptr70380-bib-0222] Tratnjek, L. , N. Sibinovska , K. Kristan , and M. E. Kreft . 2021. “In Vitro Ciliotoxicity and Cytotoxicity Testing of Repeated Chronic Exposure to Topical Nasal Formulations for Safety Studies.” Pharmaceutics 13, no. 11: 1–18. 10.3390/pharmaceutics13111750. PMC8618987 34834166

[ptr70380-bib-0223] Troelsen, J. T. , C. Mitchelmore , N. Spodsberg , A. M. Jensen , and H. Sjo= Stro= . 1997. “Regulation of Lactase‐Phlorizin Hydrolase Gene Expression by the Caudal‐Related Homoeodomain Protein Cdx‐2.” Biochemical Journal 322: 833–838. 9148757 10.1042/bj3220833 PMC1218263

[ptr70380-bib-0224] Türker, S. , E. Onur , and Y. Özer . 2004. “Nasal Route and Drug Delivery Systems.” Pharmacy World and Science: PWS 26, no. 3: 137–142. 15230360 10.1023/b:phar.0000026823.82950.ff

[ptr70380-bib-0225] Turner, R. B. , S. L. Fowler , and K. Berg . 2004. “Treatment of the Common Cold With Troxerutin.” APMIS 112, no. 9: 605–611. 10.1111/j.1600-0463.2004.apm1120908.x. 15601310

[ptr70380-bib-0226] Ullah, H. , and H. Khan . 2018. “Anti‐Parkinson Potential of Silymarin: Mechanistic Insight and Therapeutic Standing.” Frontiers in Pharmacology 9: 422. 10.3389/fphar.2018.00422. 29755356 PMC5934474

[ptr70380-bib-0227] Urpi‐Sarda, M. , M. Monagas , N. Khan , et al. 2009. “Epicatechin, Procyanidins, and Phenolic Microbial Metabolites After Cocoa Intake in Humans and Rats.” Analytical and Bioanalytical Chemistry 394, no. 6: 1545–1556. 10.1007/s00216-009-2676-1. 19333587

[ptr70380-bib-0228] van Breemen, R. B. , Y. Yuan , S. Banuvar , et al. 2014. “Pharmacokinetics of Prenylated Hop Phenols in Women Following Oral Administration of a Standardized Extract of Hops.” Molecular Nutrition and Food Research 58, no. 10: 1962–1969. 10.1002/mnfr.201400245. 25045111 PMC4265473

[ptr70380-bib-0229] Vanzo, A. , M. Scholz , M. Gasperotti , et al. 2013. “Metabonomic Investigation of Rat Tissues Following Intravenous Administration of Cyanidin 3‐Glucoside at a Physiologically Relevant Dose.” Metabolomics 9, no. 1: 88–100. 10.1007/s11306-012-0430-8.

[ptr70380-bib-0230] Văruț, R. M. , A. I. S. Popescu , S. Gaman , et al. 2025. “Cyclodextrin‐Based Drug Delivery Systems for Depression: Improving Antidepressant Bioavailability and Targeted Central Nervous System Delivery.” Pharmaceutics 17, no. 3: 1–24. 10.3390/pharmaceutics17030355. PMC11945394 40143019

[ptr70380-bib-0231] Vissiennon, C. , K. Nieber , O. Kelber , and V. Butterweck . 2012. “Route of Administration Determines the Anxiolytic Activity of the Flavonols Kaempferol, Quercetin and Myricetin ‐ Are They Prodrugs?” Journal of Nutritional Biochemistry 23, no. 7: 733–740. 10.1016/j.jnutbio.2011.03.017. 21840194

[ptr70380-bib-0232] Walle, T. 2004. “Absorption and Metabolism of Flavonoids.” Free Radical Biology and Medicine 36, no. 7: 829–837. 10.1016/j.freeradbiomed.2004.01.002. 15019968

[ptr70380-bib-0233] Walle, T. 2007. “Methylation of Dietary Flavones Greatly Improves Their Hepatic Metabolic Stability and Intestinal Absorption.” Molecular Pharmaceutics 4, no. 6: 826–832. 10.1021/mp700071d. 17958394

[ptr70380-bib-0234] Wang, H. , L. He , B. Liu , et al. 2018. “Establishment and Comparison of Air‐Liquid Interface Culture Systems for Primary and Immortalized Swine Tracheal Epithelial Cells.” BMC Cell Biology 19, no. 1: 1–10. 10.1186/s12860-018-0162-3. 29954317 PMC6025731

[ptr70380-bib-0235] Wang, Z. , S. O. Ka , Y. Lee , B. H. Park , and E. J. Bae . 2017. “Butein Induction of HO‐1 by p38 MAPK/Nrf2 Pathway in Adipocytes Attenuates High‐Fat Diet Induced Adipose Hypertrophy in Mice.” European Journal of Pharmacology 799: 201–210. 10.1016/j.ejphar.2017.02.021. 28213287

[ptr70380-bib-0236] Wasowski, C. , L. M. Loscalzo , J. Higgs , and M. Marder . 2012. “Chronic Intraperitoneal and Oral Treatments With Hesperidin Induce Central Nervous System Effects in Mice.” Phytotherapy Research 26, no. 2: 308–312. 10.1002/ptr.3560. 21717517

[ptr70380-bib-0237] Wengst, A. , and S. Reichl . 2010. “RPMI 2650 Epithelial Model and Three‐Dimensional Reconstructed Human Nasal Mucosa as In Vitro Models for Nasal Permeation Studies.” European Journal of Pharmaceutics and Biopharmaceutics 74, no. 2: 290–297. 10.1016/j.ejpb.2009.08.008. 19733661

[ptr70380-bib-0238] Wu, C. , S. Cao , T. Hong , et al. 2017. “Taxifolin Inhibits Rat and Human 11β‐Hydroxysteroid Dehydrogenase 2.” Fitoterapia 121: 112–117. 10.1016/j.fitote.2017.07.004. 28709706

[ptr70380-bib-0239] Wu, J. , Q. Q. Zhong , T. Y. Wang , et al. 2021. “MS‐Based Metabolite Analysis of Two Licorice Chalcones in Mice Plasma, Bile, Feces, and Urine After Oral Administration.” Biomedical Chromatography 35, no. 3: 1–25. 10.1002/bmc.4998. 33037660

[ptr70380-bib-0240] Wüthrich, P. , M. Martenet , and P. Buri . 1994. “Effect of Formulation Additives Upon the Intranasal Bioavailability of a Peptide Drug.” Pharmaceutical Research 11, no. 2: 278–282. 8165188 10.1023/a:1018915710318

[ptr70380-bib-0241] Xu, S. , J. Yu , J. Zhan , L. Yang , L. Guo , and Y. Xu . 2017. “Pharmacokinetics, Tissue Distribution, and Metabolism Study of Icariin in Rat.” BioMed Research International 2017: 1–17. 10.1155/2017/4684962. PMC5702950 29259982

[ptr70380-bib-0242] Xu, Z. , C. Jiang , S. Fan , R. Yan , N. Xie , and C. Wu . 2019. “Comparative Pharmacokinetics of Naringin and Neohesperidin After Oral Administration of Flavonoid Glycosides From Aurantii Fructus Immaturus in Normal and Gastrointestinal Motility Disorders Mice.” Chinese Herbal Medicines 11, no. 3: 314–320. 10.1016/j.chmed.2019.03.011.

[ptr70380-bib-0243] Xue, C. , S. Jiang , J. Guo , D. Qian , J. Duan , and E. Shang . 2011. “Screening for In Vitro Metabolites of *Abelmoschus manihot* Extract in Intestinal Bacteria by Ultra‐Performance Liquid Chromatography/Quadrupole Time‐Of‐Flight Mass Spectrometry.” Journal of Chromatography. B, Analytical Technologies in the Biomedical and Life Sciences 879, no. 32: 3901–3908. 10.1016/j.jchromb.2011.10.043. 22119023

[ptr70380-bib-0244] Yang, H. W. , H. J. Kim , J. H. Park , J. M. Shin , and H. M. Lee . 2018. “Apigenin Alleviates TGF‐β1‐Induced Nasal Mucosa Remodeling by Inhibiting MAPK/NF‐kB Signaling Pathways in Chronic Rhinosinusitis.” PLoS One 13, no. 8: 1–16. 10.1371/journal.pone.0201595. PMC6116943 30161164

[ptr70380-bib-0245] Yang, Z. , W. Zhu , S. Gao , et al. 2010. “Simultaneous Determination of Genistein and Its Four Phase II Metabolites in Blood by a Sensitive and Robust UPLC‐MS/MS Method: Application to an Oral Bioavailability Study of Genistein in Mice.” Journal of Pharmaceutical and Biomedical Analysis 53, no. 1: 81–89. 10.1016/j.jpba.2010.03.011. 20378296 PMC3397253

[ptr70380-bib-0246] Yoshida, K. , T. Takabayashi , A. Kaneko , et al. 2021. “Baicalin Suppresses Type 2 Immunity Through Breaking Off the Interplay Between Mast Cell and Airway Epithelial Cell.” Journal of Ethnopharmacology 267, no. 3: 113492. 10.1016/j.jep.2020.113492. 33091489

[ptr70380-bib-0247] Yoshimura, M. , A. Sano , J. I. Kamei , and A. Obata . 2009. “Identification and Quantification of Metabolites of Orally Administered Naringenin Chalcone in Rats.” Journal of Agricultural and Food Chemistry 57, no. 14: 6432–6437. 10.1021/jf901137x. 19558184

[ptr70380-bib-0248] Young, J. M. , and M. E. Morris . 2007. “Pharmacokinetics and Bioavailability of the Bioflavonoid Biochanin A: Effects of Quercetin and EGCG on Biochanin A Disposition in Rats.” Molecular Pharmaceutics 4, no. 6: 865–872. 10.1021/mp7000928. 17970592

[ptr70380-bib-0249] Yu, J. , and Y. Xu . 2024. “Diosmetin, Methylated Flavonoid Mitigates Ovalbumin Induced Allergic Rhinitis in Mice by Attenuating Inflammatory Signaling Proteins.” Indian Journal of Pharmaceutical Education and Research 58, no. 2: 685–694. 10.5530/ijper.58.2.77.

[ptr70380-bib-0250] Yuan, D. , Y. Guo , F. Pu , et al. 2024. “Opportunities and Challenges in Enhancing the Bioavailability and Bioactivity of Dietary Flavonoids: A Novel Delivery System Perspective.” Food Chemistry 430: 1–14. 10.1016/j.foodchem.2023.137115. 37566979

[ptr70380-bib-0251] Zeng, X. , H. Yao , Y. Zheng , et al. 2020. “Tissue Distribution of Naringin and Derived Metabolites in Rats After a Single Oral Administration.” Journal of Chromatography. B, Analytical Technologies in the Biomedical and Life Sciences 1136: 1–6. 10.1016/j.jchromb.2019.121846. 31821965

[ptr70380-bib-0252] Zhang, M. , H. Tang , Y. Yuan , et al. 2023. “The Role of Indoor Microbiome and Metabolites in Shaping Children's Nasal and Oral Microbiota: A Pilot Multi‐Omic Analysis.” Metabolites 13: 1040. 10.3390/metabo13101040. 37887365 PMC10608577

[ptr70380-bib-0253] Zhang, Q. , Y. Niu , Y. Li , et al. 2025. “Meningeal Lymphatic Drainage: Novel Insights Into Central Nervous System Disease.” Signal Transduction and Targeted Therapy 10, no. 1: 1–40. 10.1038/s41392-025-02177-z. 40320416 PMC12050339

[ptr70380-bib-0254] Zhang, S. , K. Sagawa , R. D. Arnold , E. Tseng , X. Wang , and M. E. Morris . 2010. “Interactions Between the Flavonoid Biochanin A and P‐Glycoprotein Substrates in Rats: In Vitro and In Vivo.” Journal of Pharmaceutical Sciences 99, no. 1: 430–441. 10.1002/jps.21827. 19499569

[ptr70380-bib-0255] Zhang, S. , N. Smith , D. Schuster , et al. 2011. “Quercetin Increases CFTR Mediated Chloride Transport and Ciliary Beat Frequency: Therapeutic Implications for Chronic Rhinosinusitisrhinosinusitis.” American Journal of Rhinology and Allergy 25, no. 5: 307–312. 10.2500/ajra.2011.25.3643. 22186243 PMC3584334

[ptr70380-bib-0256] Zhang, X. , G. Su , Z. Shao , et al. 2025. “Rational Development of Fingolimod Nano‐Embedded Microparticles as Nose‐To‐Brain Neuroprotective Therapy for Ischemic Stroke.” Drug Delivery and Translational Research 15, no. 6: 2022–2047. 10.1007/s13346-024-01721-8. 39485637 PMC12037672

[ptr70380-bib-0257] Zhou, S. , Y. Hu , B. Zhang , et al. 2008. “Dose‐Dependent Absorption, Metabolism, and Excretion of Genistein in Rats.” Journal of Agricultural and Food Chemistry 56, no. 18: 8354–8359. 10.1021/jf801051d. 18710250

[ptr70380-bib-0258] Zhou, Y. j. , H. Wang , H. h. Sui , L. Li , C. l. Zhou , and J. j. Huang . 2016. “Inhibitory Effect of Baicalin on Allergic Response in Ovalbumin‐Induced Allergic Rhinitis Guinea Pigs and Lipopolysaccharide‐Stimulated Human Mast Cells.” Inflammation Research 65, no. 8: 603–612. 10.1007/s00011-016-0943-0. 27043920

[ptr70380-bib-0259] Zhu, H. , Y. Xiao , H. Guo , et al. 2021. “The Isoflavone Puerarin Exerts Anti‐Tumor Activity in Pancreatic Ductal Adenocarcinoma by Suppressing mTOR‐Mediated Glucose Metabolism.” Aging 13, no. 23: 25089–25105. https://www.aging‐us.com. 34863080 10.18632/aging.203725 PMC8714170

[ptr70380-bib-0260] Zhu, Y. , J. Wen , Y. Cao , et al. 2016. “Identification of 3′,4′ ‐Dimethoxy Flavonol‐3‐β‐D‐Glucopyranoside Metabolites in Rats by Liquid Chromatography‐Electrospray Ionization Ion Trap Mass Spectrometry.” Molecules 21, no. 4: 1–13. 10.3390/molecules21040470. PMC6273979 27070571

